# Reactivity and Selectivity of Iminium Organocatalysis Improved by a Protein Host

**DOI:** 10.1002/anie.201806850

**Published:** 2018-08-23

**Authors:** Alexander R. Nödling, Katarzyna Świderek, Raquel Castillo, Jonathan W. Hall, Antonio Angelastro, Louis C. Morrill, Yi Jin, Yu‐Hsuan Tsai, Vicent Moliner, Louis Y. P. Luk

**Affiliations:** ^1^ School of Chemistry, Main Building Cardiff University Cardiff CF10 3AT UK; ^2^ Department de Química Física i Analítica Universitat Jaume I 12071 Castelló Spain

**Keywords:** enzyme models, molecular dynamics, organocatalysis, proteins, supramolecular chemistry

## Abstract

There has been growing interest in performing organocatalysis within a supramolecular system as a means of controlling reaction reactivity and stereoselectivity. Here, a protein is used as a host for iminium catalysis. A pyrrolidine moiety is covalently linked to biotin and introduced to the protein host streptavidin for organocatalytic activity. Whereas in traditional systems stereoselectivity is largely controlled by the substituents added to the organocatalyst, enantiomeric enrichment by the reported supramolecular system is completely controlled by the host. Also, the yield of the model reaction increases over 10‐fold when streptavidin is included. A 1.1 Å crystal structure of the protein–catalyst complex and molecular simulations of a key intermediate reveal the chiral scaffold surrounding the organocatalytic reaction site. This work illustrates that proteins can be an excellent supramolecular host for driving stereoselective secondary amine organocatalysis.

Designing host‐guest systems for organocatalysis has increasingly interested chemists.^1^ A molecular host provides a specific environment that dictates both selectivity and reactivity of the organocatalysis, thereby providing additional means for reaction control. Nature provides such examples, including proteins. These proteins present a hydrophobic and inherently chiral scaffold favorable for organocatalysis.^2^ Similar concepts have been tested previously. Small, metal‐free reagents including flavin,^3^ thiazolium ylides,^4^ pyridoxamine,^5^ or selenocysteine^6^ have been added to a protein covalently for oxidation, as well as C−C and C−N bond formation reactions. Recently, a novel artificial enzyme was created using π‐stacking organocatalysts whose selectivity can be improved by modifying the protein scaffold.1a, 7 In contrast, iminium formation by a secondary amine has become a major mode of substrate activation in organocatalysis,^8^ but a protein‐based host for this type of catalysis has not been achieved.

Streptavidin (**Sav**) possesses a strong, noncovalent affinity to d‐biotin.^9^ Since **Sav** mostly binds to the ureido moiety of biotin, the valeric acid sidechain can be chemically modified for other applications, such as the production of artificial enzymes carrying either nonmetal1a, 7 or metal cofactors.^10^ Herein, we have adapted this technology and prepared **Sav**‐based hybrid catalysts carrying biotinylated secondary amine functionalities (Scheme 1). This work demonstrates that **Sav**, as a water‐soluble protein, can provide a microenvironment favorable for stereoselective secondary amine organocatalysis.

**Scheme 1:**
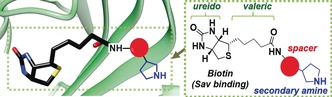
Model of the biotinylated organocatalysts anchored to the surface of streptavidin (**Sav**; PDB 1STP).

Several biotinylated organocatalysts were synthesized based on known catalytically active secondary amine motifs (Figure 1), including 4‐imidazolidinone (**1**–**4**), proline (**5** and **6**), and pyrrolidine (**7** and **8**) derivatives. The compounds **1**–**5** were prepared by copper‐catalyzed Huisgen 1,3‐dipolar cycloadditions of alkynylated biotin and azido‐functionalized precursors (5–47 % overall yield).^11^ The catalysts **6**–**8** were synthesized by direct coupling of biotin to either the Boc‐protected amino‐proline or amino‐pyrrolidine starting materials, followed by deprotection (48–63 % overall yield). The catalysts **1**–**3** and **5**–**8** were obtained as single stereoisomers, but an inseparable 1:1 mixture of *cis*‐ and *trans*‐isomers was formed for **4**.

**Figure 1:**
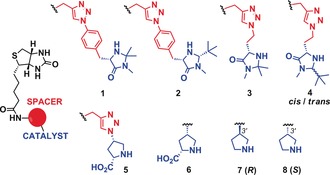
Biotinylated organocatalysts **1**–**8**.

The newly prepared biotinylated catalysts were used in the Michael addition of nitromethane to cinnamaldehyde (Table 1), a type of 1,4‐addition that is often used in pharmaceutical synthesis.^12^ Our previous analysis of this reaction indicates that water can hamper the reaction progress when used as a solvent, but enhances turnover rate when used as an additive.^13^ Hence, this sets the basis on how a protein scaffold can affect the efficiency of this organocatalytic reaction. For initial screening, each biotinylated catalyst (20 mol %) was incubated with cinnamaldehyde and five equivalents of nitromethane at 25 °C (see the Supporting Information). No activity was observed in either deionized water or in acidic buffer (pH 2.0–6.0). In contrast, at pH 7.0 and 8.0 the proline and pyrrolidine catalysts (**5**–**8**) were able to mediate the Michael addition as detected by GC‐MS, whereas such observations were absent for **1**–**4**. At pH 9.0, however, significant background reaction was observed, rendering these reaction conditions unfavorable for developing stereoselective organocatalysis. In an acidic (<pH 7.0) or a non‐buffered aqueous environment, secondary amines are likely to be protonated (p*K*_a_≈8) and deprotonation of nitromethane (p*K*_a_≈10) is unfavorable, and these factors likely stall the progress of the organocatalysis.

**Table 1:** Catalyst screening for the reaction of nitromethane with cinnamaldehyde.^[a]^ 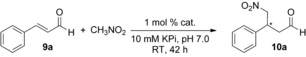

Entry	Catalyst	Solvent	Yield [%]^[b]^	TOF [*x* h^−1^]	e.r.^[c]^ (*S*/*R*)
1	None	KP_i_	3	–	–
2	Pyrrolidine	KP_i_	3	–	–
3	Proline	KP_i_	3	–	–
4	**Sav**‐biotin	KP_i_	3	–	–
5	**1‐4**	KP_i_	3	–	–
6	**5**	KP_i_	4	–	–
7	**6**	KP_i_	5	0.13	–
8	**7**	KP_i_	7	0.18	50:50
9	**8**	KP_i_	6	0.15	50:50
10	**Sav‐5**	KP_i_	6	0.15	–
11	**Sav‐6**	KP_i_	5	0.13	–
12	**Sav‐7**	KP_i_	30	0.76	80:20
13	**Sav‐8**	KP_i_	15	0.38	24:76
14	**Sav‐7** ^[d]^	KP_i_	36	0.28	80:20
15	**Sav‐7** ^[e]^	KP_i_/C_6_D_6_	<2		–
16	**Sav‐7** ^[e]^	KP_i_/CDCl_3_	<2		–
17	**Sav‐7** ^[e]^	KP_i_/THF^[f,g]^	34^[h]^		–
18	**Sav‐7** ^[e]^	KP_i_/EtOAc^[f]^	38^[h]^		92:8
19	**Sav‐7** ^[e]^	KP_i_/DMSO	<2		–
20	**Sav‐7** ^[e]^	KP_i_/MeCN	<2		–
21	**Sav‐7** ^[e]^	KP_i_/MeOH	80	4.4	91:9

[a] Reactions carried out for 42 h on a 3.3 μmol scale, using nitromethane (16.5 μmol), catalyst (1.0 mol %, additional 0.2 mol % **Sav**, 1.2 mol % active sites, for entries 4 and 10–14) in 500 μL of solvent. KP_i_=10 mm potassium phosphate buffer at pH 7.0 and 25 °C. [b] Conversion determined by ^1^H NMR spectroscopy for **5**–**8** and **Sav**‐**5**–**Sav**‐**8**. [c] Determined by chiral‐phase LC of the reduced product **11 a** (Chiralpak IB, see the Supporting Information). [d] Reaction was carried out at 4 °C for 136 h. [e] Reactions were carried out for 18 h instead of 42 h, using 1:1 mixtures of buffer to organic solvent. [f] Cinnamic acid was observed as side product. [g] Unidentified side products and the 1,2‐addition product were observed in significant amounts up to 50 %. [h] Range of yield observed in multiple runs in these solvents. DMSO=dimethyl sulfoxide, THF=tetrahydrofuran.

To see if the performance of organocatalysis can be improved by anchoring to a protein surface, the catalysts **5**–**8** were introduced to streptavidin (**Sav**). For the proline derivatives **5** and **6**, the reaction yields at pH 7.0 were not affected by the addition of **Sav** and remain at about 5–6 % (Table 1; see the Supporting Information). In contrast, the reactivities of the pyrrolidines **7** and **8** were significantly improved when **Sav** was included, showing about a five‐ and twofold increase, respectively, in the reaction yields at pH 7.0 (*k*_cat_/*k*_uncat_=10 and ΔΔ*G* ^≠^=5.71 kJ mol^−1^ for **Sav‐7**). No reaction enhancement was observed in control reactions with either l‐proline, pyrrolidine, or **Sav**‐biotin. This data highlights the synergistic effect observed by the introduction of **7** and **8** into the protein scaffold (Figure 2). While these two diastereomeric catalysts differ by one stereoconfiguration at the 3′‐position, the yield of the reaction catalyzed by **Sav‐7** is slightly higher. Indeed, the reactivity of **Sav‐7** is comparable to that of a water‐compatible derivative of the Jørgensen–Hayashi catalyst in that a similar reaction yield per mol % of catalyst was obtained.12c

**Figure 2:**
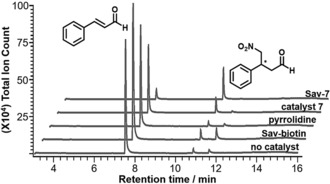
GC‐MS traces of the noncatalyzed reaction and reactions catalyzed by **Sav**‐**biotin**, pyrrolidine, **7**, and **Sav**‐**7** at pH 7.0.

Whereas many traditional organocatalysts such as the MacMillan and Jørgensen–Hiyashi catalysts contain either bulky or hydrogen‐bonding groups adjacent to the reacting nitrogen atom for stereoselectivity control, these substituents are absent in **7** and **8**. However, the transformation taking place on the surface of the inherently chiral **Sav** is anticipated to improve the stereoselectivity. This hypothesis is examined by chiral‐phase LC analyses of the catalytically enhanced reactions. In the absence of **Sav**, no enantioselectivity was observed. In the **Sav‐7** reaction, the majority of the product contains an *S* configuration at C3, giving an enantiomeric ratio of 80:20. Conversely, the *R* stereoisomer was preferentially formed in the **Sav‐8** reaction (Table 1, entries 12 and 13). These results clearly indicate that the binding of the catalysts to **Sav** causes noticeable improvement in enantioselectivity.

A crystal structure at a resolution of 1.1 Å was obtained to pinpoint the interactions between the secondary amine catalyst and protein scaffold. The amide bond in the valeric moiety undergoes hydrogen bonding with Ser88, which is frequently seen when biotin is functionalized by amide‐bond formation.10f,10h,10j In previous **Sav**‐based artificial metalloenzymes, the location of the metal catalyst is often unclear because either the ligand is inherently flexible or the metal catalyst dissociated during crystallography.10f,10h,10j In contrast, the location of the secondary amine catalyst is clearly revealed in this work. The pyrrolidinyl moiety forms a hydrogen bond with Ser112, which has been shown to be important in artificial metalloenzyme design.10f, 10h, 10j Other residues surrounding the organocatalytic reaction are also revealed, including Leu124 and Lys121. Notably, Lys121 was also found to play critical role in the development of the **Sav**‐based anion‐π enzyme dictating the activity of the catalytically important tertiary amine.1a However, this residue likely plays a different role here, as it is distant from the catalytic amine atom (8.1 and ca. 7.3 Å based on the X‐ray and MD simulations, respectively). Since Lys121 is within proximity to C3 and the phenyl moiety of the intermediate (4–5 Å), it likely dictates how the nucleophile approaches the iminium intermediate for reaction.

Molecular dynamics (MD) simulations were performed to investigate the origin of reaction stereoselectivity (see the Supporting Information for details). Our previous computational analysis indicates that the iminium and deprotonated nitromethane intermediates are formed in one step and thus they are extremely transient before forming a stereogenic center at C3.^13^ Hence, the reaction stereoselectivity is most likely dictated by the hemiaminal tetrahedral intermediate which forms prior to the iminium/deprotonated nitromethane pair. C1 of the hemiaminal tetrahedral intermediate can be either *R* or *S*, which exerts a strong effect on how C3 is being exposed for nucleophilic reaction (see Schemes S1and S2 in the Supporting Information). Interestingly, a representative snapshot of the (1*S*) intermediate from the MD simulation of **Sav**‐**7** overlays well with the crystal structure complex (Figure 3). According to the population analysis obtained from the MD simulations, this (1*S*) intermediate dictates the stereoselectivity of the reaction. Upon dehydration, this intermediate will be converted into the iminium intermediate which exposes the C3 *Si* face for nucleophilic addition, whereas the opposite face is shielded by the protein (Figure 4). Consequently, formation of the stereoisomer (*S*)‐**10 a** is favored. Similarly, for the iminium derived from the (1*R*) intermediate, the C3 *Si* face of the iminium intermediate is exposed for reaction. In the case where **8** is used, formation of the (1*R*) intermediate is favored and the product stereoisomer is reversed (see Figure S7). However, this intermediate is noticeably more flexible, and analysis of the MD trajectory of this complex indicates that while the majority of the conformers (ca. 90 %) yield the (3*R*) adduct, there is a population of conformers that yield the opposite stereoisomer (see geometrical analysis and animation movies deposited in the Supporting Information).

**Figure 3:**
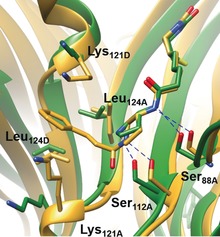
Overlay of the 1.1 Å crystal structure of **Sav‐7** (green) and the hemiaminal tetrahedral intermediates obtained from the MD simulations (yellow). Blue dashed lines denote proposed hydrogen‐bonding interactions.

**Figure 4:**
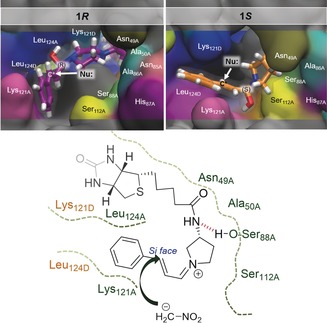
Top: Tetrahedral intermediates (1*R*) and (1*S*) intermediate derived from QM/MM studies. Bottom: Schematic representation of the orientation of the iminium intermediate. The lower‐case A denotes residues within one subunit and lower‐case D denotes residues from the other subunit. Red refers to hypothetical hydrogen‐bonding interactions.

Attempts to further enhance the reaction yield were made by prolonging the reaction time or raising the pH, but no improvement was found. Instead, there was a substantial amount of precipitation observed after 6–42 hours, and it can be caused either by imine formation between free amines of the protein and the aldehyde moieties or by aggregation resulting from surface binding of the hydrophobic reactants. To suppress the precipitation, different organic solvents were included as additives (Table 1). They can be separated into three categories: nonpolar (C_6_D_6_, CDCl_3_), polar aprotic (EtOAc, THF, DMSO, MeCN), and polar protic (MeOH) solvents.10i, 14 In all cases precipitate formation was suppressed but the reaction yield was improved only when either THF, EtOAc, or MeOH were included. However, reaction yields fluctuated severely when THF or EtOAc was used, and cinnamic acid was observed as a side product. Also, in THF up to 50 % of 1,2‐addition product was observed. The presence of MeOH enhances the reaction yield up to an average value of 80 % within 18 hours per mol % catalyst used. This data transforms into a rate enhancement (*k*_cat_/*k*_uncat_) of 8 and a ΔΔ*G* ^≠^=5.12 kJ mol^−1^ with 10 % additional side products observed in the uncatalyzed background reaction. Such efficiency has not been seen in previously reported aqueous organocatalytic system.12c, 15 Furthermore, while the previously reported **Sav**‐based π‐stacking organocatalytic system operates best at low pH,1a in the **Sav‐7** system such conditions were found to be detrimental for catalysis (ca. 5 % at pH 5.0 and no product observed at pH 3.0).

Since MeOH proved to be the most productive cosolvent, the ratio of this solvent to buffer was screened for optimal reaction yield and enantioselectivity. Increasing the amount of MeOH steadily enhances both the yield and enantioselectivity of the model reaction (see Figure S8). A ratio of 1:1 of buffer/MeOH at pH 7.0 proved to be the optimal reaction conditions. In the control experiment where the **Sav** protein scaffold is omitted, no enantioselectivity was observed. Together, these data indicate that the stereoselectivity originates from the binding between **Sav** and **7**, which remains intact even in the presence of a significant amount of MeOH.

The applicability of the **Sav‐7** system was explored by testing cinnamaldehyde derivatives and the respective ketones as alternative substrates (Table 2). Almost all of the employed aldehydes are tolerated by **Sav‐7**, giving acceptable to good yields (>35 %) and enantioselectivities (entries 1–7). The low yield of 4′‐nitrocinnamaldehyde (entry 5) is most likely caused by its low solubility in the buffer/MeOH mixture. In contrast, all of the ketone counterparts give poor yields, and only the ones shown in Table 2 afford a detectable yield.

**Table 2:** Exploration of substrate scope in the reaction of nitromethane with α,β‐unsaturated carbonyl compounds and **Sav‐7**.^[a]^ 
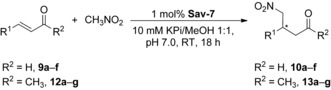

Entry	R^1^, R^2^	Yield [%]^[b]^	e.r.^[c]^
1	Ph, H; **9 a**	80	9:91
2	4′‐MeC_6_H_4_, H; **9 b**	37	13:87
3	4′‐OMeC_6_H_4_, H; **9 c**	39	14:86
4	4′‐ClC_6_H_4_, H; **9 d**	79	11:89
5	4′‐NO_2_C_6_H_4_, H; **9 e**	10	24:76
6^[d]^	3′‐Py, H; **9 f**	62	n.d.
7	4′‐XC_6_H_4_, CH_3_; (X=Cl; **12 d**, NO_2_; **12 e**, CF_3_; **12 g**)	<2–5	n.d.

[a] Reactions carried out for 18 h on a 3.3 μmol scale, using nitromethane (16.5 μmol), catalyst (1.0 mol %, additionally 0.2 mol % **Sav**, 1.2 mol % active sites) in 500 μL of mixed solvent, KP_i_/MeOH=1:1, KP_i_=10 mm, pH 7.0, and 25 °C. [b] Conversion determined by ^1^H NMR spectroscopy. [c] Determined by chiral‐phase LC of reduced products **11 a**–**11 f** (see the Supporting Information). [d] The corresponding product was found to be degraded during purification, and thus the e.r. value was not determined. n.d.=not determined.

In conclusion, a hybrid organocatalytic system based on the streptavidin‐biotin technology was developed in this work. This system facilitates secondary amine catalyzed reactions. Surprisingly, by simply exploiting the scaffold of the wild‐type streptavidin, a sparsely substituted cyclic secondary amine can be used to catalyze reactions with high enantioselectivity. This approach bypasses the need of striking a balance between reactivity and stereoselectivity as frequently seen in traditional organocatalyst design.^8^ Molecular simulations and protein crystallography have been particularly insightful, as they reveal how the protein dictates the orientation of the substrate and reaction stereoselectivity. This work lays the basis for protein engineering in which a designated scaffold can be modified for optimal organocatalysis.10d,10f,10g,10i–10k, 16 Furthermore, since this work enables organocatalysis in an isolated environment of a supramolecular complex, its compatibility with other species including reagents, other catalysts, and intermediates are greatly enhanced.1a,1c Hence, this protein‐based organocatalytic system will facilitate the development of tandem reaction sequences and hybrid catalysts as tools in chemical biology.^17^

## Conflict of interest

The authors declare no conflict of interest.

## Supporting information

As a service to our authors and readers, this journal provides supporting information supplied by the authors. Such materials are peer reviewed and may be re‐organized for online delivery, but are not copy‐edited or typeset. Technical support issues arising from supporting information (other than missing files) should be addressed to the authors.

Supplementary

Supplementary

Supplementary

Supplementary

Supplementary
