# Supplementary material for: Reactivity and Selectivity of Iminium Organocatalysis Improved by a Protein Host
Source: Angew Chem Int Ed Engl. 2018 Aug 23;57(38):12478–82. doi: 10.1002/anie.201806850 (PMC6531919; doi:10.1002/anie.201806850)
Supplement: Supplementary file 1 — Supplementary [file ANIE-57-12478-s001.pdf]

Angewandte Chemie
Eine Zeitschrift der Gesellschaft Deutscher Chemiker

Supporting Information

Reactivity and Selectivity of Iminium Organocatalysis Improved by a Protein Host

Alexander R. Nödling, Katarzyna Świderek, Raquel Castillo, Jonathan W. Hall, Antonio Angelastro, Louis C. Morrill, Yi Jin, Yu-Hsuan Tsai, Vicent Moliner, and Louis Y. P. Luk**

anie_201806850_sm_miscellaneous_information.pdf
anie_201806850_sm_Sav-7-R.avi
anie_201806850_sm_Sav-7-S.avi
anie_201806850_sm_Sav-8-R.avi
anie_201806850_sm_Sav-8-S.avi

Supporting Information

Table of Contents

1 General Information 6

2 Experimental Details for the Preparation of Catalysts 1–8 8

2.1 Biotinylating Reagents 8

(+)-Biotin propargyl amide 8

(+)-Biotin NHS ester 8

2.2 5-((3a*S*,4*S*,6a*R*)-2-oxohexahydro-1H-thieno[3,4-d]imidazol-4-yl)-N-((1-(4-(((*S*)-1,2,2-trimethyl-5-oxoimidazolidin-4-yl)methyl)phenyl)-1H-1,2,3-triazol-4-yl)methyl)pentanamide 1 9

(*S*)-4-Azidophenylalanine methyl ester hydrochloride 9

(*S*)-4-Azidophenylalanine methyl amide 9

(*S*)-5-(4-Azidobenzyl)-2,2,3-trimethylimidazolidin-4-one 10

5-((3a*S*,4*S*,6a*R*)-2-oxohexahydro-1H-thieno[3,4-d]imidazol-4-yl)-*N*-((1-(4-(((*S*)-1,2,2-trimethyl-5-oxoimidazolidin-4-yl)methyl)phenyl)-1H-1,2,3-triazol-4-yl)methyl)pentanamide 1 10

2.3 *N*-((1-(4-(((2*S*,4*S*)-2-(*tert*-butyl)-1-methyl-5-oxoimidazolidin-4-yl)methyl)phenyl)-1H-1,2,3-triazol-4-yl)methyl)-5-((3a*S*,4*S*,6a*R*)-2-oxohexahydro-1H-thieno[3,4-d]imidazol-4-yl)pentanamide 2 11

(2*S*,5*S*)-5-(4-azidobenzyl)-2-(*tert*-butyl)-3-methylimidazolidin-4-one 11

N-((1-(4-(((2*S*,4*S*)-2-(*tert*-butyl)-1-methyl-5-oxoimidazolidin-4-yl)methyl)phenyl)-1H-1,2,3-triazol-4-yl)methyl)-5-((3a*S*,4*S*,6a*R*)-2-oxohexahydro-1H-thieno[3,4-d]imidazol-4-yl)pentanamide 2 12

2.4 5-((3a*S*,4*S*,6a*R*)-2-oxohexahydro-1H-thieno[3,4-d]imidazol-4-yl)-*N*-((1-(2-((*S*)-1,2,2-trimethyl-5-oxoimidazolidin-4-yl)ethyl)-1H-1,2,3-triazol-4-yl)methyl)pen-tanamide 3 13

(*S*)-2-Amino-4-azidobutanoic acid methyl ester hydrochloride 13

(*S*)-*N*-Methyl-2-amino-4-azidobutanamide 13

(*S*)-5-(2-Azidoethyl)-2,2,3-trimethylimidazolidin-4-one 14

5-((3a*S*,4*S*,6a*R*)-2-oxohexahydro-1H-thieno[3,4-d]imidazol-4-yl)-*N*-((1-(2-((*S*)-1,2,2-trimethyl-5-oxoimidazolidin-4-yl)ethyl)-1H-1,2,3-triazol-4-yl)methyl)pentanamide 3 14

2.5 *N*-((1-(2-((4*S*)-2-(*tert*-butyl)-1-methyl-5-oxoimidazolidin-4-yl)ethyl)-1H-1,2,3-triaz-ol-4-yl)methyl)-5-((3a*S*,4*S*,6a*R*)-2-oxohexahydro-1H-thieno[3,4-d]imidazol-4-yl)pentanamide 4 15

(5*S*)-5-(2-azidoethyl)-2-(*tert*-butyl)-3-methylimidazolidin-4-one 15

N-((1-(4-(((2*S*,4*S*)-2-(*tert*-butyl)-1-methyl-5-oxoimidazolidin-4-yl)methyl)phenyl)-1H-1,2,3-triazol-4-yl)methyl)-5-((3a*S*,4*S*,6a*R*)-2-oxohexahydro-1H-thieno[3,4-d]imidazol-4-yl)pentanamide 4 16

2.6 (2*S*,4*S*)-2-carboxy-4-(4-((5-((3a*S*,4*S*,6a*R*)-2-oxohexahydro-1H-thieno[3,4-d]imidazol-4-yl)pentanamido)methyl)-1H-1,2,3-triazol-1-yl)pyrrolidin-1-ium trifluoroacetate 5 17

(2*S*,4*S*)-1-(*tert*-butoxycarbonyl)-4-(4-((5-((3a*S*,4*S*,6a*R*)-2-oxohexahydro-1H-thieno[3,4-d]imidazol-4-yl)pentanamido)methyl)-1H-1,2,3-triazol-1-yl)pyrrolidine-2-carboxylic acid 17

(2*S*,4*S*)-2-carboxy-4-(4-((5-((3a*S*,4*S*,6a*R*)-2-oxohexahydro-1H-thieno[3,4-d]imidazol-4-yl)pentanamido)methyl)-1H-1,2,3-triazol-1-yl)pyrrolidin-1-ium trifluoroacetate 5 18

2.7 (2*S*,4*S*)-2-carboxy-4-(5-((3a*S*,4*S*,6a*R*)-2-oxohexahydro-1H-thieno[3,4-d]imidazol-4-yl)pentanamido)pyrrolidin-1-ium formate 6 19

2.8 (*R*)-3-(5-((3a*S*,4*S*,6a*R*)-2-oxohexahydro-1H-thieno[3,4-d]imidazol-4-yl)pentanamido)pyrrolidin-1-ium formate 7 20

2.9 (*S*)-3-(5-((3a*S*,4*S*,6a*R*)-2-oxohexahydro-1H-thieno[3,4-d]imidazol-4-yl)pentanamido)pyrrolidin-1-ium formate 8 21

3 Experimental Details for the Activity Screening of Catalysts 1–7 in the Michael Addition of Nitromethane to Cinnamaldehyde 22

3.1 GCMS Based Screening for Catalyst Activity 22

4 Experimental Details for the Activity Screening of Catalysts 1–7 and Sav in the Michael Addition of Nitromethane to Cinnamaldehyde 23

4.1 GCMS Based Screening for Hybrid Catalyst Activity 23

Pure Catalyst Runs 23

Organocatalytic Artificial Enzyme Runs 23

4.2 ^{1}H NMR Based Screening for Yield Determination 24

Catalytic Runs – KP_i Buffer 24

Catalytic Runs – Organic Solvents 24

Exemplary ^{1}H NMR Spectrum after Extraction of the Buffer Reaction Mixture 26

Exemplary ^{1}H NMR Spectrum after Extraction of the Biphasic Reaction Mixture 26

5 Experimental Details for the Catalyzed Michael Addition of Nitromethane to Cinnamaldehyde for Chiral Analysis 27

5.1 Half-Micro Scale Reactions for Product Isolation and Analysis 27

6 Experimental Details for the Structure Determination of Sav-7 28

6.1 Procedure for Crystallization of Sav-7 28

6.3 X-ray data collection, processing and structure solution 28

6.4 Crystallographic Details for the Obtained Crystal Structure of Sav-7 29

7 Experimental Details for the Molecular Dynamics Simulations of the Model Michael Reaction with Sav-7 and Sav-8 30

7.1 Computer simulations of the active catalysts Sav-7 and Sav-8 30

8 NMR Spectra of Novel Compounds 40

8.1 (S)-4-Azidophenylalanine methyl amide 40

8.2 (*S*)-5-(4-Azidobenzyl)-2,2,3-trimethylimidazolidin-4-one 41

8.3 5-((3a*S*,4*S*,6a*R*)-2-oxohexahydro-1H-thieno[3,4-d]imidazol-4-yl)-N-((1-(4- (((*S*)-1,2,2-trimethyl-5-oxoimidazolidin-4-yl)methyl)phenyl)-1H-1,2,3-triazol-4-yl)methyl)pentanamide 1 42

8.4 (2*S*,5*S*)-5-(4-azidobenzyl)-2-(*tert*-butyl)-3-methylimidazolidin-4-one 43

8.5 *N*-((1-(4-(((2*S*,4*S*)-2-(*tert*-butyl)-1-methyl-5-oxoimidazolidin-4-yl)methyl)phenyl)-1H-1,2,3-triazol-4-yl)methyl)-5-((3a*S*,4*S*,6a*R*)-2-oxohexahydro-1H-thieno[3,4-d]imidazol-4-yl)pentanamide 2 44

8.6 (*S*)-*N*-Methyl-2-amino-4-azidobutanamide 45

8.7 (*S*)-5-(2-Azidoethyl)-2,2,3-trimethylimidazolidin-4-one 46

8.8 5-((3a*S*,4*S*,6a*R*)-2-oxohexahydro-1H-thieno[3,4-d]imidazol-4-yl)-*N*-((1-(2-((*S*)-1,2,2-trimethyl-5-oxoimidazolidin-4-yl)ethyl)-1H-1,2,3-triazol-4-yl)methyl)pentanamide 3 47

8.9 (5*S*)-5-(2-azidoethyl)-2-(*tert*-butyl)-3-methylimidazolidin-4-one 48

8.10 *N*-((1-(4-(((2*S*,4*S*)-2-(*tert*-butyl)-1-methyl-5-oxoimidazolidin-4-yl)methyl)phenyl)-1H-1,2,3-triazol-4-yl)methyl)-5-((3a*S*,4*S*,6a*R*)-2-oxohexahydro-1H-thieno[3,4-d]imidazol-4-yl)pentanamide 4 49

8.11 (2*S*,4*S*)-1-(*tert*-butoxycarbonyl)-4-(4-((5-((3a*S*,4*S*,6a*R*)-2-oxohexahydro-1H-thieno[3,4-d]imidazol-4-yl)pentanamido)methyl)-1H-1,2,3-triazol-1-yl)pyrrolidine-2-carboxylic acid 50

8.12 (2*S*,4*S*)-2-carboxy-4-(4-((5-((3a*S*,4*S*,6a*R*)-2-oxohexahydro-1H-thieno[3,4-d]imidazol-4-yl)pentanamido)methyl)-1H-1,2,3-triazol-1-yl)pyrrolidin-1-ium trifluoroacetate 5 51

8.13 (2*S*,4*S*)-2-carboxy-4-(5-((3a*S*,4*S*,6a*R*)-2-oxohexahydro-1H-thieno[3,4-d]imidazol-4-yl)pentanamido)pyrrolidin-1-ium formate 6 51

8.14 (*R*)-3-(5-((3a*S*,4*S*,6a*R*)-2-oxohexahydro-1H-thieno[3,4-d]imidazol-4-yl)pentanamido)pyrrolidin-1-ium formate 7 53

8.15 (*S*)-3-(5-((3a*S*,4*S*,6a*R*)-2-oxohexahydro-1H-thieno[3,4-d]imidazol-4-yl)pentanamido)pyrrolidin-1-ium formate 8 54

9 GCMS Traces of Catalyst Activity Screening 55

9.1 pH screening of catalyst 5 55

9.2 pH screening of catalyst 6 56

9.3 pH screening of catalyst 7 57

10 GCMS Traces of Hybrid Catalyst Activity Screening 58

10.1 Reactions at pH 7.0, 10 mM KP_i, 25 °C, 18 h 58

10.2 Reactions at pH 8.0, 10 mm KP_i, 25 °C, 18 h 60

10.3 Reactions at pH 9.0, 10 mM Tris, 25 °C, 18 h 62

11 Table of Optimized Catalytic Runs for Yield Determination 64

11.1 Catalytic Runs – KP_i Buffer 64

12 Chiral HPLC Data of Micro-Scale Reactions 66

12.1 Cinnamaldehyde Runs under Different Conditions 66
Racemate 11a 66
Product 11a with Catalyst 7, Pure Buffer 67
Product 11a with Catalyst 8 68
Product 11a with Catalyst Sav-7 69
Product 11a with Catalyst Sav-8 70
Product 11a with Catalyst L-14 71
Product 11a with Catalyst Sav-7, Ethylacetate 72
Product 11a with Catalyst Sav-7, 50% Methanol 73
Product 11a with Catalyst Sav-7, 25% Methanol 74
Product 11a with Catalyst Sav-7, 10% Methanol 75
12.2 Cinnamaldehyde Derivatives under Optimized Conditions 76
Product 11b with Catalyst Sav-7, Methanol 76
Racemate 11b 77
Product 11c with Catalyst Sav-7, Methanol 78
Racemate 11c 79
Product 11d with Catalyst Sav-7, Methanol 80
Racemate 11d 81
Product 11e with Catalyst Sav-7, Methanol 82
Racemate 11e 83
13 References 84

1 General Information

Reactions were performed in oven dried glassware without precautions to exclude air. Reaction temperatures are stated as heating device temperature (e.g. oil bath, shaker, etc.), if not stated otherwise. Concentrations under reduced pressure were performed by rotary evaporation at 40°C at the appropriated pressure, unless otherwise noted. Cinnamaldehyde was purified by distillation under reduced pressure (about 5 mbar) and stored under inert atmosphere at –23 °C. Cinnamaldehyde derivatives **9b–9f** were obtained commercially (Sigma-Aldrich, Acros, Fluorochem, Santa Cruz Biotechnology) and used without further purification or synthesized according to a literature procedure in case of **9f**.[1] Ketones **12a–12g** were obtained commercially or synthesized according to literature procedures.[2] **12f** was not used in this study. Deionized water was obtained by an *Elga PURELAB Option* system (15 MΩ·cm). Amberlite IR-120 hydrogen form ion exchange resin was stirred in MeOH for several minutes, filtered, washed with MeOH, and dried under fine vacuum. Jørgensen-Hayashi catalysts **L-14** and **D-14** were prepared according to known literature procedures.[3] Enantioenriched samples of (*S*)- and (*R*)-**10a** for peak assignment in chiral HPLC measurements were obtained using **L-14** and **D-14** according to a known procedure.[4] Racemic samples of **10a–10f** were obtained following a known procedure, using piperidine as catalyst.[4] All other solvents and reagents were obtained from commercial sources and used as received.

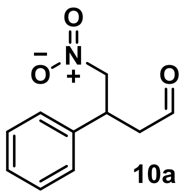

10a

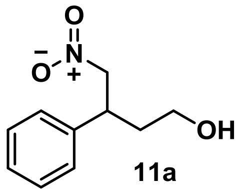

11a

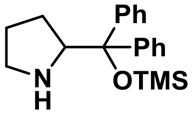

14

Sav (Streptavidin Streptomyces Avidinii Recombinant, tetramer, M_w ≈ 52 kDa) was obtained commercially from *ProSpec* (PRO-791) as lyophilized powder in 10 mM KP_i pH 6.5 and stored at −80 °C upon receipt until further use. According to the supplier **Sav** consists of the following amino acid sequence:

```
MAEAGITGTWYNQLGSTFIVTAGADGALTGTYESAVGNAESRYVLT
GRYDSAPATDGSGTALGWTVAWKNNYRNAHSATTWSGQYVGGA
EARINTQWLLTSGTTEANAWKSTLVGHDTFTKVKPSAAS
```

A *Hanna instruments pH 209* benchtop pH Meter connected to a *Jenway* micro pH electrode or a *VWR Universal* pH electrode were used for the pH adjustment of buffers and reaction mixtures employing either 1.0 M or 0.1 M sodium hydroxide solution or hydrochloric acid.

Shaking of the reactions at 25 °C was achieved using a *VWR* thermoshaker *Mini shake lite* or a *VWR Incubating Orbital Shaker*.

Analytical and preparative Thin Layer Chromatography (TLC) was carried out with silica gel 60 F254 aluminum sheets from *Merck*. Detection was carried out using UV light (λ = 254 nm

and 366 nm), followed by immersion in permanganate or cerium ammonium molybdate staining solution with subsequent development via careful heating with a heat gun. Flash column chromatography was performed using silica gel (pore size 60 Å, 0.040-0.063 mm).

^{1}H and ^{13}C NMR spectra were recorded in $CDCl_3$, methanol-d_4, DMSO-d_6, or D_2O on *Bruker Fourier 300*, *Ultrashield 400*, or *Ascend 500* instruments. Chemical shifts are reported in parts per million (ppm) and are referenced to the residual solvent resonance as the internal standard ($CHCl_3$: δ = 7.26 ppm for ^{1}H and $CDCl_3$: δ = 77.2 ppm for ^{13}C NMR; methanol-d_3: δ = 3.31 ppm for ^{1}H and methanol-d_4: δ = 49.0 ppm for ^{13}C NMR; DMSO-d_5: δ = 2.50 ppm for ^{1}H and DMSO-d_6: δ = 39.5 ppm for ^{13}C NMR; D_2O: δ = 4.79 ppm for ^{1}H).[5] Data are reported as follows: chemical shift, multiplicity (br s = broad singlet, s = singlet, d = doublet, t = triplet, q = quartet, p = pentet, sept = septet, br m = broad multiplet, m = multiplet, m_c = centrosymmetric multiplet), coupling constants (Hz) and integration.

High resolution Mass spectra (HRMS) were recorded on a *Waters LCT Premier* (ESI-(+) and APCI-(+)) or a *Waters GCT Premier* (EI) system.

Gas chromatography–mass spectrometry (GCMS) was performed on an *Agilent 6890N* system with a *Waters GCT Premium* EI MS instrument employing an *Agilent DB-35* column, 30 m, 350 µm inner diameter, 0.25 µm film thickness, and an inlet temperature of 150 °C. If not stated otherwise the following method was employed: 80 °C initial temperature with a 15 °C/min ramp until 250 °C.

Liquid chromatography–mass spectrometry (LCMS) was performed on a *Dionex Ultimate 3000* LC system with a *Bruker Amazon LS* MS instrument employing a *Waters Acquity BEH C18* column, 130 Å, 1.7 µm particle size, 2.1 mm × 100 mm. If not stated otherwise the following method was employed: 40 °C, flow rate = 0.3 mL·min^{-1}, H_2O:MeCN (0.1% HCO_2H) 97:3, 1 min, gradient to 3:97 over 18 min, 1 min hold, gradient to 97:3 over 1 min.

Preparative reverse phase high performance liquid chromatography (RP-HPLC) was performed on a *Dionex* LC system consisting of a *Dionex UVD17OU* detector, *UCI-50*, and *P680* HPLC pump using a *Supelcosil PLC-18* column, 12 µm particle size, 21.2 mm × 250 mm. If not stated otherwise the following method was employed: rt, flow rate = 10.0 mL·min^{-1}, H_2O:MeCN (0.1% HCO_2H) 99:1, gradient to 50:50 over 35 min.

Analytical chiral HPLC analysis of product **11a** was performed on an *Agilent Technologies 1260 Infinity Quaternary* LC system using a *Chiralpak IB* column, 4.6 mm × 250 mm (0.5 mL/min, 20 °C, *n*-hexane/*iso*-propanol 90:10). Products **11b–11e** were analysed on a *Gilson* HPLC system composed of a *Gilson 305* pump, a *Gilson 306* pump, a *Gilson 811C* dynamic mixer, a *Gilson 805* manometric module, a *Gilson 401C* dilutor, a *Gilson 213XL* sample injector, and a *Gilson 118* UV-Vis detector, using a *Chiralpak IC* column. Detailed conditions and retention times are given in section 12. The absolute stereochemistry of **11a** was assigned according to Ref [3], or in analogy to **11a** for **11b–11e**.

2 Experimental Details for the Preparation of Catalysts 1–8

2.1 Biotinylating Reagents

(+)-Biotin propargyl amide

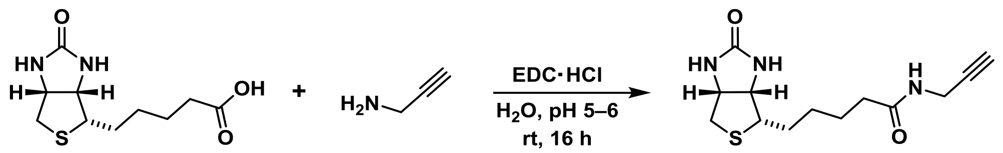

A mixture of (+)-biotin (140 mg, 0.57 mmol, 1.0 eq) and propargylamine (65 mg, 1.18 mmol, 2.0 eq) in water (10 mL) at room temperature was acidified to pH 5.0-6.0 by dropwise addition of 2.0 M HCl. After the addition of 1-ethyl-3-(3-dimethylaminopropyl)carbodiimide hydrochloride (EDC·HCl, 210 mg, 1.09 mmol, 1.9 eq) the mixture was stirred at room temperature for 16 h. The solution was concentrated under reduced pressure and the residue was purified via flash column chromatography ($CHCl_3$:MeOH 83:17) to give the alkyne as a white solid (125 mg, 0.44 mmol, 77% yield).

^{1}H NMR (400 MHz, DMSO-*d*$_6$): δ = 8.25 (br m, 1H), 6.44 (s, 1H), 6.37 (s, 1H), 4.34–4.26 (m, 1H), 4.13 (s, 1H), 3.87–3.81 (m, 2H), 3.10 (s, 2H), 2.82 (dd, *J* = 12.5, 4.3 Hz, 1H), 2.58 (d, *J* = 12.5 Hz, 1H), 2.08 (*t*, *J* = 7.1 Hz, 2H),1.66–1.24 (m, 6H) ppm.
The analytical data is in accordance with the literature.[6]

(+)-Biotin NHS ester

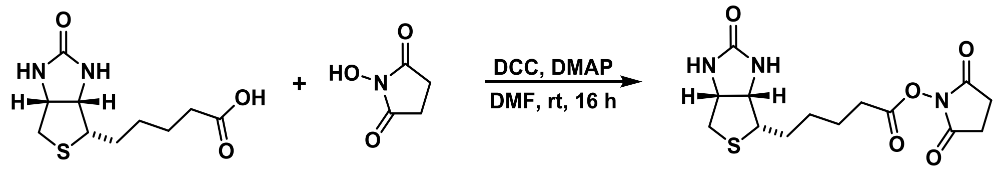

(+)-Biotin (960 mg, 4.0 mmol, 1.0 eq), *N*-hydroxysuccinimide (NHS, 920 mg, 8.0 mmol, 2.0 eq), and 2-(dimethylamino)pyridine (DMAP, 24 mg, 0.2 mmol, 0.05 eq) were dissolved in dry DMF (≈ 40 mL) under inert atmosphere. The solution was cooled to 0 °C with an ice bath. Dicyclohexylcarbodiimide (DCC, 908 mg, 4.4 mmol, 1.1 eq) dissolved in dry DMF was then added dropwise. The reaction mixture was left stirring at room temperature overnight and formed precipitate was removed via vacuum filtration. Crude NHS ester was precipitated by the addition of Et_2O, collected by filtration, and washed with deionized water and Et_2O. The crude product was recrystallized from *iso*-propanol, collected via filtration, and washed with Et_2O to afford the active ester as white solid after drying under fine vacuum (272 mg, 0.8 mmol, 20% yield).

^{1}H NMR (400 MHz, DMSO-*d*$_6$): δ = 6.43 (s, 1H), 6.37 (s, 1H), 4.32 (m_c, 1H), 4.13 (m_c, 1H),), 3.10 (m_c, 1H), 2.87−2.78 (m, 5H), 2.67 (t, *J* = 7.3 Hz, 1H), 2.58 (d, *J* = 12.6 Hz, 1H),1.71 – 1.28 (m, 6H) ppm.
The analytical data is in accordance with the literature.[7]

2.2 5-((3a*S*,4*S*,6a*R*)-2-oxohexahydro-1H-thieno[3,4-d]imidazol-4-yl)-N-((1-(4-(((*S*)-1,2,2-trimethyl-5-oxoimidazolidin-4-yl)methyl)phenyl)-1H-1,2,3-triazol-4-yl)methyl)pentanamide 1

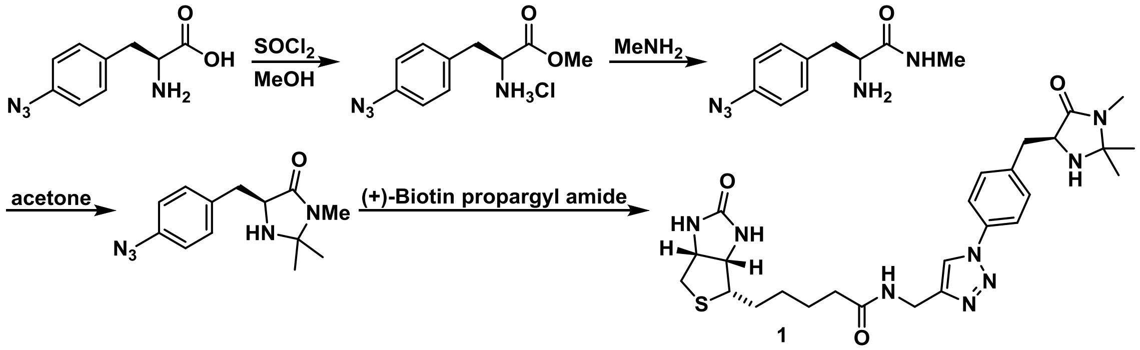

(*S*)-4-Azidophenylalanine methyl ester hydrochloride

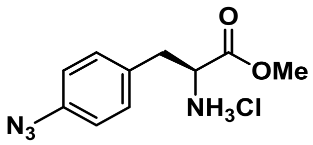

(*S*)-4-Azidophenylalanine (1.80 g, 8.72 mmol, 1.0 eq) was dissolved in MeOH (HPLC grade, 20 mL), cooled with an ice bath to 0 °C, and $SOCl_2$ (1.90 mL, 26.2 mmol, 3.0 eq) was added slowly under vigorous stirring. After complete addition, the reaction mixture was heated to 70 °C for 16 h. The mixture was cooled to room temperature and the volatiles were removed under reduced pressure at 60 °C. The residue was coevaporated with MeOH (3 × 50 mL), the title compound was obtained as off-white solid and used for further steps without additional purification.

(*S*)-4-Azidophenylalanine methyl amide

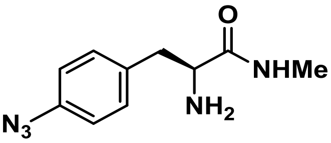

To (*S*)-4-azidophenylalanine methyl ester hydrochloride (425 mg, 1.65 mmol, 1.0 eq) in EtOH (10 mL) was added methylamine (33 *wt*% in EtOH, 2.4 mL, 20 mmol, 12 eq) and the resulting solution was stirred at room temperature until full conversion was detected (LCMS). The volatiles were removed under reduced pressure and the residue was taken up in CH_2Cl_2 (20 mL). The organic phase was washed with saturated aqueous $NaHCO_3$ solution (50 mL), the phases were separated, and the aqueous phase was back-extracted with CH_2Cl_2 (2 × 20 mL). The combined organic phases were dried over Na_2SO_4, the solvent was removed under reduced pressure, and the amide was obtained as yellow oil (254 mg, 1.16 mmol, 70% yield).

^{1}H NMR (400 MHz, $CDCl_3$): δ = 7.20 (d, *J* = 7.8 Hz, 2H), 6.98 (d, *J* = 7.8 Hz, 2H), 3.58 (dd, *J* = 8.9, 3.9 Hz, 1H), 3.22 (dd, *J* = 13.8, 3.7 Hz, 1H), 2.81 (d, *J* = 4.9 Hz, 3H), 2.72 (dd, *J* = 13.8, 9.4 Hz, 1H), 1.31 (br s, 2H) ppm. **^{13}C NMR** (126 MHz, $CDCl_3$) : δ = 174.5, 138.8, 134.6, 130.8, 119.4, 56.4, 40.3, 26.0 ppm. **HRMS** (ESI-(+)): m/z: calculated for $C_{10}H_{14}N_5O$: 220.1198 $[M+H]^+$; found: 220.1179.

(*S*)-5-(4-Azidobenzyl)-2,2,3-trimethylimidazolidin-4-one

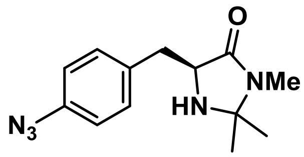

To a solution of (*S*)-4-azidophenylalanine methyl amide (307 mg, 1.4 mmol, 1.0 eq) and NEt_3 (150 µL, 1.1 mmol, 0.8 eq) in MeOH (5 mL) was added acetone (500 µL, 7.0 mmol, 5.0 eq) and the mixture was stirred until full conversion was detected (LCMS). The volatiles were removed under reduced pressure and the imidazolidinone was obtained as yellow oil (360 mg, 1.4 mmol, quantitative yield).

^{1}H NMR (400 MHz, $CDCl_3$): δ = 7.23 (d, *J* = 7.9 Hz, 2H), 6.96 (d, *J* = 7.9 Hz, 2H), 3.78–3.75 (m, 1H), 3.11 (dd, *J* = 14.3, 4.3 Hz, 1H), 2.98 (dd, *J* = 14.2, 6.9 Hz, 1H), 2.76 (s, 3H), 1.27 (s, 3H), 1.19 (s, 3H) ppm. **^{13}C NMR** (100 MHz, $CDCl_3$): δ = 173.3, 138.7, 134.1, 131.1, 119.3, 75.7, 59.4, 36.8, 27.5, 25.4 ppm. **HRMS** (EI): m/z: calculated for $C_{13}H_{17}N_5O$: 259.1433 $[M]^+$; found: 259.1432.

5-((3a*S*,4*S*,6a*R*)-2-oxohexahydro-1H-thieno[3,4-d]imidazol-4-yl)-*N*-((1-(4-(((*S*)-1,2,2-trimethyl-5-oxoimidazolidin-4-yl)methyl)phenyl)-1H-1,2,3-triazol-4-yl)methyl)pentanamide 1

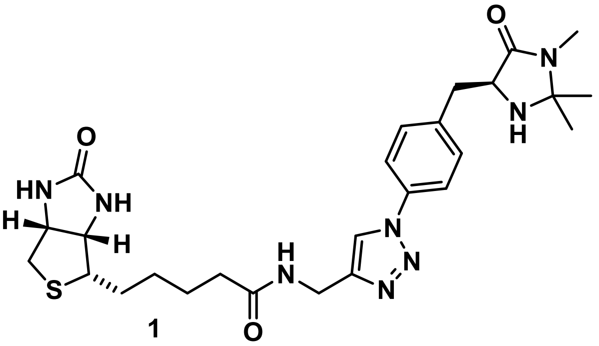

1

(*S*)-5-(4-Azidobenzyl)-2,2,3-trimethylimidazolidin-4-one (30 mg, 0.12 mmol, 1.0 eq) and (+)-biotin propargyl amide (33 mg, 0.12 mmol, 1.0 eq) were suspended in a H_2O:*tert*-butanol mixture (1:1, *V*/*V*, 2 mL) and (+)-sodium L-ascorbate (1 M in H_2O, 180 µL, 0.18 mmol, 1.5 eq) and copper sulfate pentahydrate (1 M in H_2O, 120 µL, 0.12 mmol, 1.0 eq) were added. The suspension was stirred at room temperature until full conversion of the azide was observed (TLC or LCMS). The solvent was removed under reduced pressure at 60 °C. The residue was dissolved in MeOH, concentrated on silica gel, and purified by column chromatography on silica gel (CH_2Cl_2:MeOH 95:5 → 90:10 → 80:20). Compound **1** was obtained as off-white solid (33 mg, 0.06 mmol, 50% yield).

^{1}H NMR (400 MHz, $CDCl_3$): δ = 8.04 (s, 1H), 7.79 (t, *J* = 5.6 Hz, 1H), 7.64 (d, *J* = 8.5 Hz, 2H), 7.40 (d, *J* = 8.5 Hz, 2H), 6.98 (s, 1H), 6.33 (s, 1H), 4.63 (dd, *J* = 15.0, 6.2 Hz, 1H), 4.56–4.51 (m, 1H), 4.44 (dd, *J* = 15.0, 5.7 Hz, 1H), 4.32 (dd, *J* = 7.9, 4.8 Hz, 1H), 3.83 (dd, *J* = 6.9, 4.4 Hz, 1H), 3.21 (dd, *J* = 14.2, 4.3 Hz, 1H), 3.11 (m_c, 1H), 3.01 (dd, *J* = 14.2, 7.2 Hz, 1H), 2.91 (dd, *J* = 12.8, 4.9 Hz, 1H), 2.77 (s, 3H), 2.76 (d, *J* = 10.5, 1H), 2.24 (m_c, 2H), 1.82–1.62 (m, 6H), 1.29 (s, 3H), 1.22 (s, 3H) ppm. **^{13}C NMR** (100 MHz, $CDCl_3$): δ = 173.7, 173.1, 164.8, 146.0, 138.7, 135.7, 131.0, 121.0, 120.7, 75.8, 61.7, 60.4, 59.4, 55.9, 40.8, 37.3, 35.9, 34.4, 28.2, 28.1, 27.7, 25.5, 25.4 ppm. **HRMS** (ESI-(+)): m/z: calculated for $C_{26}H_{37}N_8O_3S$: 541.2709 $[M+H]^+$; found: 541.2714.

2.3 *N*-((1-(4-(((2*S*,4*S*)-2-(*tert*-butyl)-1-methyl-5-oxoimidazolidin-4-yl)methyl)phenyl)-1H-1,2,3-triazol-4-yl)methyl)-5-((3a*S*,4*S*,6a*R*)-2-oxohexahydro-1H-thieno[3,4-d]imidazol-4-yl)pentanamide 2

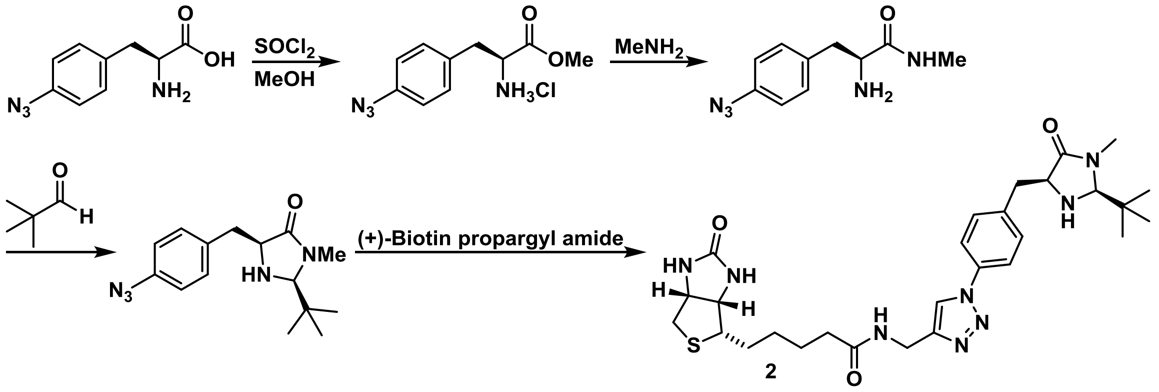

(2*S*,5*S*)-5-(4-azidobenzyl)-2-(*tert*-butyl)-3-methylimidazolidin-4-one

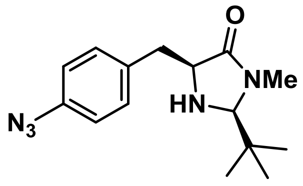

To a solution of (*S*)-4-azidophenylalanine methyl amide (219 mg, 1.0 mmol, 1.0 eq) and pivaldehyde (220 µL, 2.0 mmol, 2.0 eq) in $CHCl_3$ (10 mL) was added $Yb(OTf)_3$ (6 mg, 0.01 mmol, 0.01 eq) and the mixture was heated to reflux for 8 h. The volatiles were removed under reduced pressure and the oily residue was purified by column chromatography on silica gel (*n*-hexane:EtOAc 85:15 → 75:25 → 66:34). Both stereoisomers were obtained as yellow oils (*cis*: 16 mg, 0.06 mmol, 6% yield; *trans*: 88 mg, 0.31 mmol, 31% yield).

cis:
^{1}H NMR (300 MHz, $CDCl_3$): δ = 7.24 (d, *J* = 8.4 Hz, 2H), 6.95 (d, *J* = 8.4 Hz, 2H), 4.07 (d, *J* = 1.0 Hz, 1H), 3.69 (dd, *J* = 7.3, 3.7 Hz, 1H), 3.11 (dd, *J* = 13.8, 3.9 Hz, 1H), 2.92–2.85 (m, 1H), 2.91 (s, 3H), 1.78 (br s, 1H), 0.84 (s, 9H) ppm. **^{13}C NMR** (75 MHz, $CDCl_3$): δ = 175.1, 138.5, 134.8, 131.1, 119.2, 82.5, 59.5, 37.9, 35.2, 30.8, 25.4 ppm. **HRMS** (APCI-(+)): m/z: calculated for $C_{15}H_{22}N_5O$: 288.1824 $[M+H]^+$; found: 288.1814.

trans:
^{1}H NMR (400 MHz, $CDCl_3$): δ = 7.22 (d, *J* = 8.1 Hz, 2H), 6.96 (d, *J* = 8.1 Hz, 2H), 3.87–3.79 (m, 1H), 3.82 (s, 1H), 3.06 (dd, *J* = 14.1, 4.0 Hz, 1H), 2.91–2.84 (m, 1H), 2.88 (s, 3H), 0.91 (s, 9H) ppm. **HRMS** (APCI-(+)): m/z: calculated for $C_{15}H_{22}N_5O$: 288.1824 $[M+H]^+$; found: 288.1814.
The analytical data is in accordance with the literature.[8]

N-((1-(4-(((2S,4S)-2-(tert-butyl)-1-methyl-5-oxoimidazolidin-4-yl)methyl)phenyl)-1H-1,2,3-triazol-4-yl)methyl)-5-((3aS,4S,6aR)-2-oxohexahydro-1H-thieno[3,4-d]imidazol-4-yl)pentanamide 2

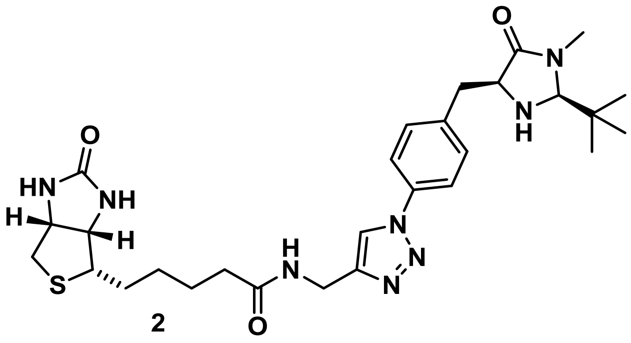

2

(2*S*,5*S*)-5-(4-azidobenzyl)-2-(*tert*-butyl)-3-methylimidazolidin-4-one (15 mg, 0.05 mmol, 1.0 eq) and (+)-biotin propargyl amide (16 mg, 0.06 mmol, 1.2 eq) were suspended in a H_2O:*tert*-butanol mixture (1:1, *V*/*V*, 2 mL) and (+)-sodium L-ascorbate (1.0 M in H_2O, 75 µL, 0.08 mmol, 1.5 eq) and copper sulfate pentahydrate (1.0 M in H_2O, 50 µL, 0.05 mmol, 1.0 eq) were added. The suspension was stirred at room temperature until full conversion of the azide was observed (TLC and LCMS). The solvent was removed under reduced pressure at 60 °C. The residue was dissolved in MeOH, concentrated on silica gel, and purified by column chromatography on silica gel (CH_2Cl_2:MeOH 95:5 → 90:10 → 80:20). Compound **2** was obtained as yellowish oil (14 mg, 0.03 mmol, 49% yield).

^{1}H NMR (400 MHz, $CDCl_3$): δ = 8.08 (s, 1H), 7.75 (br s, 1H), 7.62 (d, *J* = 8.0 Hz, 2H), 7.38 (d, *J* = 8.0 Hz, 2H), 6.52 (s, 1H), 6.04 (s, 1H), 4.61–4.40 (m, 3H), 4.31–4.21 (m, 1H), 3.81–3.71 (m, 1H), 3.17 (dd, *J* = 13.7, 2.9 Hz, 1H), 3.12–3.01 (m, 1H), 2.98–2.79 (m, 2H), 2.91 (s, 3H), 2.68 (d, *J* = 12.5 Hz, 1H), 2.31–2.13 (m, 3H), 1.73–1.30 (m, 6H), 0.85 (s, 9H) ppm. **^{13}C NMR** (126 MHz, $CDCl_3$): δ =174.7, 173.7, 164.6, 145.9, 139.5, 135.6, 131.0, 120.7, 82.5, 61.8, 60.5, 59.4, 55.7, 40.8, 38.6, 35.8, 35.4, 34.5, 30.8, 29.8, 28.1, 25.4 ppm. **HRMS** (ESI-(+)): m/z: calculated for $C_{28}H_{41}N_8O_3S$: 569.3022 $[M+H]^+$; found: 569.3021.

2.4 5-((3a*S*,4*S*,6a*R*)-2-oxohexahydro-1H-thieno[3,4-d]imidazol-4-yl)-*N*-((1-(2-((*S*)-1,2,2-trimethyl-5-oxoimidazolidin-4-yl)ethyl)-1H-1,2,3-triazol-4-yl)methyl)pen-tanamide 3

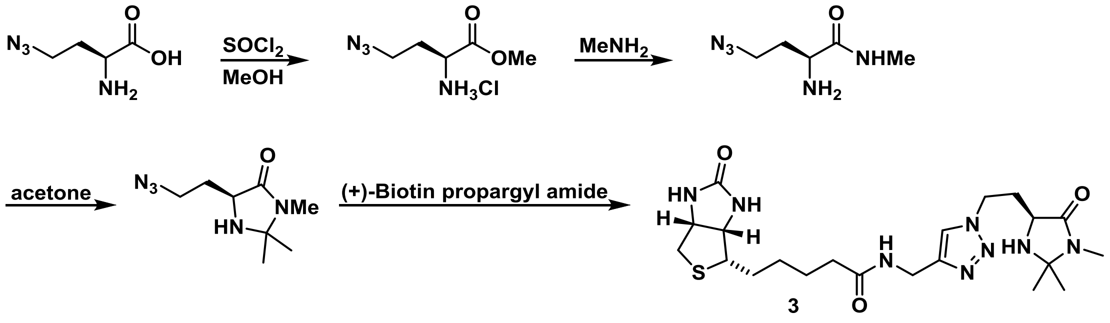

(*S*)-2-Amino-4-azidobutanoic acid methyl ester hydrochloride

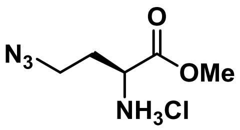

(*S*)-2-Amino-4-azidobutanoic acid hydrochloride (50 mg, 0.28 mmol, 1.0 eq) was dissolved in MeOH (HPLC grade, 10 mL), cooled with an ice bath to 0 °C, and $SOCl_2$ (0.3 mL, 4.1 mmol, 15.0 eq) was added slowly under vigorous stirring. After complete addition, the reaction mixture was heated to 60 °C for 16 h. The mixture was cooled to room temperature and the volatiles were removed under reduced pressure at 60 °C. The residue was coevaporated with MeOH (3 × 20 mL), the title compound was obtained as off-white solid and used for further steps without additional purification.

(*S*)-*N*-Methyl-2-amino-4-azidobutanamide

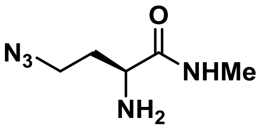

To (*S*)-2-amino-4-azidobutanoic acid methyl ester hydrochloride (55 mg, 0.28 mmol, 1.0 eq) was added methylamine (33 *wt*% in EtOH, mL, eq) and the resulting solution was stirred at room temperature until full conversion was detected (LCMS). The volatiles were removed under reduced pressure and the residue was taken up in CH_2Cl_2 (20 mL). The organic phase was washed with saturated aqueous $NaHCO_3$ solution (50 mL), the phases were separated, and the aqueous phase was back-extracted with CH_2Cl_2 (2 × 20 mL). The combined organic phases were dried over Na_2SO_4, the solvent was removed under reduced pressure, and the amide was obtained as yellow oil (30 mg, 0.20 mmol, 68% yield).

^{1}H NMR (400 MHz, $CDCl_3$): δ = 7.24 (br s, 1H), 3.52–3.44 (m, 3 H), 2.83 (d, *J* = 5 Hz, 3H), 2.17–2.07 (m, 1H), 1.76 (td, *J* = 14.0, 6.8 Hz, 1H), 1.58 (s, 2H) ppm. **^{13}C NMR** (100 MHz, $CDCl_3$): δ =174.8, 53.5, 49.1, 34.3, 26.0 ppm. **LCMS**: 157.9 [14%, $(M+H)^+$], 170.9 [100%, $(M+Na)^+$].

(*S*)-5-(2-Azidoethyl)-2,2,3-trimethylimidazolidin-4-one

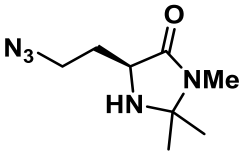

To a solution of (*S*)-*N*-methyl-2-amino-4-azidobutanamide (44 mg, 0.28 mmol, 1.0 eq) and NEt_3 (22 mg, 31 µL, 0.22 mmol, 0.8 eq) in MeOH (10 mL) was added acetone (100 µL, 1.4 mmol, 5.0 eq) and the mixture was stirred until full conversion was detected (LCMS). The volatiles were removed under reduced pressure and the oily residue was purified by column chromatography on silica gel (CH_2Cl_2:MeOH 95:5). The imidazolidinone was obtained as yellow oil (48 mg, 0.24 mmol, 87% yield).

^{1}H NMR (400 MHz, $CDCl_3$): δ = 3.61 (dd, *J* = 8.3, 4.5 Hz, 1H), 3.54–3.46 (m, 2 H), 2.78 (s, 3H), 2.15 (dtd, *J* = 11.7, 7.1, 4.7 Hz, 1H), 1.91 (br s, 2H), 1.76–1.68 (m, 1H), 1.38 (s, 3H), 1.32 (s, 3H) ppm. **^{13}C NMR** (100 MHz, $CDCl_3$): δ =173.5, 75.9, 56.1, 48.7, 31.8, 27.9, 25.33, 25.31 ppm. **HRMS** (ESI-(+)): m/z: calculated for $C_8H_{16}N_5O$: 198.1355 $[M+H]^+$; found: 198.1329.

5-((3a*S*,4*S*,6a*R*)-2-oxohexahydro-1H-thieno[3,4-d]imidazol-4-yl)-*N*-((1-(2-((*S*)-1,2,2-trimethyl-5-oxoimidazolidin-4-yl)ethyl)-1H-1,2,3-triazol-4-yl)methyl)pentanamide 3

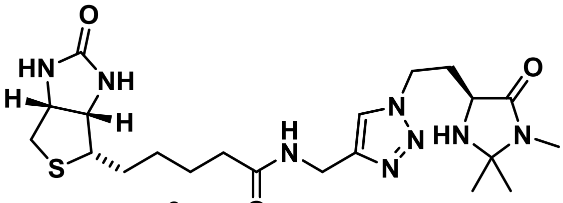

3

(*S*)-5-(2-Azidoethyl)-2,2,3-trimethylimidazolidin-4-one (17 mg, 0.09 mmol, 1.0 eq) and (+)-biotin propargyl amide (25 mg, 0.09 mmol, 1.0 eq) were suspended in a H_2O:*tert*-butanol mixture (1:1, *V*/*V*, 2 mL) and (+)-sodium L-ascorbate (1.0 M in H_2O, 135 µL, 0.14 mmol, 1.5 eq) and copper sulfate pentahydrate (1.0 M in H_2O, 90 µL, 0.09 mmol, 1.0 eq) were added. The suspension was stirred at room temperature until full conversion of the azide was observed (LCMS). The solvent was removed under reduced pressure. The residue was dissolved in MeOH, concentrated on silica gel, and purified by column chromatography on silica gel (CH_2Cl_2:MeOH 95:5 → 90:10 → 80:20). Compound **3** was obtained as off-white solid (34 mg, 0.07 mmol, 79% yield).

^{1}H NMR (400 MHz, $CDCl_3$): δ = 7.78 (t, *J* = 5.3 Hz, 1H), 7.67 (s, 1 H), 7.07 (s, 1H), 6.40 (s, 1H), 4.61–4.45 (m, 4H), 4.43–4.28 (m, 2H), 3.49–3.45 (m, 1H), 3.12 (dd, *J* = 11.6, 7.1 Hz, 1H), 2.90 (dd, *J* = 12.8, 4.8 Hz, 1H), 2.77–2.74 (m, 1H), 2.77 (s, 3H), 2.36 (dt, *J* = 12.9, 7.5 Hz, 1H), 2.24–2.18 (m, 2H), 2.07–1.97 (m, 2H), 1.77–1.58 (m, 4H), 1.48–1.22 (m, 2H), 1.38 (s, 3H), 1.33 (s, 3H) ppm. **^{13}C NMR** (100 MHz, $CDCl_3$): δ =173.5, 172.9, 164.7, 145.3, 123.1, 76.1, 61.8, 60.4, 55.9, 55.3, 47.2, 40.8, 35.8, 34.5, 33.7, 28.22, 28.18, 28.0, 25.7, 25.5, 25.3 ppm. **HRMS** (ESI-(+)): m/z: calculated for $C_{21}H_{35}N_8O_3S$ 479.2553 $[M+H]^+$; found: 479.2567.

2.5 *N*-((1-(2-((4*S*)-2-(*tert*-butyl)-1-methyl-5-oxoimidazolidin-4-yl)ethyl)-1H-1,2,3-triaz-ol-4-yl)methyl)-5-((3a*S*,4*S*,6a*R*)-2-oxohexahydro-1H-thieno[3,4-d]imidazol-4-yl)pentanamide 4

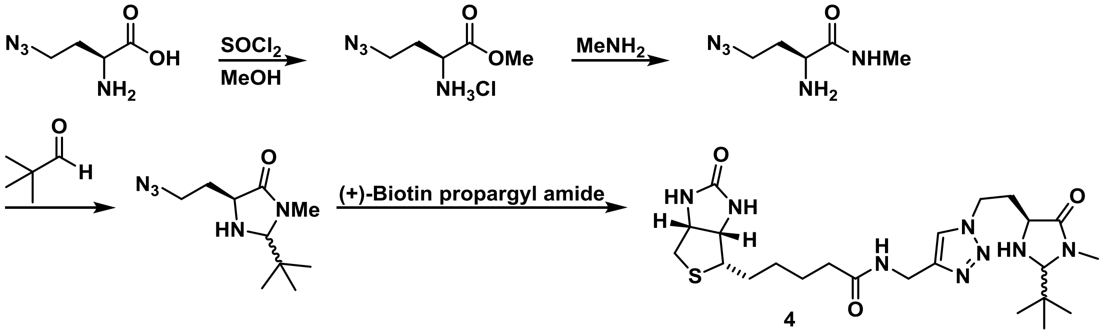

(5*S*)-5-(2-azidoethyl)-2-(*tert*-butyl)-3-methylimidazolidin-4-one

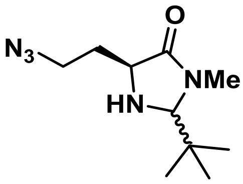

To a solution of (*S*)-*N*-methyl-2-amino-4-azidobutanamide (30 mg, 0.19 mmol, 1.0 eq) and pivaldehyde (40 µL, 0.38 mmol, 2.0 eq) in $CHCl_3$ (5 mL) was added $Yb(OTf)_3$ (1 mg, 2 µmol, 0.01 eq) and the mixture was heated to reflux for 16 h until full conversion of the starting material was observed (TLC). The volatiles were removed under reduced pressure and the oily residue was purified by column chromatography on silica gel (*n*-hexane:EtOAc 75:25). The imidazolidinone was obtained as an inseparable mixture of both stereoisomers (~50:50) as yellow oil (40 mg, 0.18 mmol, 95% yield).

^{1}H NMR (400 MHz, $CDCl_3$): δ = 4.14 (s, 1H), 4.06 (s, 1H), 3.73–3.66 (m, 1H), 3.63 (dd, *J* = 8.2, 4.2 Hz, 1H), 3.52–3.44 (m, 4H), 2.94 (s, 3H), 2.92 (s, 3H), 2.17–1.98 (m, 4H), 1.80–1.64 (m, 2H), 0.96 (s, 9H), 0.95 (s, 9H) ppm. **^{13}C NMR** (100 MHz, $CDCl_3$): δ =175.4, 175.0, 83.7, 82.6, 56.2, 55.7, 48.59, 48.58, 37.8, 35.5, 32.52, 32.49, 31.5, 30.7, 26.2, 25.7, 25.4 ppm. **HRMS** (ESI-(+)): m/z: calculated for $C_{10}H_{20}N_5O$ 226.1668 $[M+H]^+$; found: 226.1666.

N-((1-(4-(((2S,4S)-2-(tert-butyl)-1-methyl-5-oxoimidazolidin-4-yl)methyl)phenyl)-1H-1,2,3-triazol-4-yl)methyl)-5-((3aS,4S,6aR)-2-oxohexahydro-1H-thieno[3,4-d]imidazol-4-yl)pentanamide 4

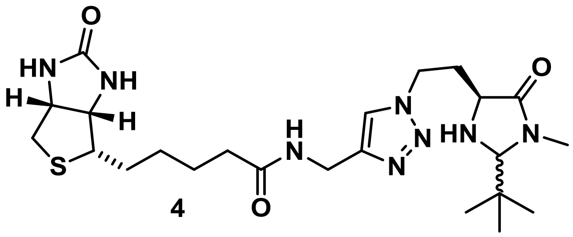

(5*S*)-5-(2-azidoethyl)-2-(*tert*-butyl)-3-methylimidazolidin-4-one (7 mg, 0.031 mmol, 1.0 eq) and (+)-biotin propargyl amide (9 mg, 0.032 mmol, 1.0 eq) were suspended in a H_2O:*tert*-butanol mixture (1:1, *V*/*V*, 2 mL) and (+)-sodium L-ascorbate (1.0 M in H_2O, 50 µL, 0.05 mmol, 1.6 eq) and copper sulfate pentahydrate (1.0 M in H_2O, 30 µL, 0.030 mmol, 1.0 eq) were added. The suspension was stirred at room temperature until full conversion of the azide was observed (TLC or LCMS). The solvent was removed under reduced pressure. The residue was dissolved in MeOH, concentrated on silica gel, and purified by column chromatography on silica gel (CH_2Cl_2:MeOH 95:5 → 90:10 → 80:20). Compound **4** was obtained as yellowish film (4 mg, 0.08 mmol, 25% yield).

^{1}H NMR (500 MHz, methanol-d_4): δ = 7.81 + 7.79 (s, 1H), 4.48–4.44 (m, 1H), 4.40 (dd, *J* = 7.7, 4.8 Hz, 1H), 4.33 (s, 2H), 4.20 (dd, *J* = 7.8, 4.4 Hz, 1H), 4.07 (d, *J* = 1.9 Hz, 1H), 2.87–2.80 (m, 4H), 2.61 (d, *J* = 12.5 Hz, 1H), 2.14 (t, *J* = 7.3 Hz, 3H), 2.07–2.02 (m, 1H), 1.95–1.86 (m, 1H), 1.65–1.42 (m, 6H), 1.37–1.29 (m, 2H), 0.89 + 0.87 (s, 9H) ppm. **^{13}C NMR** (126 MHz, methanol-d_4): δ = 177.2, 175.9, 166.1, 146.4, 124.5, 85.2, 63.3, 61.6, 57.1, 57.0, 48.0, 41.1, 38.7, 36.6, 35.6, 34.7, 33.1, 31.8, 30.78, 30.75, 29.7, 29.4, 26.7, 25.9 ppm. **HRMS** (ESI-(+)): m/z: calculated for $C_{23}H_{39}N_8O_3S$: 507.2866 $[M+H]^+$; found: 507.2881.

2.6 (2*S*,4*S*)-2-carboxy-4-(4-((5-((3a*S*,4*S*,6a*R*)-2-oxohexahydro-1H-thieno[3,4-d]imidazol-4-yl)pentanamido)methyl)-1H-1,2,3-triazol-1-yl)pyrrolidin-1-ium trifluoroacetate 5

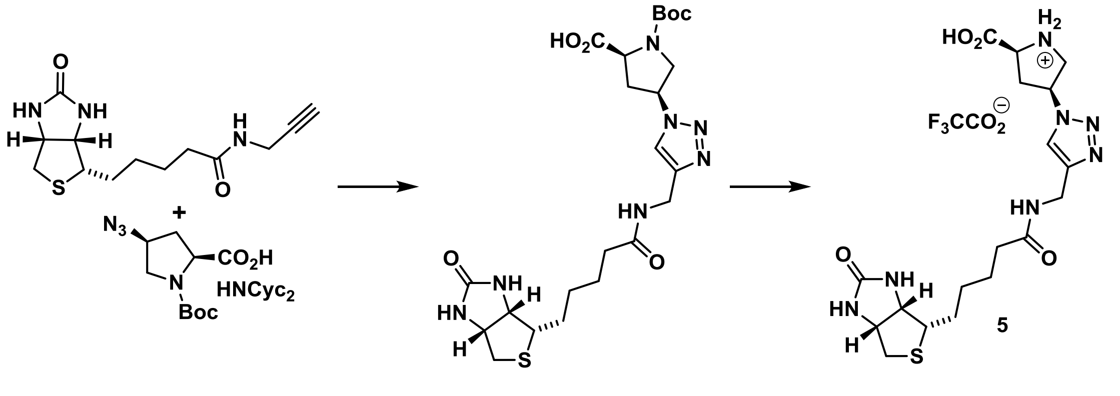

(2*S*,4*S*)-1-(*tert*-butoxycarbonyl)-4-(4-((5-((3a*S*,4*S*,6a*R*)-2-oxohexahydro-1H-thieno[3,4-d]imidazol-4-yl)pentanamido)methyl)-1H-1,2,3-triazol-1-yl)pyrrolidine-2-carboxylic acid

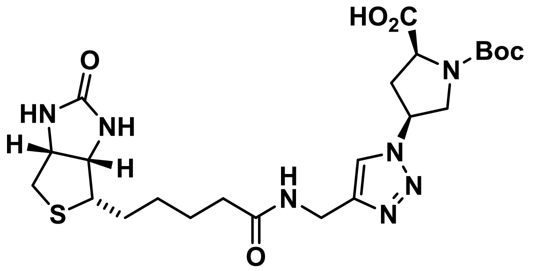

(+)-Biotin propargyl amide (140 mg, 0.50 mmol, 1.0 eq) and *N*-Boc-*cis*-4-azido-L-proline dicyclohexylammonium salt (215 mg, 0.50 mmol, 1.0 eq) were suspended in a H_2O:*tert*-butanol mixture (1:1, 2.0 mL). (+)-Sodium L-ascorbate (1.0 M in H_2O, 750 µL, 0.75 mmol, 1.5 eq) and copper sulfate pentahydrate (1.0 M in H_2O, 500 µL, 0.50 mmol, 1.0 eq) were added, the suspension was stirred at room temperature, and the progress was monitored by LCMS. After full conversion the pH of the mixture was adjusted to approximately 4.0 by careful addition of Amberlite IR-120 ion exchange resin. The mixture was filtered through celite, washed with MeOH (50 mL), and the filtrate was concentrated on silica gel. Column chromatography on silica gel (CH_2Cl:MeOH 95:5 → 90:10 → 80:20) afforded the triazole as off-white solid (70 mg, 0.13 mmol, 26%).

^{1}H NMR (400 MHz, MeOH-d_4): δ = 8.05 (s, 1H), 5.20 (p, J = 8.0 Hz, 1H), 4.50 (dd, J = 7.6, 5.0 Hz, 1H), 4.44 (s, *2*H), 4.30 (dd, J = 7.8, 4.5 Hz, 1H), 4.23 (m_c, 1H), 4.18–4.11 (m, 1H), 3.76 (m_c, 1H), 3.22–3.16 (m_c, 1H), 2.94 (dd, J = 12.8, 5.7 Hz, 2H), 2.71 (d, J = 12.7, 1H), 2.40 (m_c, 1H), 2.25 (t, J = 7.3 Hz, 2H), 1.79–1.54 (m, 4H), 1.52–1.39 (m, 2H), 1.45 (s, 9H) ppm. **^{13}C NMR** (100 MHz, MeOH-d_4): δ = 179.1, 175.9, 166.1, 156.1, 146.3, 123.0, 81.5, 63.3, 61.6, 58.7, 57.0, 54.8, 41.0, 37.9, 36.6, 35.6, 29.7, 29.4, 28.7, 26.7 ppm. **HRMS** (ESI-(+)): m/z: calculated for $C_{23}H_{36}N_7O_6S$: 538.2448 $[M+H]^+$; found, 538.2448.

(2*S*,4*S*)-2-carboxy-4-(4-((5-((3a*S*,4*S*,6a*R*)-2-oxohexahydro-1H-thieno[3,4-d]imidazol-4-yl)pentanamido)methyl)-1H-1,2,3-triazol-1-yl)pyrrolidin-1-ium trifluoroacetate 5

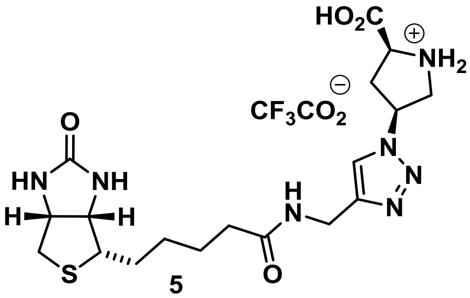

5

(2*S*,4*S*)-1-(*tert*-butoxycarbonyl)-4-(4-((5-((3a*S*,4*S*,6a*R*)-2-oxohexahydro-1H-thieno[3,4-d]imidazol-4-yl)pentanamido)methyl)-1H-1,2,3-triazol-1-yl)pyrrolidine-2-carboxylic acid (23 mg, 0.043 mmol, 1.0 eq) was suspended in CH_2Cl_2 (2 mL) and cooled to 0 °C with an ice bath. Trifluoroacetic acid (TFA, 1.0 mL, 13.0 mmol, 300 eq) was added under stirring. After addition the ice bath was removed and the reaction progress was monitored (LCMS). Upon completion the volatiles were removed under reduced pressure to afford compound **5** as brown oil (17 mg, 0.039 mmol, 91% yield), which was found pure enough for further use.

^{1}H NMR (400 MHz, MeOH-d_4): δ = δ = 7.98 (s, 1H), 5.54–5.44 (m, 1H), 4.70 (dd, *J* = 9.4, 6.9 Hz, 1H), 4.55–4.48 (m, 1H), 4.43 (s, *2*H), 4.30 (dd, *J* = 7.4, 4.4 Hz, 1H), 3.91 (d, *J* = 5.1 Hz, 2H), 3.24–3.18 (m, 1H), 3.13–3.05 (m, 1H), 2.93 (dd, *J* = 12.7, 4.8 Hz, 1H), 2.88–2.78 (m, 1H), 2.70 (d, *J* = 12.7 Hz, 1H), 2.24 (t, *J* = 7.1 Hz, 2H), 1.79–1.60 (m, 4H), 1.51–1.36 (m, 2H) ppm. **^{13}C NMR** (100 MHz, MeOH-d_4): δ = 176.1, 170.3, 166.1, 147.0, 124.3, 63.3, 61.6, 59.5, 57.0, 51.8, 41.0, 36.5, 35.5, 29.5, 29.4, 26.7 ppm. **HRMS** (ESI-(+)): m/z: calculated for $C_{18}H_{28}N_7O_4S$: 438.1923 $[M+H]^+$; found, 438.1911.

2.7 (2*S*,4*S*)-2-carboxy-4-(5-((3a*S*,4*S*,6a*R*)-2-oxohexahydro-1H-thieno[3,4-d]imidazol-4-yl)pentanamido)pyrrolidin-1-ium formate 6

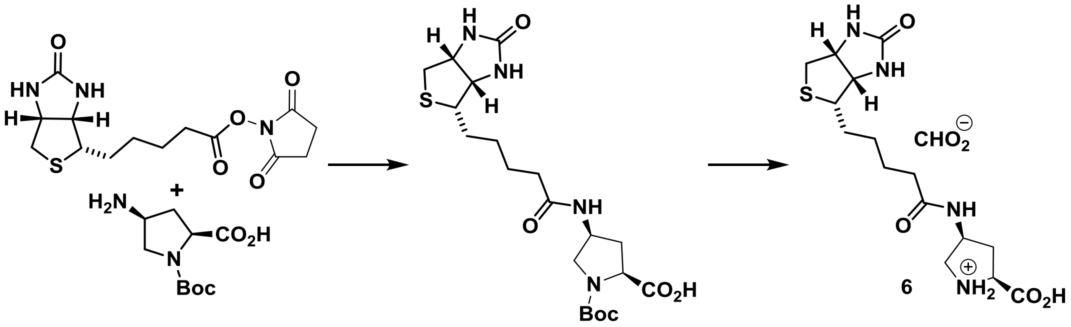

(
+)-Biotin NHS ester (67 mg, 0.20 mmol, 1.0 eq) and (2*S*,4*S*)-4-amino-1-Boc-proline (46 mg, 0.20 mmol, 1.0 eq) were dissolved in dry DMF. Di-*iso*-propylethylamine (DIPEA, 73 µL, 0.40 mmol, 2.0 eq) was added and the reaction was allowed to proceed under stirring overnight at room temperature. The solvent was removed under reduced pressure at 80 °C. The residue was dissolved in a mixture of TFA:H_2O (95:5, 5.0 mL) and stirred at room temperature for 1 h. Excess TFA was removed under reduced pressure, the residue was dissolved in a minimal amount of H_2O and lyophilized. The crude product was purified by preparative HPLC (*Supelcosil* C_{18} column, 25 cm x 21.2 mm, 12 µm; gradient H_2O:MeCN 99:1 to 50:50 over 30 min, 0.1% HCO_2H, 10 mL·min^{-1}, 20 °C, λ = 210 nm) and compound **6** was obtained as white solid (51 mg, 0.13 mmol, 63% yield).

^{1}H NMR (400 MHz, D_2O): δ = 4.59 (dd, *J* = 7.8, 5.0 Hz, 1H), 4.49–4.44 (m, 1H), 4.41 (dd, *J* = 7.8, 4.4 Hz, 1H), 4.20 (t, *J* = 8.3 Hz, 1H), 3.62 (dd, *J* = 12.3, 7.1 Hz, 1H), 3.38–3.30 (m, 2H), 2.98 (dd, *J* = 13.1, 4.9 Hz, 1H), 2.77 (d, *J* = 13.1 Hz, 1H), 2.74–2.64 (m, 1H), 2.24 (t, *J* = 7.3 Hz, 2H), 2.09 (dt, *J* = 14.0, 7.1 Hz, 1H), 1.77–1.50 (m, 4H), 1.46–1.31 (m, 2H) ppm. **^{13}C NMR** (100 MHz, D_2O): δ = 177.1, 173.5, 165.4, 62.0, 60.2, 60.1, 55.2, 49.3, 48.5, 39.6, 35.2, 34.0, 27.8, 27.6, 24.8 ppm. **HRMS** (ESI-(+)): m/z: calculated for $C_{15}H_{25}N_4O_4S$: 357.1597 $[M+H]^+$; found, 357.1595.

2.8 (*R*)-3-(5-((3a*S*,4*S*,6a*R*)-2-oxohexahydro-1H-thieno[3,4-d]imidazol-4-yl)pentanamido)pyrrolidin-1-ium formate 7

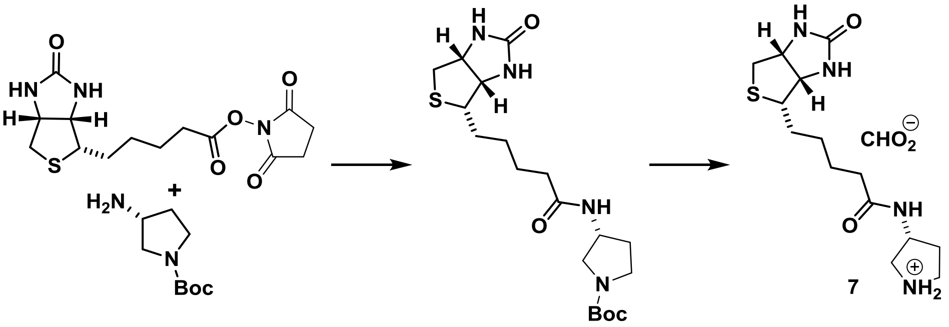

(+)-Biotin NHS ester (100 mg, 0.29 mmol, 1.0 eq) and (3*R*)-3-amino-1-Boc-pyrrolidine (64 mg, 0.29 mmol, 1.0 eq) were dissolved in dry DMF. Di-*iso*-propylethylamine (DIPEA, 106 µL, 0.58 mmol, 2.0 eq) was added and the reaction was allowed to proceed under stirring overnight at room temperature. The solvent was removed under reduced pressure at 80 °C. The residue was dissolved in a mixture of TFA:H_2O (95:5, 5.0 mL) and stirred at room temperature for 1 h. Excess TFA was removed under reduced pressure, the residue was dissolved in a minimal amount of H_2O and lyophilized. The crude product was purified by preparative HPLC (*Supelcosil* C_{18} column, 25 cm x 21.2 mm, 12 µm; gradient H_2O:MeCN 99:1 to 50:50 over 30 min, 0.1% HCO_2H, 10 mL·min^{-1}, 20 °C, λ = 210 nm) and compound **7** was obtained as white solid (43 mg, 0.12 mmol, 42% yield).

^{1}H NMR (400 MHz, D_2O): δ = 4.57 (dd, *J* = 7.8, 4.9 Hz, 1H), 4.45–4.35 (m, 2H), 3.54 (dd, *J* = 12.5, 7.0 Hz, 1H), 3.46–3.35 (m, 2H), 3.30 (dt, *J* = 9.6, 4.1 Hz, 1H), 3.19 (dd, *J* = 12.5, 4.9 Hz, 1H), 2.95 (dd, *J* = 13.1, 4.9 Hz, 1H), 2.74 (d, *J* = 13.1 Hz, 1H), 2.38–2.27 (m, 1H), 2.23 (t, *J* = 7.2 Hz, 2H), 1.99 (td, *J* = 13.5, 6.8 Hz, 1H), 1.76–1.47 (m, 4H), 1.45–1.31 (m, 2H) ppm. **^{13}C NMR** (100 MHz, D_2O): δ = 177.1, 170.9, 165.3, 62.0, 60.2, 55.3, 49.6, 48.6, 44.3, 39.6, 35.1, 29.4, 27.8, 27.6, 24.9 ppm. **HRMS** (ESI-(+)): m/z: calculated for $C_{14}H_{25}N_4O_2S$: 313.1698 $[M+H]^+$; found, 313.1688.

2.9 (*S*)-3-(5-((3a*S*,4*S*,6a*R*)-2-oxohexahydro-1H-thieno[3,4-d]imidazol-4-yl)pentanamido)pyrrolidin-1-ium formate 8

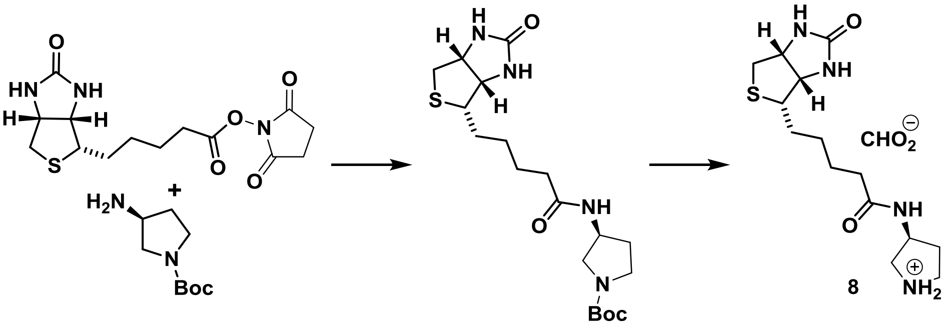

(+)-Biotin NHS ester (100 mg, 0.29 mmol, 1.0 eq) and (3*S*)-3-amino-1-Boc-pyrrolidine (64 mg, 0.29 mmol, 1.0 eq) were dissolved in dry DMF. Di-*iso*-propylethylamine (DIPEA, 106 µL, 0.58 mmol, 2.0 eq) was added and the reaction was allowed to proceed under stirring overnight at room temperature. The solvent was removed under reduced pressure at 80 °C. The residue was dissolved in a mixture of TFA:H_2O (95:5, 5.0 mL) and stirred at room temperature for 1 hr. Excess TFA was removed under reduced pressure, the residue was dissolved in a minimal amount of H_2O and lyophilized. The crude product was purified by preparative HPLC (*Supelcosil* C_{18} column, 25 cm x 21.2 mm, 12 µm; gradient H_2O:MeCN 99:1 to 50:50 over 30 min, 0.1% HCO_2H, 10 mL·min^{-1}, 20 °C, λ = 210) and compound **6** was obtained as white solid (63 mg, 0.18 mmol, 63% yield).

^{1}H NMR (400 MHz, D_2O): δ = 4.60–4.55 (m, 1H), 4.45–4.35 (m, 2H), 3.54 (dd, *J* = 12.4, 7.0 Hz, 1H), 3.47–3.34 (m, 2H), 3.33–3.26 (m, 1H), 3.20 (dd, *J* = 12.5, 4.7 Hz, 1H), 2.95 (dd, *J* = 13.0, 4.7 Hz, 1H), 2.74 (d, *J* = 13.1 Hz, 1H), 2.38–2.27 (m, 1H), 2.23 (t, *J* = 7.1 Hz, 2H), 2.05–1.94 (m, 1H), 1.75–1.47 (m, 4H), 1.45–1.27 (m, 2H) ppm. **^{13}C NMR** (100 MHz, D_2O): δ = 177.1, 170.9, 165.3, 62.0, 60.2, 55.3, 49.6, 48.6, 44.3, 39.6, 35.1, 29.4, 27.8, 27.6, 24.9 ppm. **HRMS** (ESI-(+)): m/z: calculated for $C_{14}H_{25}N_4O_2S$: 313.1698 [M+H]$^+$; found, 313.1696.

3 Experimental Details for the Activity Screening of Catalysts 1–7 in the Michael Addition of Nitromethane to Cinnamaldehyde

3.1 GCMS Based Screening for Catalyst Activity

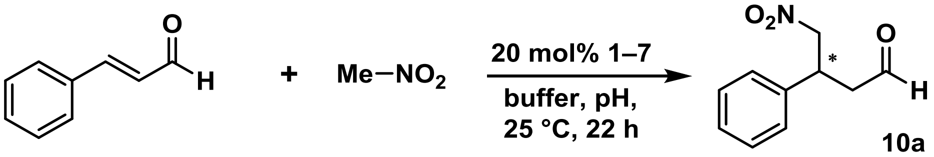

The respective catalyst (2.6 µmol, 0.20 eq) was added to an 1.5 mL Eppendorf tube. In case of an oily catalyst, a stock solution in MeOH (HPLC grade) was prepared. An aliquot of the stock solution was added to the Eppendorf tube and most of the MeOH was evaporated by shaking the tube at 400 rpm for at least 20 minutes at 50 °C. Deionized water (500 µL) or buffer (500 µL, 10 mM, pH 2.0-3.0: citrate buffer, pH 4.0-7.0: KP_i buffer, pH 8.0-9.0: Tris buffer) was added and the mixture was homogenized by shaking at 1400 rpm for 5 minutes at 25 °C. Nitromethane (3.5 µL, 65.3 µmol, 5.00 eq) and cinnamaldehyde (1.3 µL, 13.1 µmol, 1.00 eq) were added and the mixture was shaken at 1400 rpm at 25 °C for 22 hrs. CH_2Cl_2 (500 µL) was added and the biphasic mixture was shaken vigorously for 3 minutes. An aliquot (100 µL) of the organic phase was taken, diluted with CH_2Cl_2 (900 µL), and subjected to GCMS analysis.

4 Experimental Details for the Activity Screening of Catalysts 1–7 and Sav in the Michael Addition of Nitromethane to Cinnamaldehyde

4.1 GCMS Based Screening for Hybrid Catalyst Activity

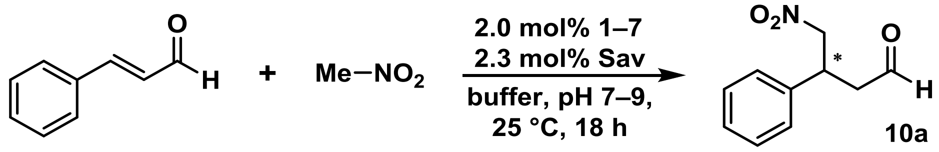

Pure Catalyst Runs

A stock solution of the respective biotinylated organocatalyst (2.59 mM in KP_i buffer, pH 7.0 & 8.0, or Tris buffer, pH 9.0, 10 mM) was prepared. 150 µL of the respective buffer as well as 24 µL of the organocatalyst stock solution (62 nmol, 2.0 mol%) were added into an 1.5 mL Eppendorf tube. The pH was adjusted carefully to 7.0, 8.0, or 9.0 by using 0.1 M NaOH and 0.1 M HCl stock solutions. The mixture was filled up to 250 µL with the respective buffer. Stock solutions of cinnamaldehyde (1.33 M in MeOH, HPLC grade) and nitromethane (6.61 M in MeOH, HPLC grade) were prepared. Subsequently, 2.5 µL of the nitromethane stock solution (16.5 µmol, 5.0 eq), and 2.5 µL of the aldehyde stock solution (3.3 µmol, 1.0 eq) were added to the catalyst solution. The reaction mixture was placed in an Eppendorf tube shaker (300 rpm, 25 °C) for 18 h. The tube was taken out of the shaker and the aqueous mixture was extracted once with CH_2Cl_2 (500 µL). An aliquot (100 µL) of the organic phase was taken, diluted with CH_2Cl_2 (900 µL), and subjected to GCMS analysis.

Organocatalytic Artificial Enzyme Runs

A stock solution of the respective biotinylated organocatalyst (2.59 mM in KP_i buffer, pH 7.0 & 8.0, or Tris buffer, pH 9.0, 10 mM) was prepared. 1.15 mg commercial **Sav** (77 nmol active sites, 2.3 mol%, contains 0.15 mg KP_i mixture, pH 6.5) were weighed into an 1.5 mL Eppendorf tube. 150 µL of the respective buffer as well as 24 µL of the organocatalyst stock solution (62 nmol, 2.0 mol%) were added. The mixture was vortexed for 15 s, spun down in an Eppendorf tube centrifuge (13,000 *g*), and the pH was adjusted carefully to 7.0, 8.0, or 9.0 by using 0.1 M NaOH and 0.1 M HCl stock solutions. The mixture was filled up to 250 µL with the respective buffer. Stock solutions of cinnamaldehyde (1.33 M in MeOH, HPLC grade) and nitromethane (6.61 M in MeOH, HPLC grade) were prepared. Subsequently, 2.5 µL of the nitromethane stock solution (16.5 µmol, 5.0 eq), and 2.5 µL of the aldehyde stock solution (3.3 µmol, 1.0 eq) were added to the hybrid catalyst solution. The reaction mixture was placed in an Eppendorf tube shaker (300 rpm, 25 °C) for 18 h. The tube was taken out of the shaker and the aqueous mixture was extracted once with CH_2Cl_2 (500 µL). An aliquot (100 µL) of the organic phase was taken, diluted with CH_2Cl_2 (900 µL), and subjected to GCMS analysis.

4.2 ^{1}H NMR Based Screening for Yield Determination

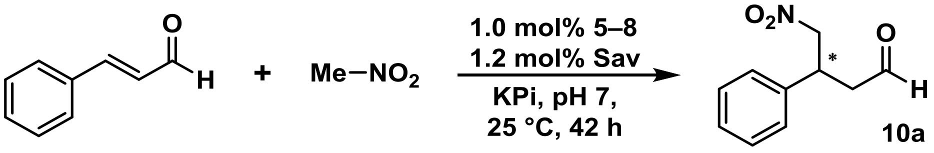

Catalytic Runs – KP_i Buffer

A stock solution of the respective biotinylated organocatalyst (2.59 mM in KP_i buffer, pH 7.0 10 mM) was prepared. In case of the hybrid catalyst runs 0.58 mg commercial **Sav** (38 nmol active sites, 1.2 mol%, contains 75 µg KP_i mixture, pH 6.5) were weighed into an 1.5 mL Eppendorf tube. 400 µL of buffer (KP_i buffer, pH 7.0, 10 mM) as well as 12 µL of the organocatalyst stock solution (31 nmol, 1.0 mol%) were added into an 1.5 mL Eppendorf tube, or to **Sav** in case of the hybrid catalyst runs. The mixture was vortexed for 15 s, spun down in an Eppendorf tube centrifuge (13,000 *g*), and the pH was adjusted carefully to 7.0 by using 0.1 M NaOH and 0.1 M HCl stock solutions. The mixture was filled up to 500 µL with buffer (KP_i buffer, pH 7.0, 10 mM). Stock solutions of cinnamaldehyde (1.33 M in MeOH, HPLC grade), nitrobenzene (1.33 M in MeOH, HPLC grade, use of *ortho* protons as internal standard), and nitromethane (6.61 M in MeOH, HPLC grade) were prepared. Subsequently, 2.5 µL of the nitromethane stock solution (16.5 µmol, 5.0 eq), 2.5 µL of the nitrobenzene stock solution (3.3 µmol, 1.0 eq), and 2.5 µL of the aldehyde stock solution (3.3 µmol, 1.0 eq) were added to the catalyst solution. The Eppendorf tube was placed in a Falcon tube, which was placed inside an incubator shaker (50 rpm, 25 °C) for 42 h. The tube was taken out of the incubator and the aqueous mixture was extracted once with $CDCl_3$ (700 µL). The organic phase was transferred into an NMR tube and directly subjected to ^{1}H NMR analysis. Initial attempts to estimate product conversion were made by measuring the ratio of internal standard nitrobenzene with that of starting material, but the integrals were found to be skewed after organic solvent extraction. Instead, product conversion was estimated by measuring the integral ratio between the substrate double bond and product CH_2 group (see Table 9.1)

Catalytic Runs – Organic Solvents

A stock solution of the respective biotinylated organocatalyst (2.59 mM in KP_i buffer, pH 7.0 10 mM) was prepared. 0.58 mg commercial **Sav** (38 nmol active sites, 1.2 mol%, contains 75 µg KP_i mixture, pH 6.5) were weighed into an 1.5 mL Eppendorf tube. 188 µL of buffer (KP_i buffer, pH 7.0, 10 mM) as well as 12 µL of the organocatalyst stock solution (31 nmol, 1.0 mol%) were added. The mixture was vortexed for 15 s, spun down in an Eppendorf tube centrifuge (13,000 *g*), and the pH was adjusted carefully to 7.0 by using 0.1 M NaOH and 0.1 M HCl stock solutions. The mixture was filled up to 250 µL with buffer (KP_i buffer, pH 7.0, 10 mM). Stock solutions of cinnamaldehyde (1.33 M in MeOH, HPLC grade) and nitromethane (6.61 M in MeOH, HPLC grade) were prepared. Subsequently, 2.5 µL of the nitromethane stock solution (16.5 µmol, 5.0 eq) and 2.5 µL of the aldehyde stock solution (3.3 µmol, 1.0 eq) were added to the catalyst solution. 250 µL of the respective organic solvent (C_6D_6, $CDCl_3$, EtOAc, THF, MeOH, MeCN, or DMSO) were added (appropriate amounts of MeOH and buffer for the 10% and 25% runs). The Eppendorf tube was placed in a Falcon tube, which was placed inside an incubator shaker (50 rpm, 25 °C) for 18 h. In case of C_6D_6 and $CDCl_3$, the phases were separated, the aqueous phase was extracted with the respective solvent (2 × 375 µL), and the combined organic phases were transferred into an NMR tube. This was directly subjected to ^{1}H NMR analysis. In each other case, the phases were separated, the aqueous phase was extracted (3 × 500 µL EtOAc, 3 × 500 µL $CHCl_3$), and the organic phases were combined. The volatiles were removed under reduced pressure, the residue was taken up in 700 µL $CDCl_3$, and transferred to an NMR tube. This was directly subjected to ^{1}H NMR

analysis. The product conversion was estimated by measuring the integral ratio between the substrate double bond and product CH_2 group.

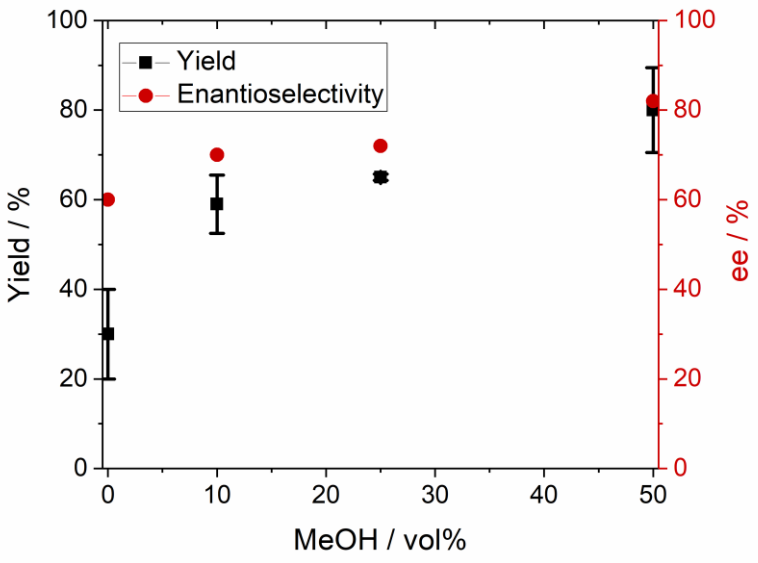

Graph S1. Plot of the effect of the vol% of MeOH in the catalyzed reaction by **Sav-7** on yield and enantioselectivity.

Exemplary ^1H NMR Spectrum after Extraction of the Buffer Reaction Mixture

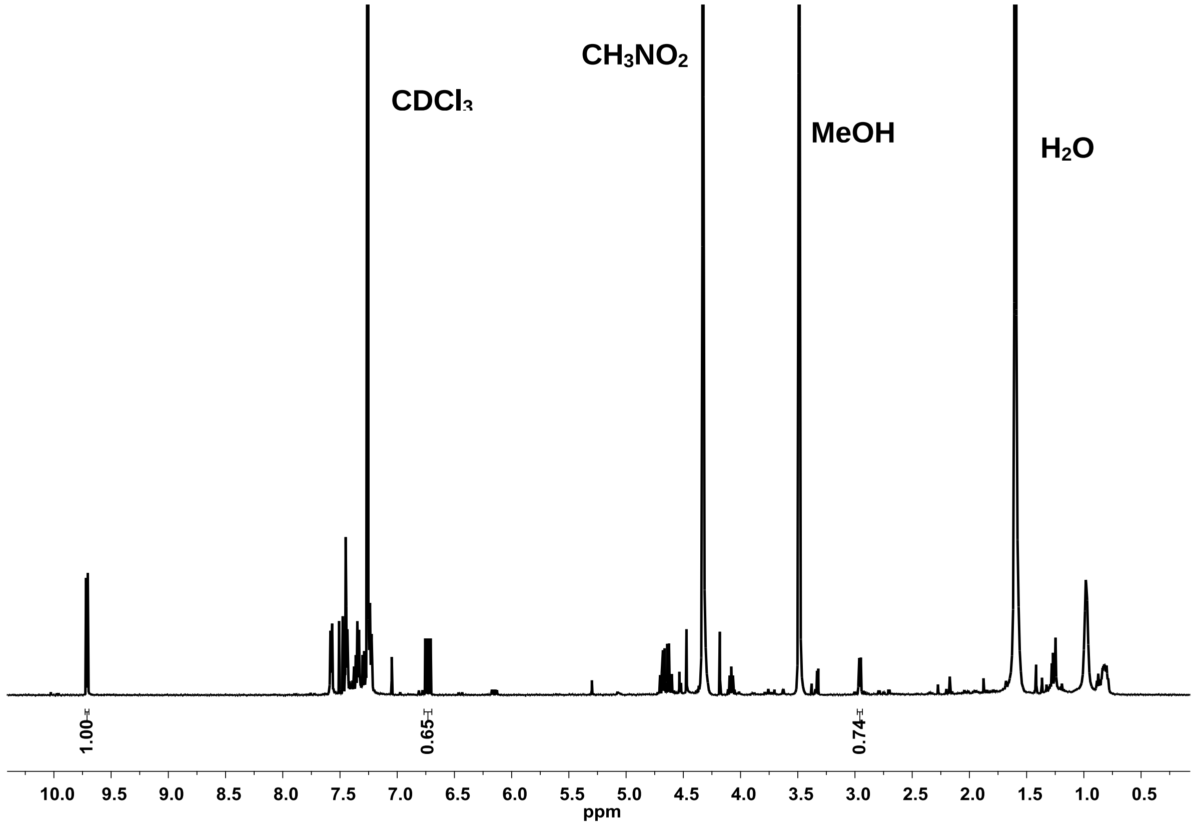

Exemplary ^1H NMR Spectrum after Extraction of the Biphasic Reaction Mixture

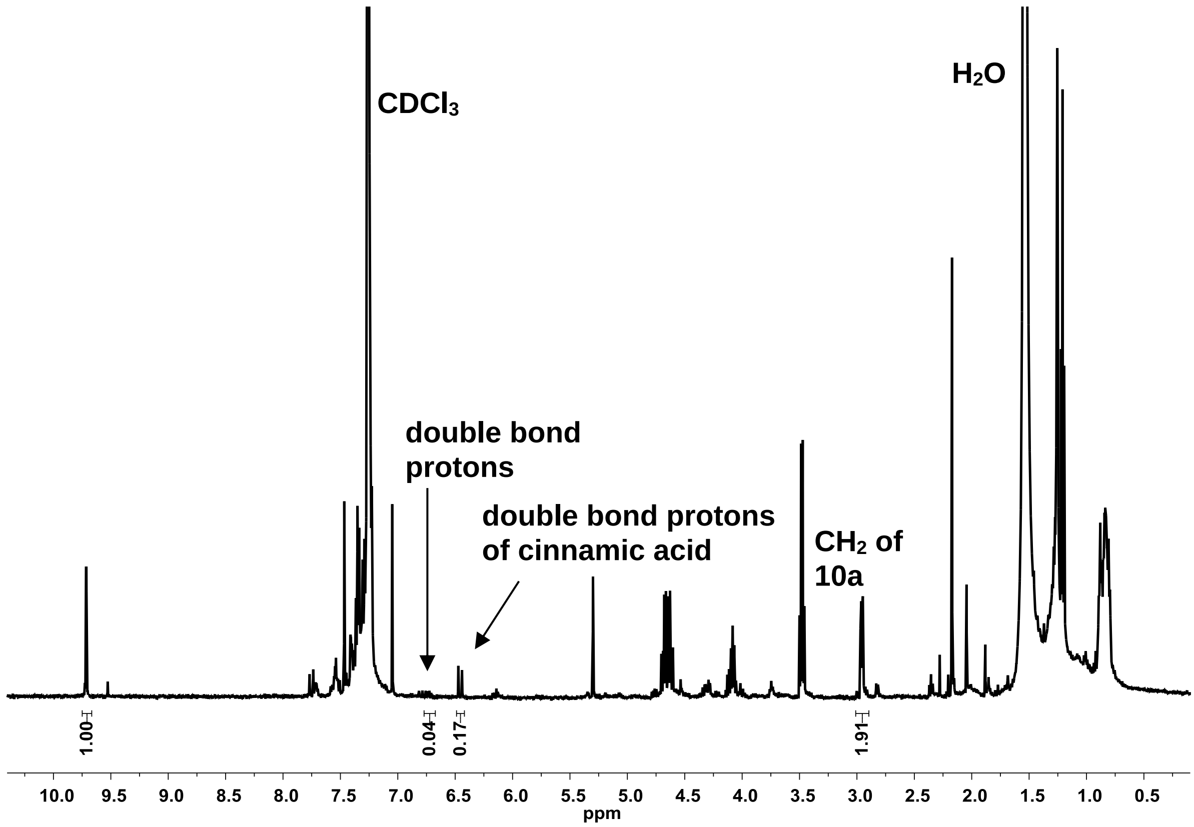

5 Experimental Details for the Catalyzed Michael Addition of Nitromethane to Cinnamaldehyde for Chiral Analysis

5.1 Half-Micro Scale Reactions for Product Isolation and Analysis

The reactions were performed as stated in 4.2. After checking the conversion by ^{1}H NMR, the aqueous phase was additionally extracted with CH_2Cl_2 (3 × volume of aqueous phase), the organic phases including the NMR sample were combined, dried over Na_2SO_4, and the solvent was removed. The crude material was purified by preparative TLC (*n*-hexane:EtOAc 75:25) using a complete sheet. The part containing **10** (checked via racemic reference sample by UV fluorescence deletion and permanganate stain) was cut out, the silica scratched from the aluminium plate, and stirred in CH_2Cl_2 for several minutes. The silica was filtered off, washed with CH_2Cl_2, the filtrate was concentrated under reduced pressure, and the purified product was stored at –23 °C under inert atmosphere until further usage

The purified compounds **10** were converted to alcohols **11** according to Ref [3] via reduction with $NaBH_4$, using methanol as solvent instead of ethanol.

6 Experimental Details for the Structure Determination of Sav-7

6.1 Procedure for Crystallization of Sav-7

Crystals of **Sav**-**7** were grown by adapting protocols for the preparation of **Sav**-biotin crystals.[9] A solution of commercial **Sav** (*Prospec* 791, 10 mg/mL) was incubated with **7** (5 equivalents per active biotin binding site) for 10 minutes. Residual KP_i from the commercial lyophilised protein was removed by buffer exchange via spin column (10k MWCO) to produce a solution of **Sav**-**7** in deionised water (40 mg/mL). This was used as protein stock solution to prepare samples for crystallisation.

The crystallisation experiments were performed in a 24-well plate employing 4 different conditions in each quadrant of 6 wells, with slightly varied conditions within the quadrants (see Table S1). All crystals were grown using the hanging-drop vapour-diffusion method. Each well was filled with 1 mL of the precipitant solution (Table S1). On the respective cover slip, three drops were produced by mixing the protein solution (1, 2, and 3 µL) with the same volume of precipitant solution. The assay was stored in an incubator at 20 °C and the crystals appeared within 10 days.

Table S1. Overview of crystallization conditions used in the 24 well plate.

Precipitant solution	mg/mL protein, % PEG 8000			Precipitant solution	mg/mL protein, % $(NH_4)_2SO_4$		
0.1 M HEPES, pH 7.5	25, 15	25, 16	25, 17	0.1 M NaOAc, pH 4.5, 0.2 M NaCl	26, 30	26, 35	26, 40
	30, 15	30, 16	30, 17		35, 30	35, 35	35, 40
Precipitant solution	mg/mL protein, % $(NH_4)_2SO_4$			Precipitant solution	mg/mL protein, % MPD		
5% 2-Propanol	12.5, 55	12.5, 60	12.5, 65	Deionized water	25, 49	25, 52	25, 55
	25, 55	25, 60	25, 65		30, 49	30, 52	30, 55

6.3 X-ray data collection, processing and structure solution

Crystals grown from various drops were fished and flash-frozen with liquid nitrogen. Diffraction data were collected at 100 K at beamlines I04 of the Diamond Light Source, Didcot, UK, with all images integrated with XDS[10] and scaled with Aimless[11] in xia2. The crystals grown in 52% MPD gave the best resolution. The structures were solved by molecular replacement using MOLREP[12] in CCP4i2,[13] using PDB 1mk5 as a search model. Model was refined numerous cycles with maximum likelihood refinement using REFMAC[14] and manually corrected using COOT.[15]

6.4 Crystallographic Details for the Obtained Crystal Structure of Sav-7

Table S2 Data collection and refinement statistics by Molecular replacement

	tSav-7
**PDB code**	6GH7
**Data collection**	
Space group	$P2_1$
Cell dimensions	
a, *b*, *c* (Å)	50.63, 98.50, 52.92
α, β, γ (°)	90.00, 112.67, 90.00
Resolution (Å)	98.49 (1.08) *
R_{merge}	0.075 (0.479)
$I/\sigma I$	6.2 (1.4)
Completeness (%)	97.4 (95.3)
Redundancy	3.7 (3.7)
**Refinement**	
Resolution (Å)	1.08
No. reflections	198738/9761
$R_{work/}$ R_{free}	0.159/0.185
No. atoms	
Protein	6712
Ligand/ion	180
Water	683
B-factors	
Protein	14.33
Ligand/ion	11.4
Water	30.8
R.m.s deviations	
Bond lengths (Å)	0.0137
Bond angles (º)	1.904

*Highest resolution shell is shown in parenthesis.

7 Experimental Details for the Molecular Dynamics Simulations of the Model Michael Reaction with Sav-7 and Sav-8

7.1 Computer simulations of the active catalysts Sav-7 and Sav-8

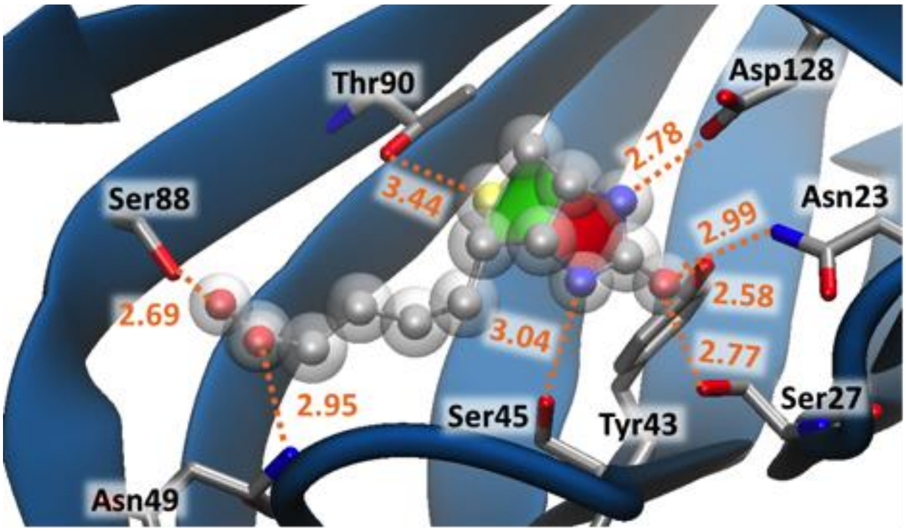

Scheme S1. Active site of Streptavidin with bound biotin together with highlighted residues involved in direct interaction based on the X-Ray structure. Distances are given in Å.

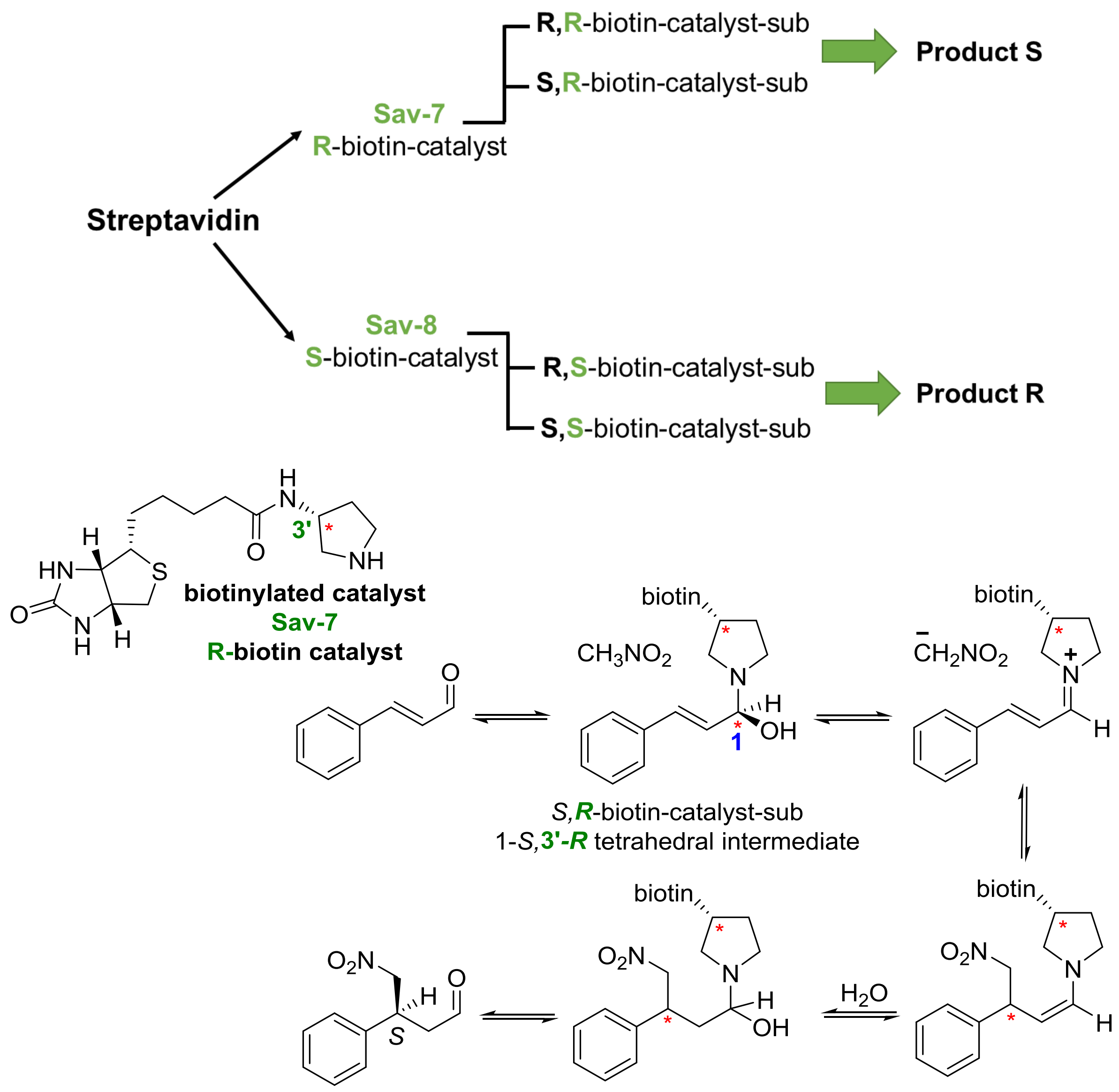

Scheme S2. Schematic representation of the possible formation of Streptavidin complex with biotin-catalyst and cinamaldehyde.

System setup. The origin of atoms coordinates of streptavidin with bound biotin was adapted from X-Ray structure as available in Protein Data Bank (PDB ID 1STP).[16] Biological assembly of homo-4-mer was generated using the PDBe PISA v1.52 software.[17] The correctness of the protein side-chains was confirmed using the SCit tool.[18] Subsequently, one of the oxygen atoms of the carboxylic acid of biotin was mutated to nitrogen and the pyrrolidine catalyst (**7** and **8**) was covalently bound to it forming biotin-catalyst complexes in *R* or *S* conformation, respectively (see Scheme S1 and S2). Missing hydrogen atoms were added using the t-LEAP module of Amber Tools program. The protonation state of titratable amino acids in pH 7 was determined using pKa results calculated using PROPKA ver. 3.1[19] available on PDB2PQR server.[20] Obtained results indicated that Asp, Glu, Tyr, Lys and Arg residues are present in their standard protonation form together with His127 (pKa 1.92) in which the proton was added in ε-position. Only His87 presented non-standard behavior (with calculated pK_a of 7.13) and was protonated in both δ- and ε-positions in all four chains. Neutralization of the total charge of the system was made by addition of 4 sodium cations (Na+) in the electrostatically most favorable positions. Finally, the system was placed in orthorhombic box of TIP3P water with size of 87 × 86 × 76 Å^3.

Molecular dynamics: Optimization and molecular dynamics (MD) simulations were performed at molecular mechanistic (MM) level using NAMD software.[21] The behaviour of the system was controlled by using AMBER ff-03 parameters.[22] NPT MD of 10 ns for the **Sav-7** and **Sav-8** models with time steps of 1 fs at 300 K were carried out after previous optimization, heating (from 0 to 300 K with 0.001 K temperature increment) and equilibration processes of 100ps. The constant temperature and pressure were controlled using the Langevin piston method.[23] The periodic boundary conditions (PBC) using the particle mesh Ewald method were applied. A cut-off for nonbonding interactions was applied using a smooth switching function with between 14.5 to 16 Å.

After confirming that the two systems were equilibrated (see Fig. S1), the last structures from 10 ns MDs were used in order to build up four different models of the intermediate in which cinnamaldehyde is covalently bound to the nitrogen of catalyst and the proton previously bound to nitrogen is transferred to oxygen atom of carbonyl group of the substrate, as taking place in the full catalytic process (see Scheme S2). Subsequently, 4 ns of NPT MDs were ran for all 4 models with the same conditions of calculations as described above. Missing atom types, charges and parameters for *R*-biotin-catalyst **7**, *S*-biotin-catalyst **8** and four complexes of biotin-catalyst-cinamaldehyde were generated using the Antechamber software package together with a general AMBER force field (GAFF)[24] available in Amber Tools.

Table S3. Atom types, charges and parameters obtained for *R*-biotin-catalyst **7**, *S*-biotin-catalyst **8** and four complexes of biotin-catalyst-cinamaldehyde generated using the Antenchamber package included in AmberTools.

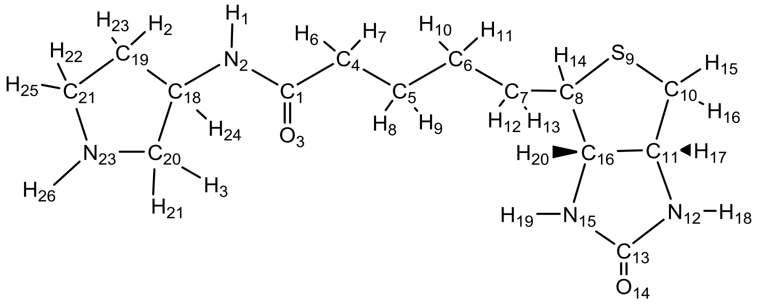

Biotin-catalyst (**7**)			Biotin-catalyst (**8**)		
Atom name	Atom type	Charge	Atom name	Atom type	Charge
C1	c	0.6591	C1	c	0.6561
N2	n	-0.5749	N2	n	-0.5709
O3	o	-0.6091	O3	o	-0.6001
C4	c3	-0.1494	C4	c3	-0.1534
C5	c3	-0.0694	C5	c3	-0.0744
C6	c3	-0.0854	C6	c3	-0.0854
C7	c3	-0.0704	C7	c3	-0.0594
C8	c3	-0.0076	C8	c3	0.0144
S9	ss	-0.2972	S9	ss	-0.2902
C10	c3	-0.0433	C10	c3	-0.0393
C11	c3	0.0577	C11	c3	0.0557
N12	n	-0.5139	N12	n	-0.5049
C13	c	0.7505	C13	c	0.7575
O14	o	-0.6175	O14	o	-0.6165
N15	n	-0.5189	N15	n	-0.5459
C16	c3	0.0617	C16	c3	0.0737
C18	c3	0.0727	C18	c3	0.0897
C19	c3	-0.1254	C19	c3	-0.1154
C20	c3	0.1268	C20	c3	0.1218
C21	c3	0.1408	C21	c3	0.1438
N23	n3	-0.7932	N23	n3	-0.7822
H25	h1	0.0407	H1	hn	0.3085
H26	hn	0.3458	H2	hc	0.0427
H1	hn	0.3045	H3	h1	0.0417
H2	hc	0.0497	H25	h1	0.0367
H6	hc	0.0657	H26	hn	0.3408
H3	h1	0.0517	H6	hc	0.0627
H7	hc	0.0627	H7	hc	0.0627
H8	hc	0.0677	H8	hc	0.0587
H9	hc	0.0437	H9	hc	0.0577
H10	hc	0.0487	H10	hc	0.0447
H11	hc	0.0637	H11	hc	0.0527
H12	hc	0.0587	H12	hc	0.0477
H13	hc	0.0547	H13	hc	0.0727
H14	h1	0.0867	H14	h1	0.0797
H15	h1	0.0867	H15	h1	0.0847
H16	h1	0.0777	H16	h1	0.0757
H17	h1	0.0897	H17	h1	0.0857
H18	hn	0.3115	H18	hn	0.3075
H19	hn	0.3175	H19	hn	0.3265
H20	h1	0.0897	H20	h1	0.0637
H21	h1	0.0667	H21	h1	0.0797
H22	h1	0.0607	H22	h1	0.0687
H23	hc	0.0617	H23	hc	0.0797
H24	h1	0.0997	H24	h1	0.0437
**Missing parameters:**					
IMPROPER					
c3-n -c -o	10.5	180.0	2.0	General improper torsional angle (2 general atom types)	
n -n -c -o	10.5	180.0	2.0	General improper torsional angle (2 general atom types)	

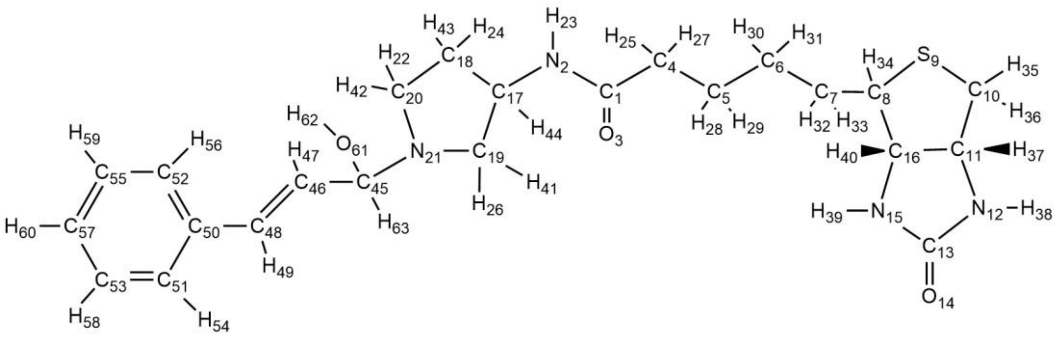

		**Sav-7-*R*-sub**	**Sav-7-*S*-sub**	**Sav-8-*R*-sub**	**Sav-8-*S*-sub**
Atom name	Atom type	Charges			
C1	c	0.6571	0.6581	0.6601	0.6591
N2	n	-0.5779	-0.5759	-0.5679	-0.5719
O3	o	-0.6081	-0.6181	-0.6091	-0.6161
C4	c3	-0.1554	-0.1494	-0.1504	-0.1494
C5	c3	-0.0794	-0.0694	-0.0764	-0.0694
C6	c3	-0.0914	-0.0854	-0.0864	-0.0854
C7	c3	-0.0704	-0.0704	-0.0724	-0.0704
C8	c3	-0.0176	-0.0076	-0.0056	-0.0036
S9	ss	-0.2892	-0.2952	-0.2882	-0.2892
C10	c3	-0.0443	-0.0443	-0.0213	-0.0213
C11	c3	0.0597	0.0567	0.0707	0.0697
N12	n	-0.5149	-0.5129	-0.5399	-0.5399
C13	c	0.7485	0.7495	0.7555	0.7555
O14	o	-0.6125	-0.6175	-0.6115	-0.6145
N15	n	-0.5189	-0.5179	-0.5089	-0.5079
C16	c3	0.0567	0.0617	0.0557	0.0557
C17	c3	0.0807	0.0797	0.0807	0.0937
C18	c3	-0.1124	-0.1194	-0.1224	-0.1114
C19	c3	0.1298	0.1368	0.1398	0.1478
C20	c3	0.1548	0.1468	0.1588	0.1568
N21	n3	-0.7376	-0.7306	-0.7506	-0.7236
H23	hn	0.3165	0.3235	0.3095	0.3135
H24	hc	0.0617	0.0567	0.0557	0.0547
H26	h1	0.0547	0.0637	0.0707	0.0447
H22	h1	0.0457	0.0407	0.0487	0.0407
H25	hc	0.0847	0.0657	0.0817	0.0667
H27	hc	0.0667	0.0647	0.0567	0.0627
H28	hc	0.0497	0.0667	0.0627	0.0667
H29	hc	0.0547	0.0427	0.0527	0.0437
H30	hc	0.0617	0.0487	0.0547	0.0497
H31	hc	0.0487	0.0647	0.0457	0.0627
H32	hc	0.0497	0.0587	0.0547	0.0567
H33	hc	0.0607	0.0537	0.0617	0.0557
H34	h1	0.0987	0.0857	0.0787	0.0837
H35	h1	0.0877	0.0867	0.1017	0.0997
H36	h1	0.0797	0.0767	0.0687	0.0677
H37	h1	0.0907	0.0887	0.0627	0.0617
H38	hn	0.3145	0.3115	0.3225	0.3205
H39	hn	0.3145	0.3175	0.3125	0.3125
H40	h1	0.0877	0.0887	0.0827	0.0827
H41	h1	0.0687	0.0657	0.0567	0.0727
H42	h1	0.0587	0.0627	0.0557	0.0527
H43	hc	0.0597	0.0597	0.0527	0.0617
H44	h1	0.1017	0.0977	0.0947	0.0947
C45	c3	0.3975	0.3945	0.4035	0.4195
H63	h2	0.0947	0.0947	0.0597	0.0497
O61	oh	-0.6418	-0.6338	-0.5938	-0.5948

H62	ho	0.402	0.4	0.409	0.41
C46	c2	-0.1962	-0.2082	-0.1852	-0.2422
C48	ce	-0.1002	-0.0732	-0.0952	-0.0872
H47	ha	0.15	0.132	0.136	0.14
H49	ha	0.122	0.142	0.126	0.125
C50	ca	-0.0688	-0.0668	-0.0628	-0.0678
C51	ca	-0.117	-0.114	-0.116	-0.116
C52	ca	-0.114	-0.116	-0.116	-0.113
C53	ca	-0.128	-0.131	-0.131	-0.132
H54	ha	0.14	0.134	0.133	0.132
C55	ca	-0.133	-0.132	-0.131	-0.131
H56	ha	0.133	0.13	0.134	0.135
C57	ca	-0.121	-0.122	-0.123	-0.121
H58	ha	0.139	0.134	0.133	0.133
H59	ha	0.133	0.133	0.133	0.134
H60	ha	0.134	0.133	0.133	0.134

Missing Parameters:

DIHEDRALS

```
c2-ce-ca-ca   1    2.550      180.000        2.000      same as X -c2-ca-X
ha-ce-ca-ca   1    2.550      180.000        2.000      same as X -c2-ca-X
```

IMPROPERS

```
c3-n -c -o       10.5       180.0      2.0       General improper torsional angle (2 general atom types)
n -n -c -o       10.5       180.0      2.0       General improper torsional angle (2 general atom types)
c3-ce-c2-ha       1.1       180.0      2.0       Using default value
c2-ca-ce-ha       1.1       180.0      2.0       Using default value
ca-ca-ca-ce       1.1       180.0      2.0       Using default value
ca-ca-ca-ha       1.1       180.0      2.0       General improper torsional angle (2 general atom types)
```

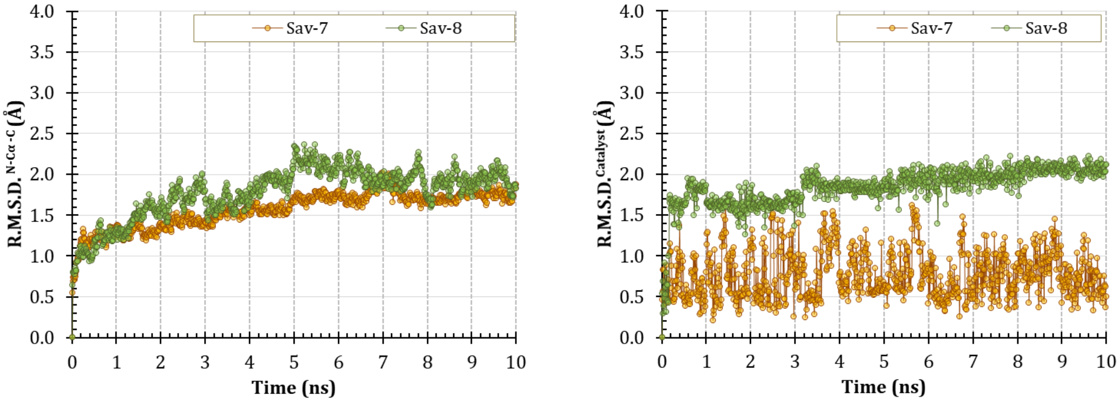

Figure S1. Time evolution of RMSD of atoms position of **A)** protein backbone and **B)** biotin-catalyst for **Sav-7** and **Sav-8** model along 10 ns of MD simulations.

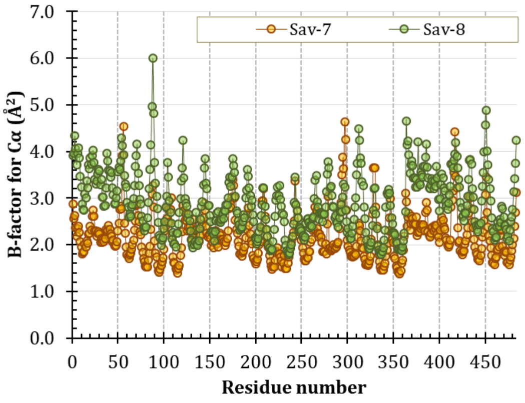

Figure S2. Displacement of Cα atoms of protein backbone from their original position (B-factor) along 10 ns of MD simulations for **Sav-7** and **Sav-8.**

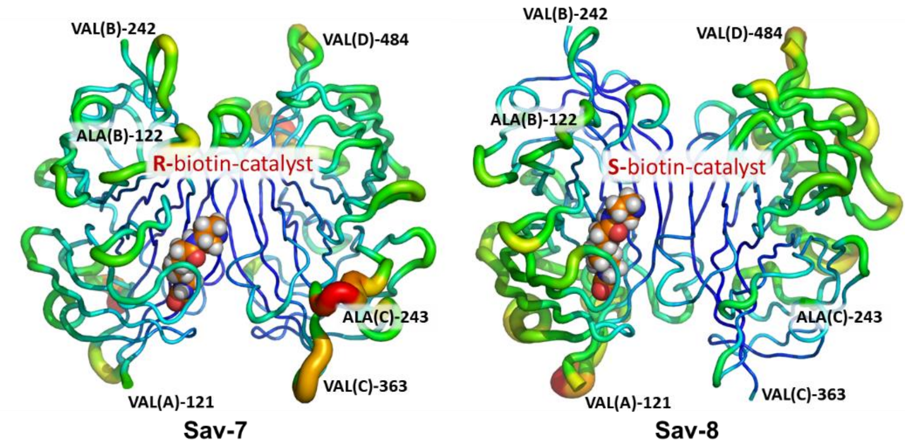

Figure S3. Displacement of atoms of protein from their original position (B-factor) along 10ns of MD simulations for **Sav-7** and **Sav-8**.

A. Sav-7

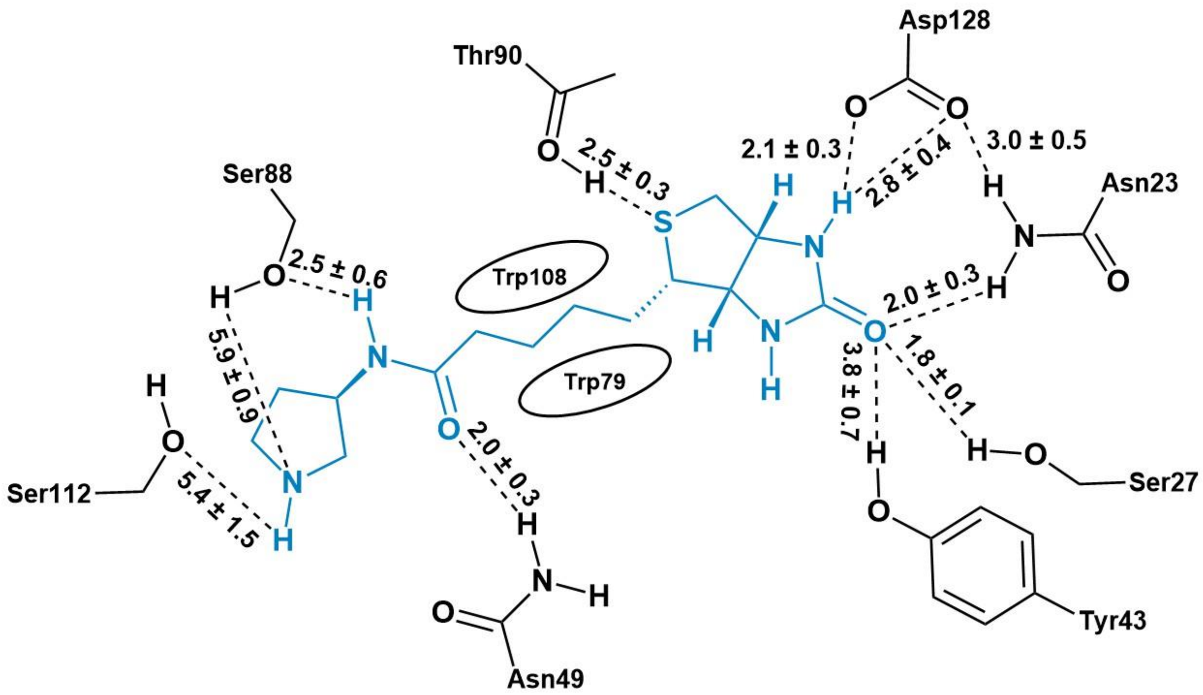

B. Sav-8

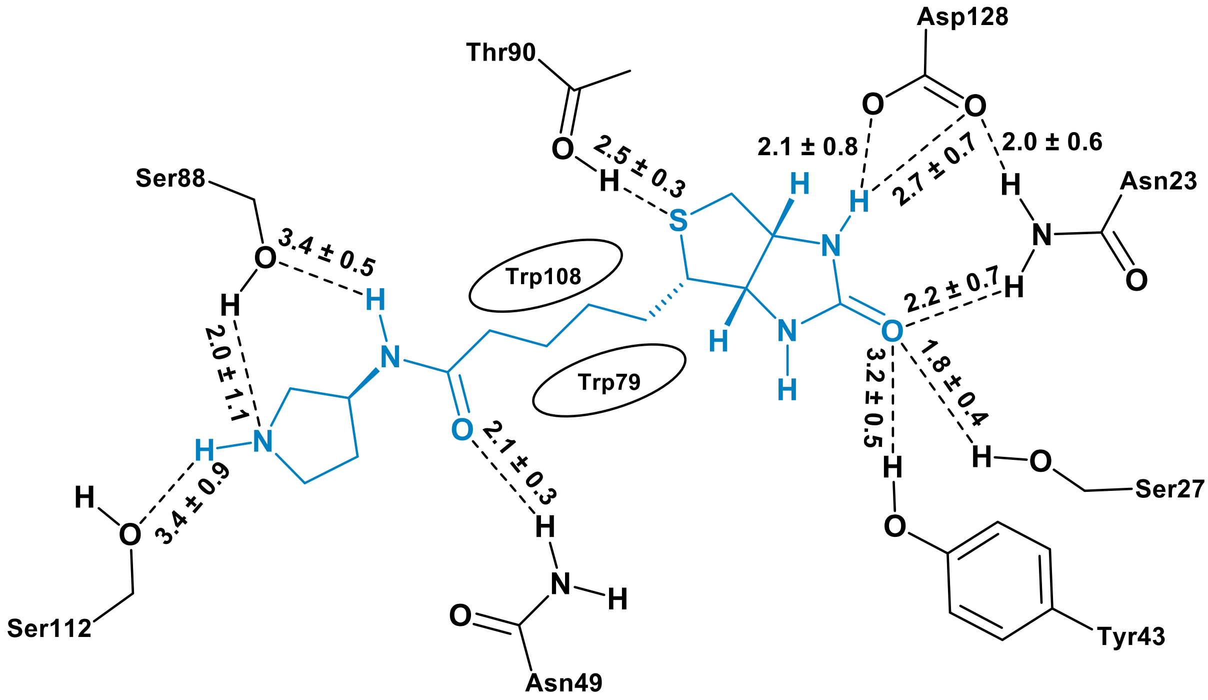

Figure S4. Schematic representation of important interactions between residues of the active site and both isomers of the biotin-catalyst. Distances (in Å) computed as average over the last 5 ns of the MD simulation.

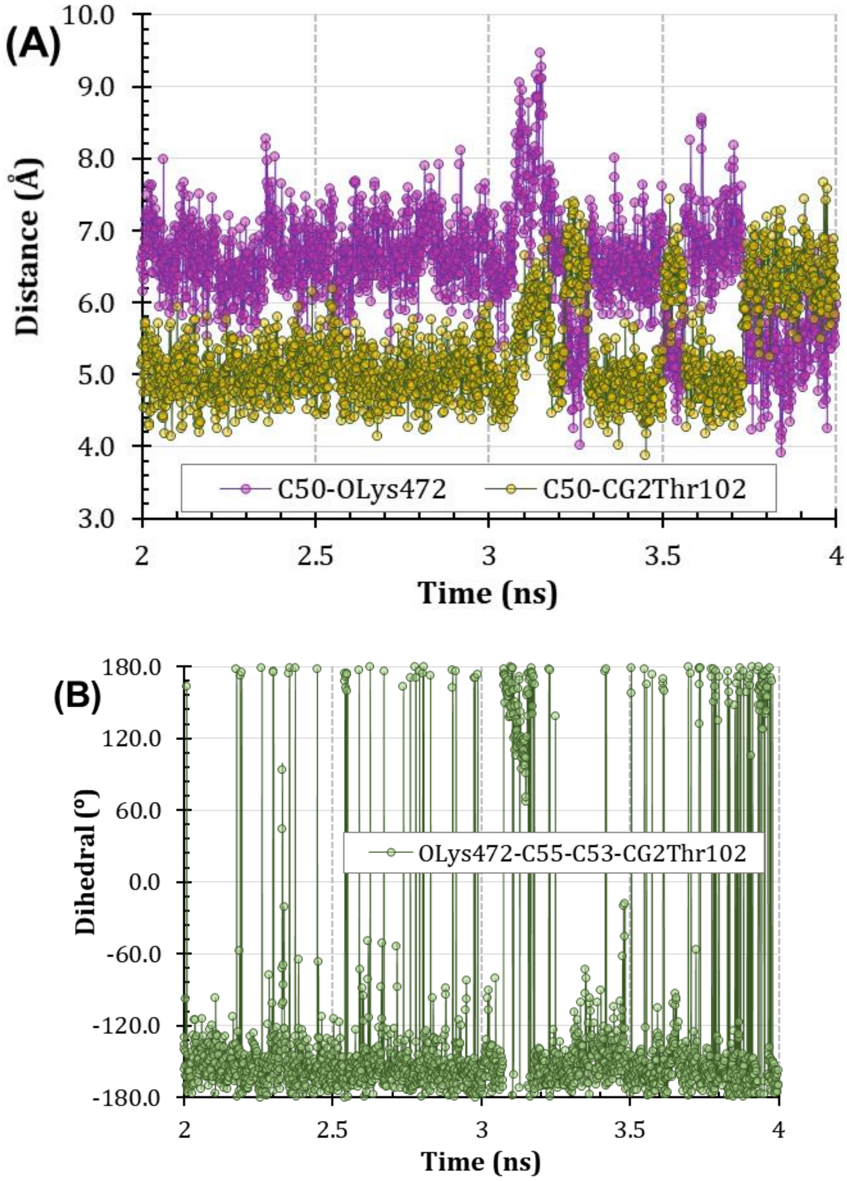

Figure S5. Population distribution of key distances (A) and dihedral angle (B) obtained along the MD simulations for the in **Sav**-**8**-*R* model. The selected distances on panel A indicate the relative distances of cinnamaldehyde to the cavity of the protein, while the dihedral angles indicate the relative orientation: negative values represent orientation that would form *R*-**10a** while positive values would favor the attack that would form *S*-**10a**. According to the population analysis, 89% of structures along the last 2 ns MD are in orientations that favor the Nu: attack to generate the *R*-product.

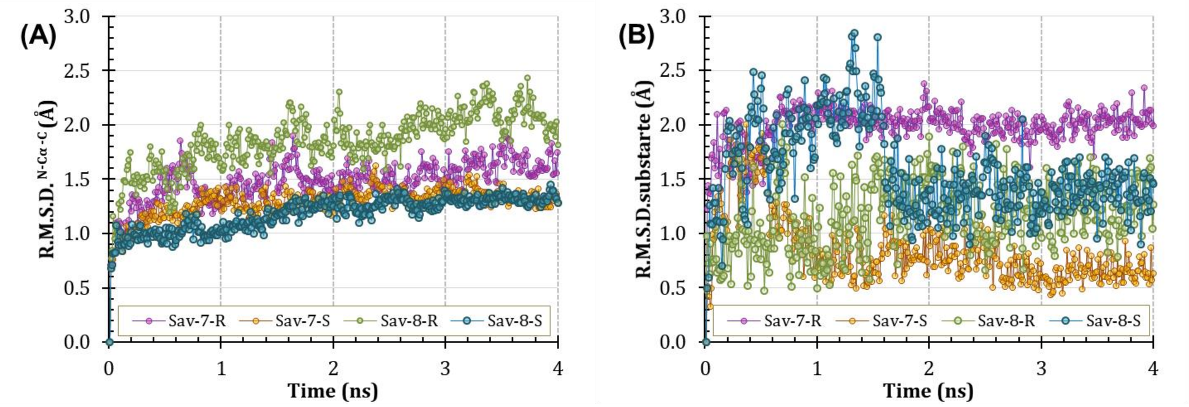

Figure S6. Time dependence of RMSD computed from the 4 ns MD simulations for the four stereoisomers of **Sav**-**7**-*R*. **Sav**-**7**-*S*, **Sav**-**8**-*R* and **Sav**-**8**-*S* computed for the position of: **A**) the atoms of the protein backbone; and **B**) the atoms of the organocatalyst complex with the cinnamaldehyde.

Averaged values of the RMSD of the atomic positions of the protein backbone computed in range of MD between 2 ns to 4 ns is 1.563 ± 0.122 Å, 1.352 ± 0.071 Å, 2.001 ± 0.166 Å and 1.293 ± 0.059 Å for **Sav**-**7**-*R*, **Sav**-**7**-*S*, **Sav**-**8**-*R* and **Sav**-**8**-*S*, respectively.

As shown in Fig S6B, the systems can be considered as equilibrated after 2 ns of MD. The calculated average RMSD values obtained from the last 2 ns of the MD simulations are equal to 1.997±0.105 Å, 0.695±0.142 Å, 1.251 ± 0.253 Å, and 1.376 ± 0.196 Å, **Sav**-**7**-*R*, **Sav**-**7**-*S*, **Sav**-**8**-*R* and **Sav**-**8**-*S*, respectively.

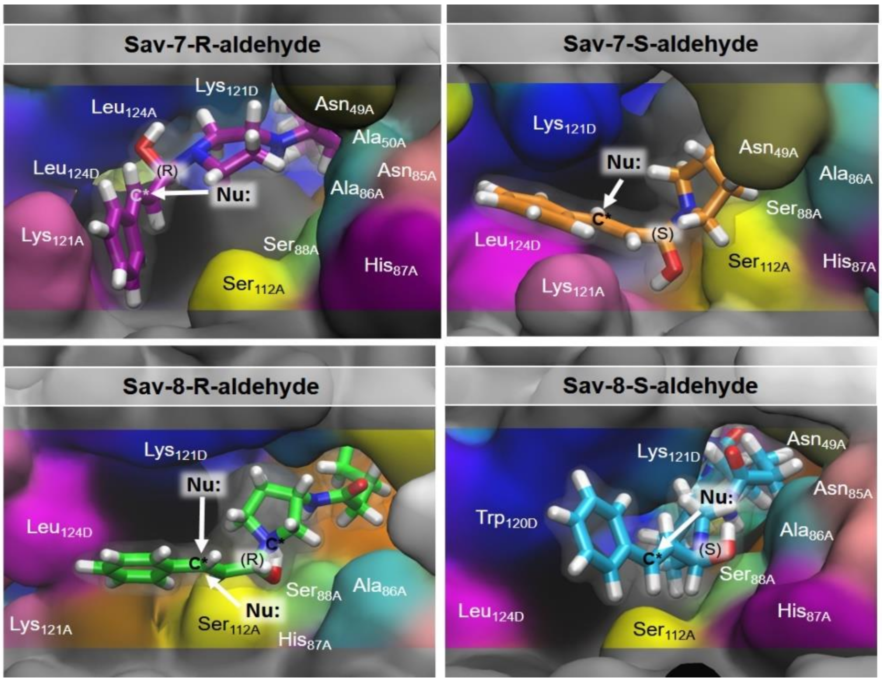

Figure S7. Representative snapshots of the tetrahedral intermediates obtained from reaction with **Sav-7** (top) and **Sav-8** (bottom). The arrow indicates the possible side for addition of a nucleophile.

8 NMR Spectra of Novel Compounds

8.1 (S)-4-Azidophenylalanine methyl amide

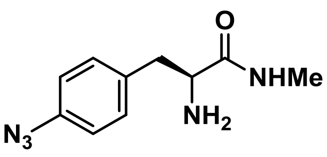

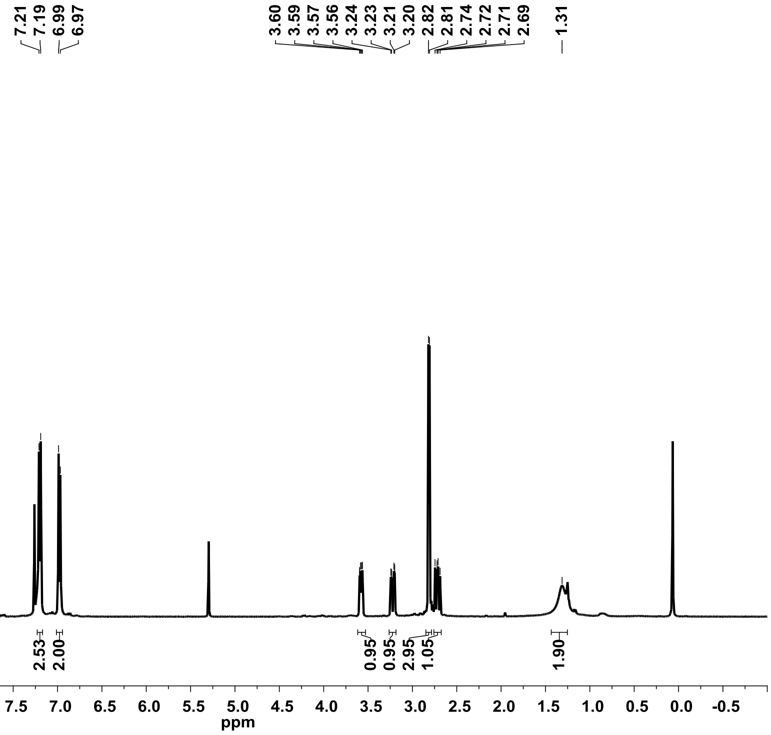

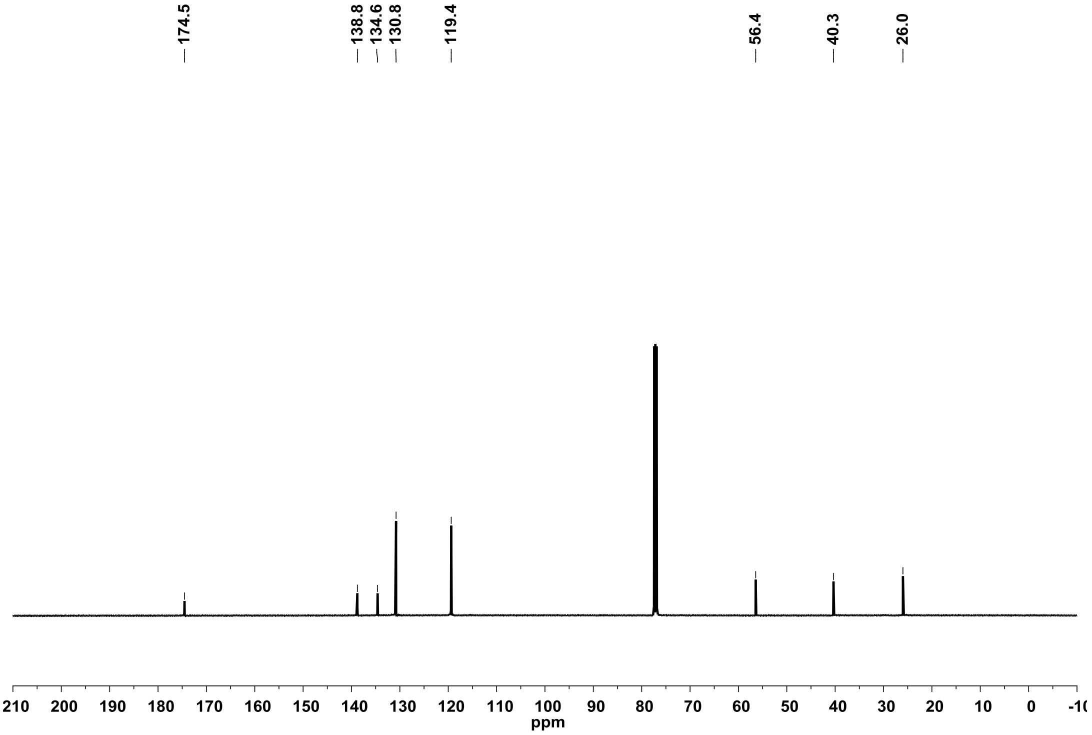

8.2 (*S*)-5-(4-Azidobenzyl)-2,2,3-trimethylimidazolidin-4-one

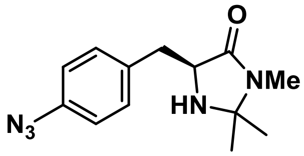

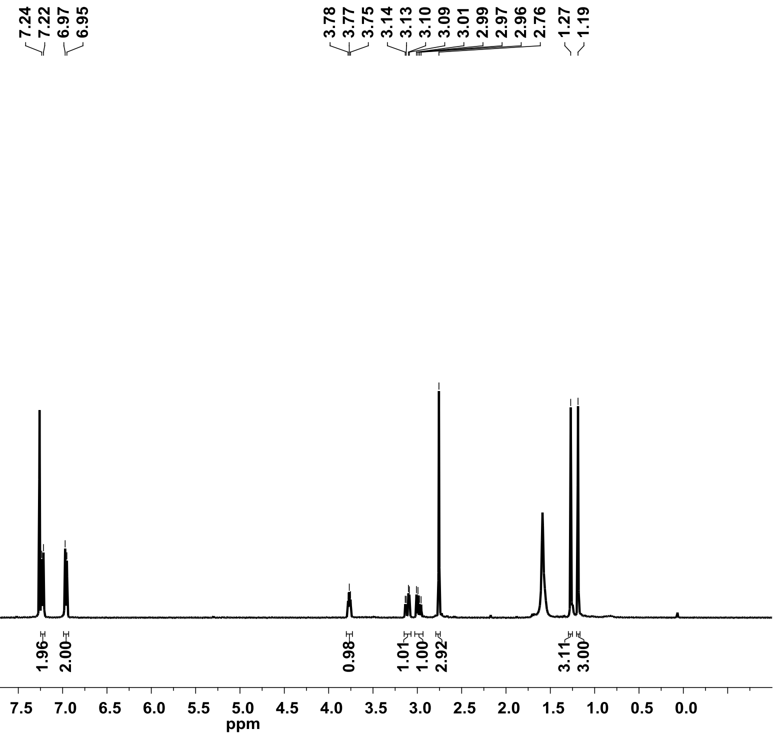

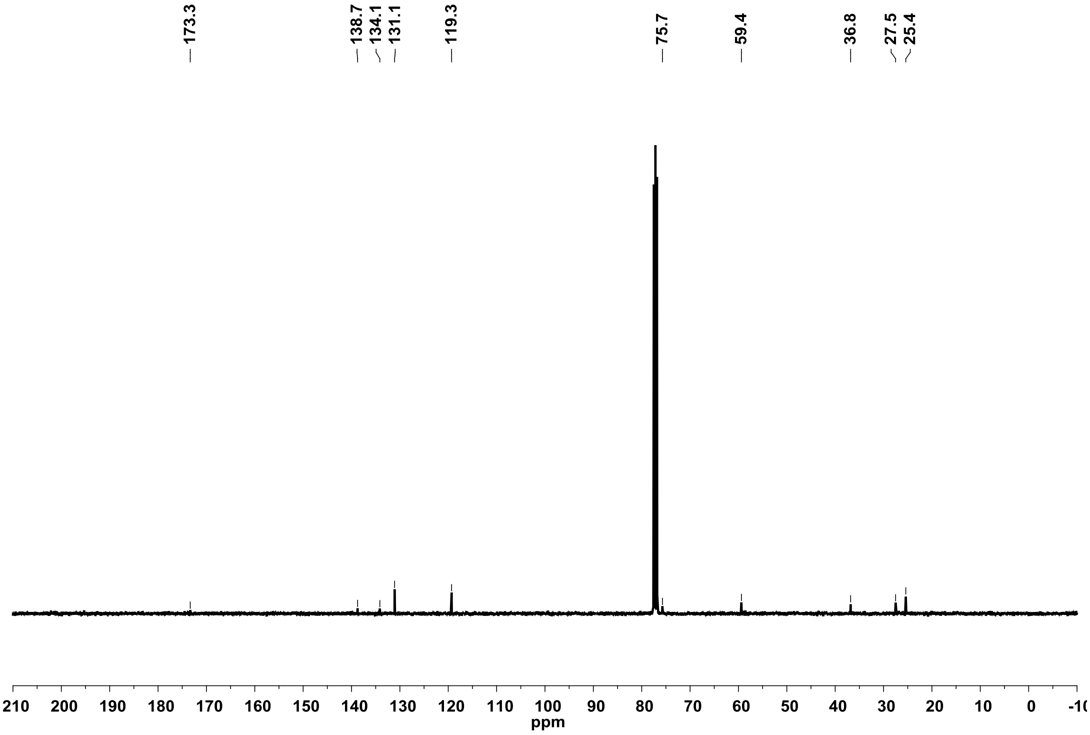

8.3 5-((3a*S*,4*S*,6a*R*)-2-oxohexahydro-1H-thieno[3,4-d]imidazol-4-yl)-N-((1-(4-(((*S*)-1,2,2-trimethyl-5-oxoimidazolidin-4-yl)methyl)phenyl)-1H-1,2,3-triazol-4-yl)methyl)pentanamide 1

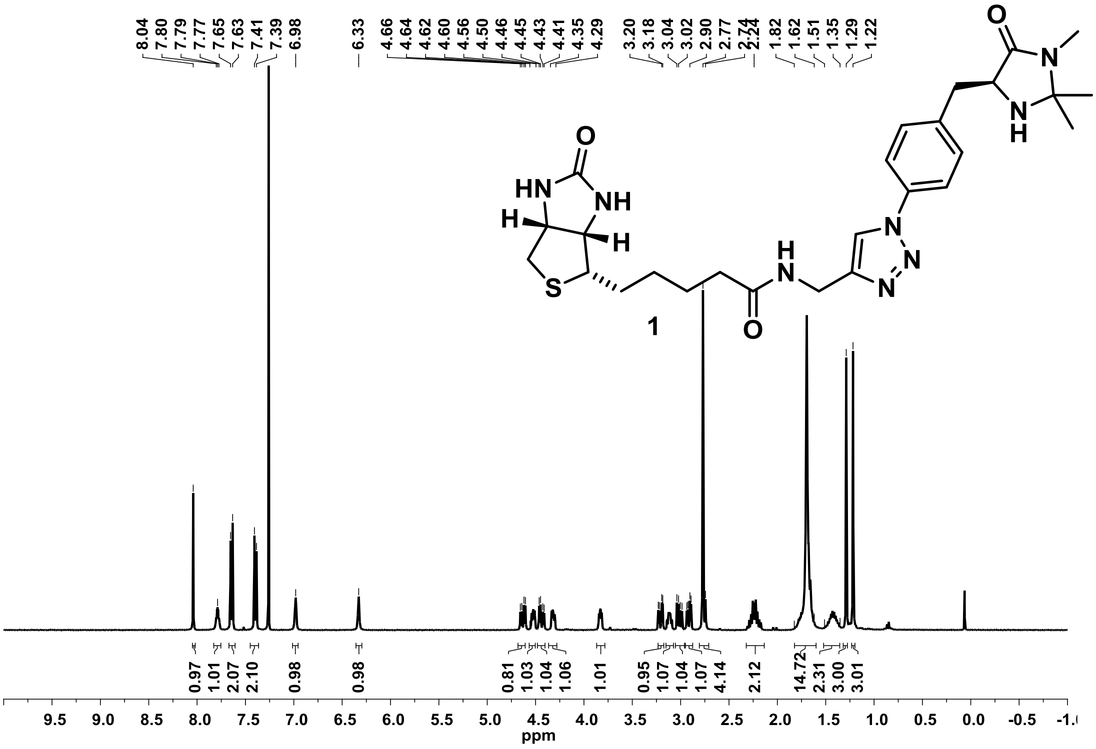

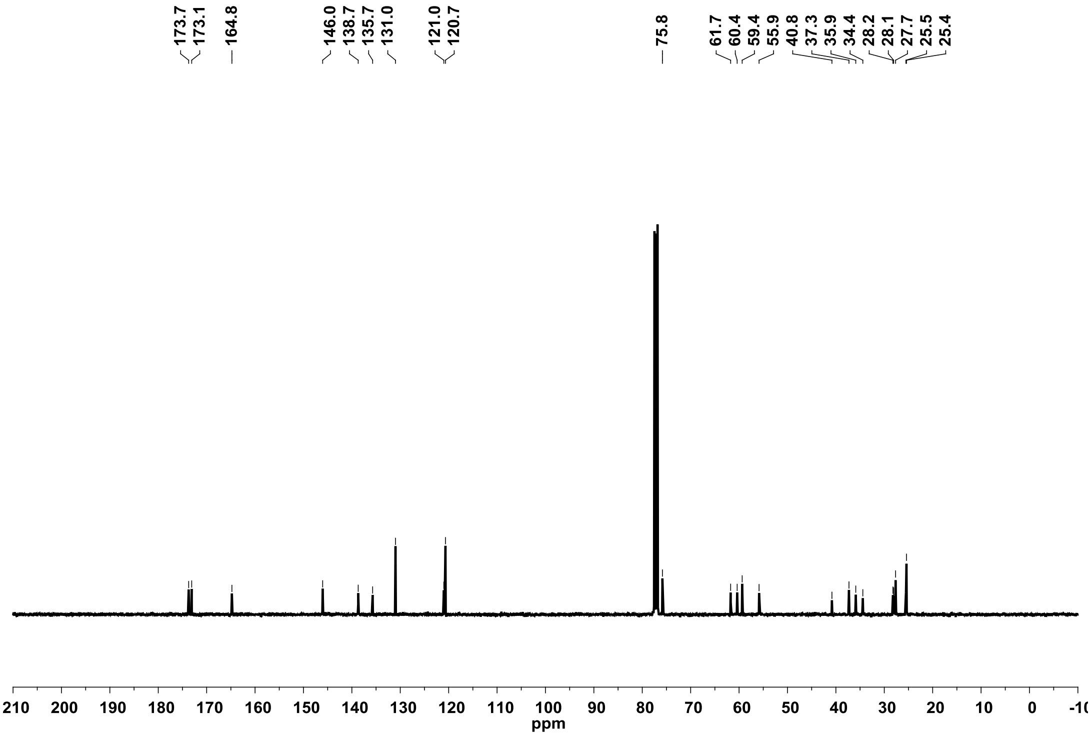

8.4 (2*S*,5*S*)-5-(4-azidobenzyl)-2-(*tert*-butyl)-3-methylimidazolidin-4-one

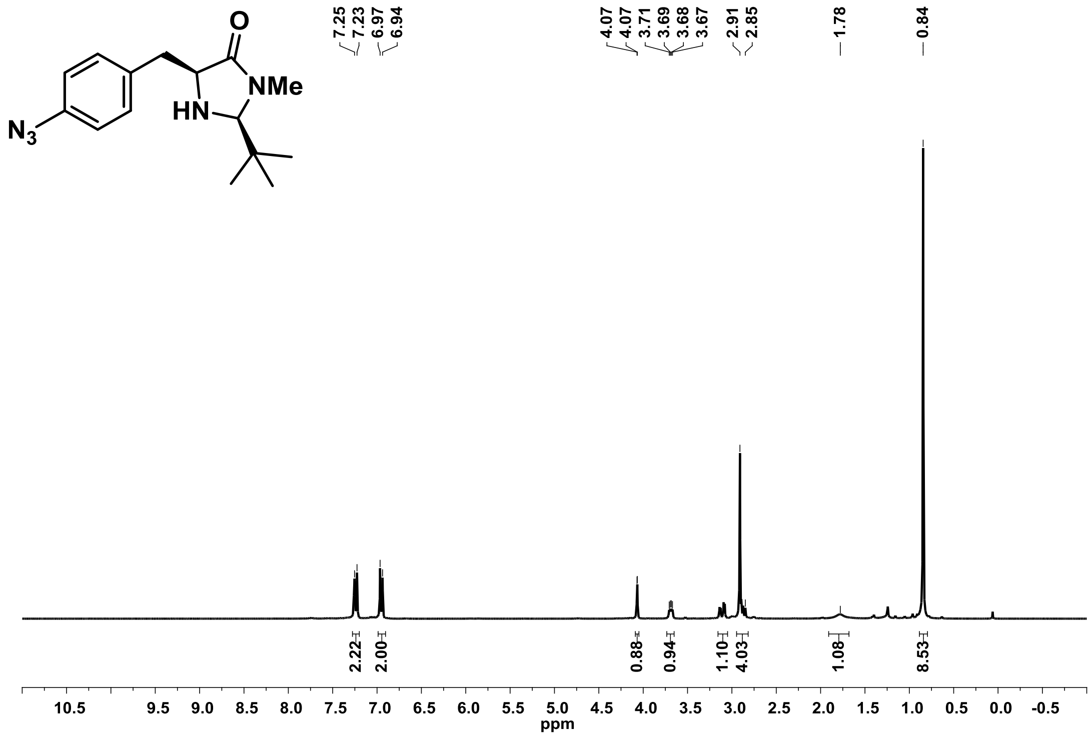

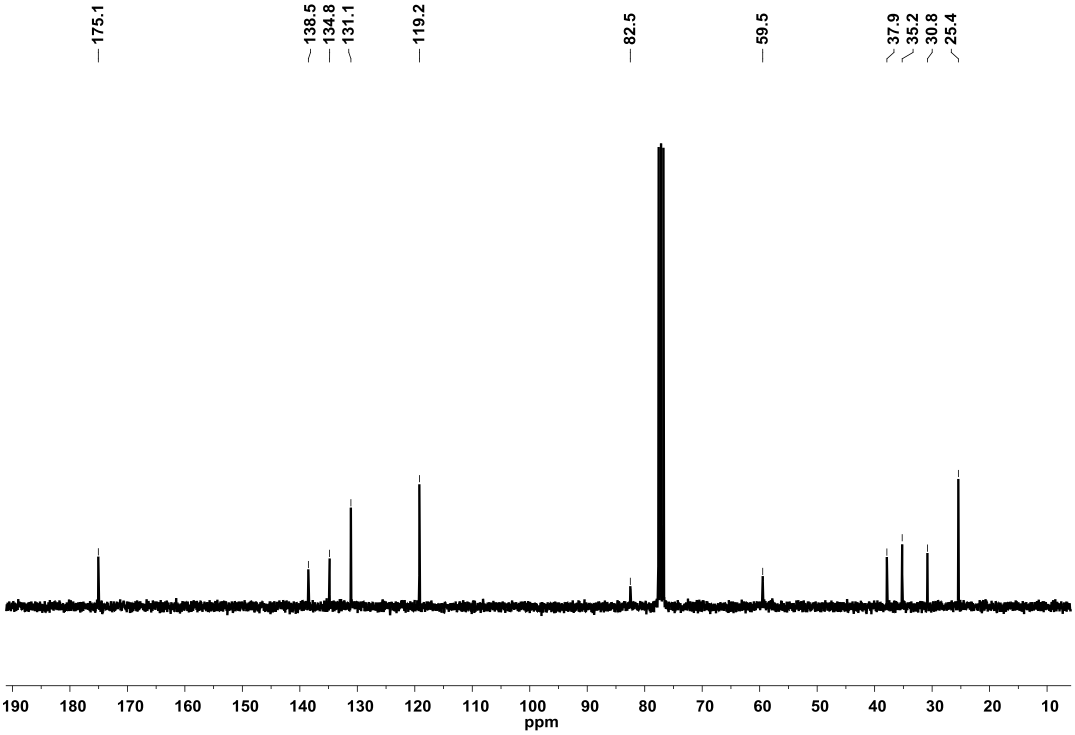

8.5 *N*-((1-(4-(((2*S*,4*S*)-2-(*tert*-butyl)-1-methyl-5-oxoimidazolidin-4-yl)methyl)phenyl)-1H-1,2,3-triazol-4-yl)methyl)-5-((3a*S*,4*S*,6a*R*)-2-oxohexahydro-1H-thieno[3,4-d]imidazol-4-yl)pentanamide 2

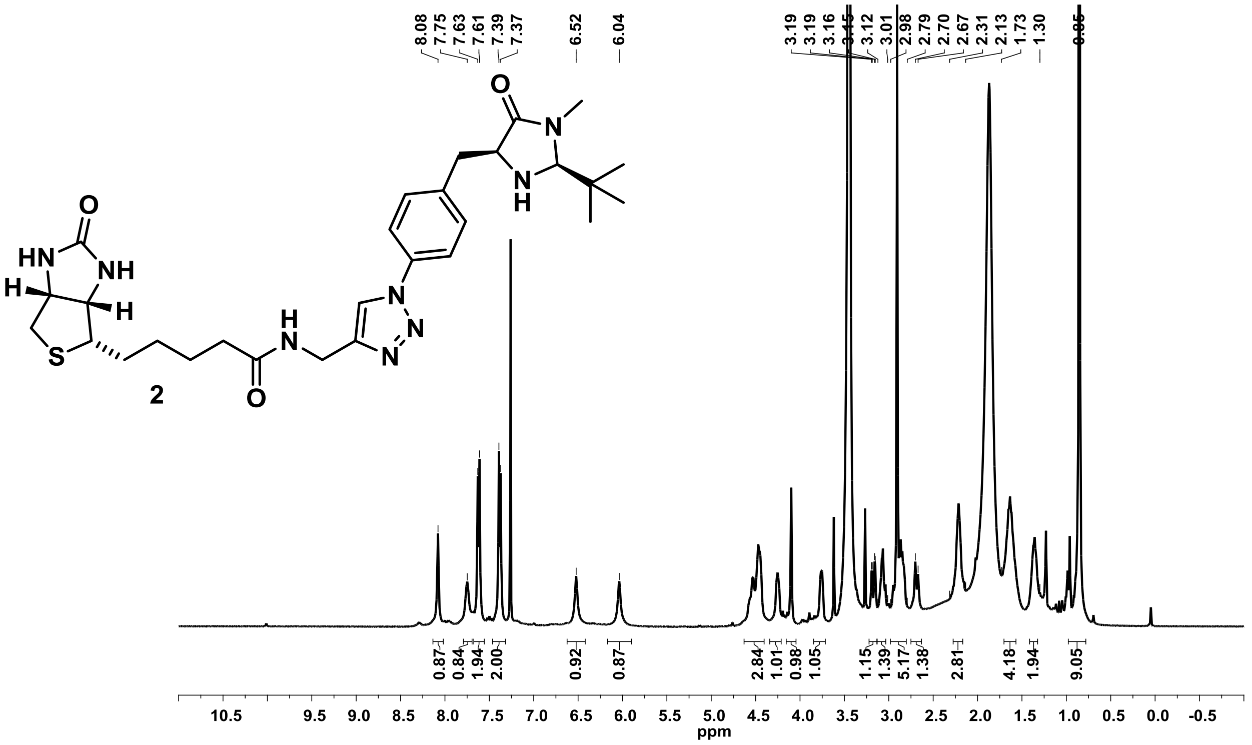

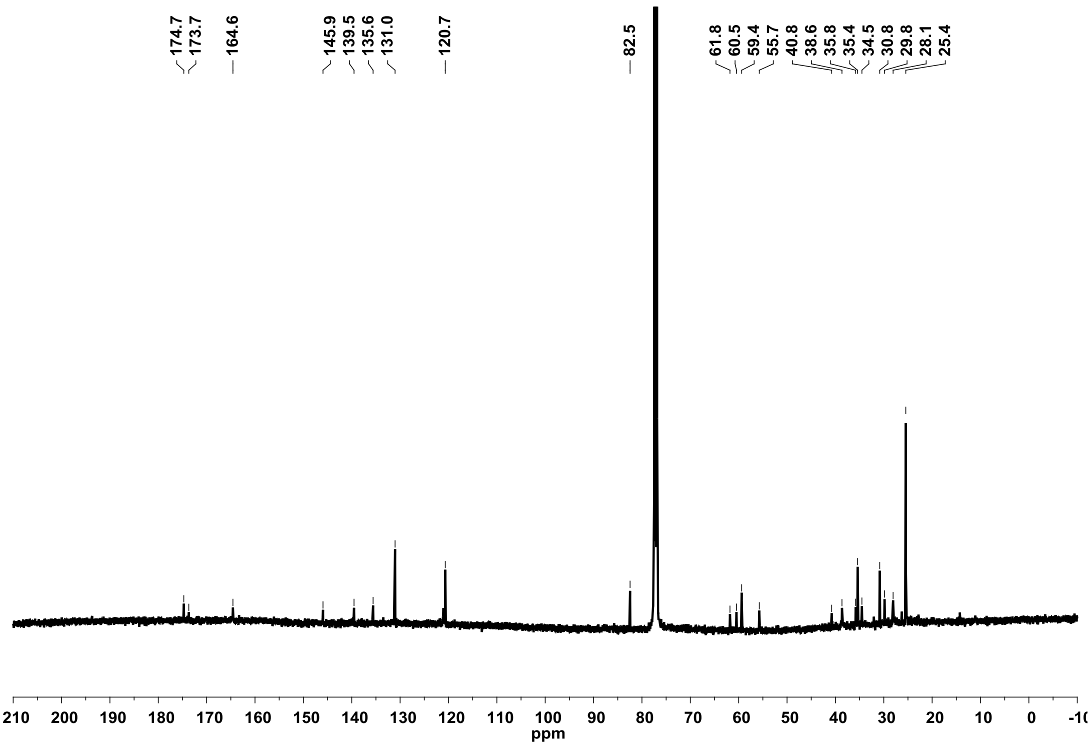

8.6 (*S*)-*N*-Methyl-2-amino-4-azidobutanamide

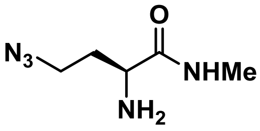

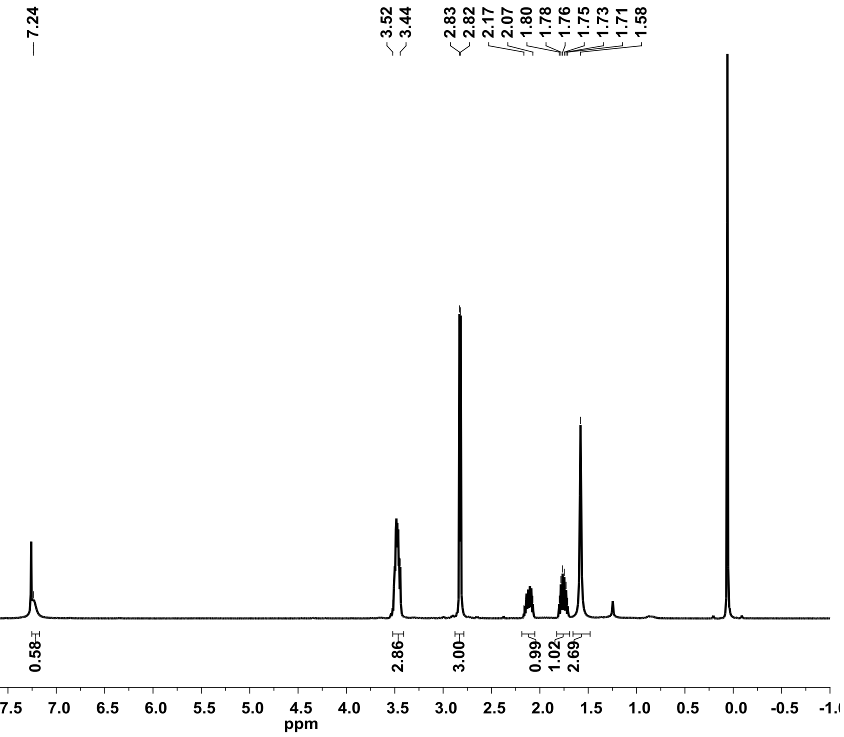

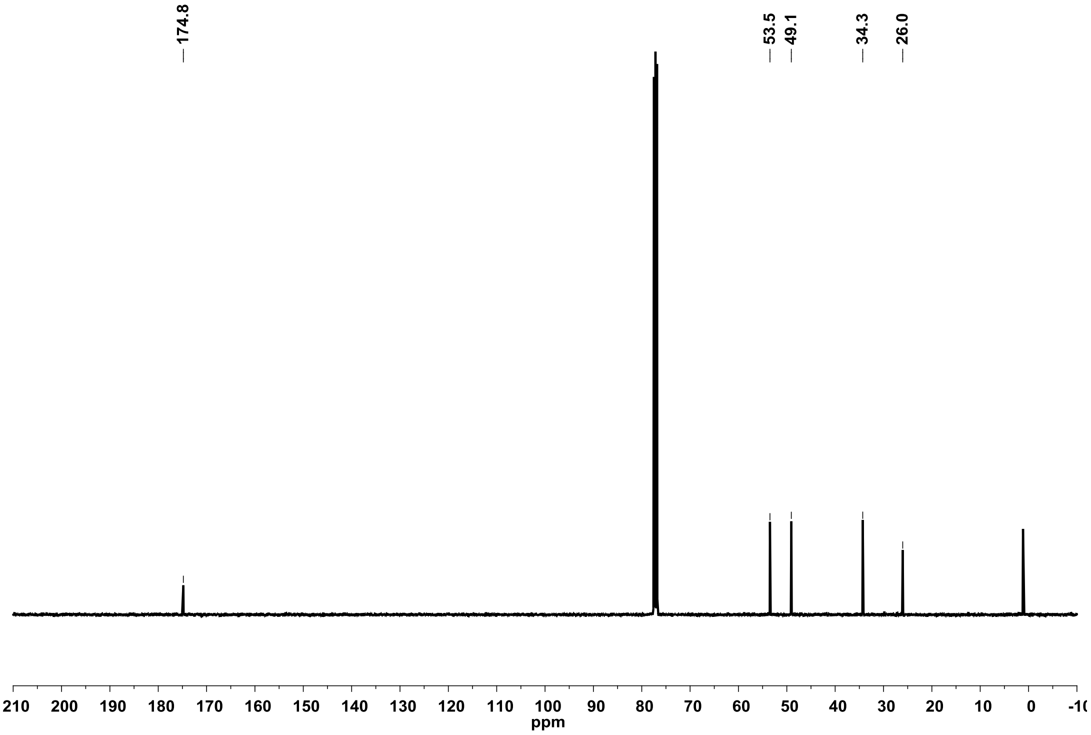

8.7 (*S*)-5-(2-Azidoethyl)-2,2,3-trimethylimidazolidin-4-one

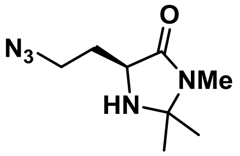

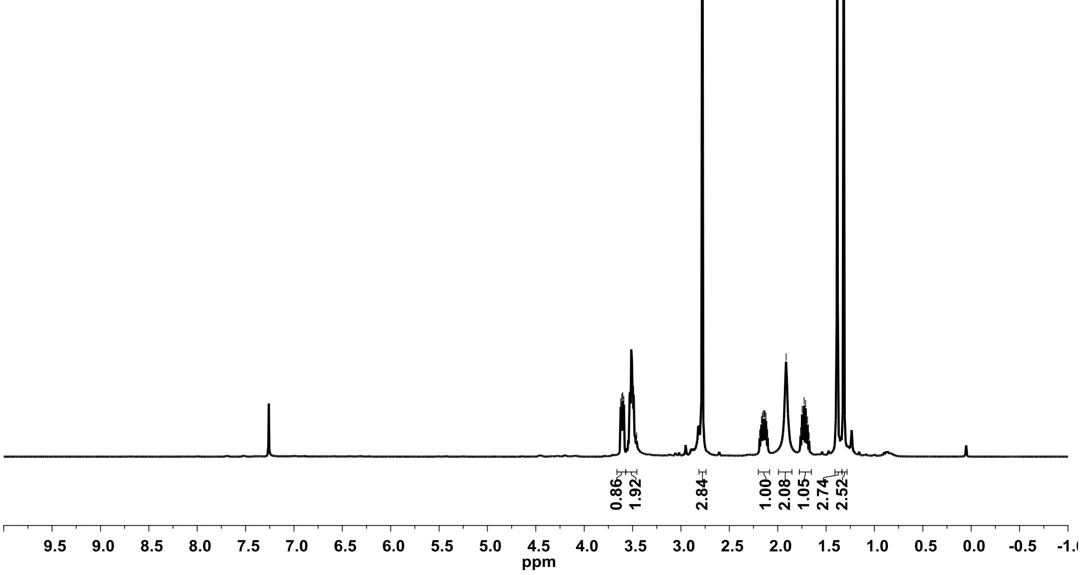

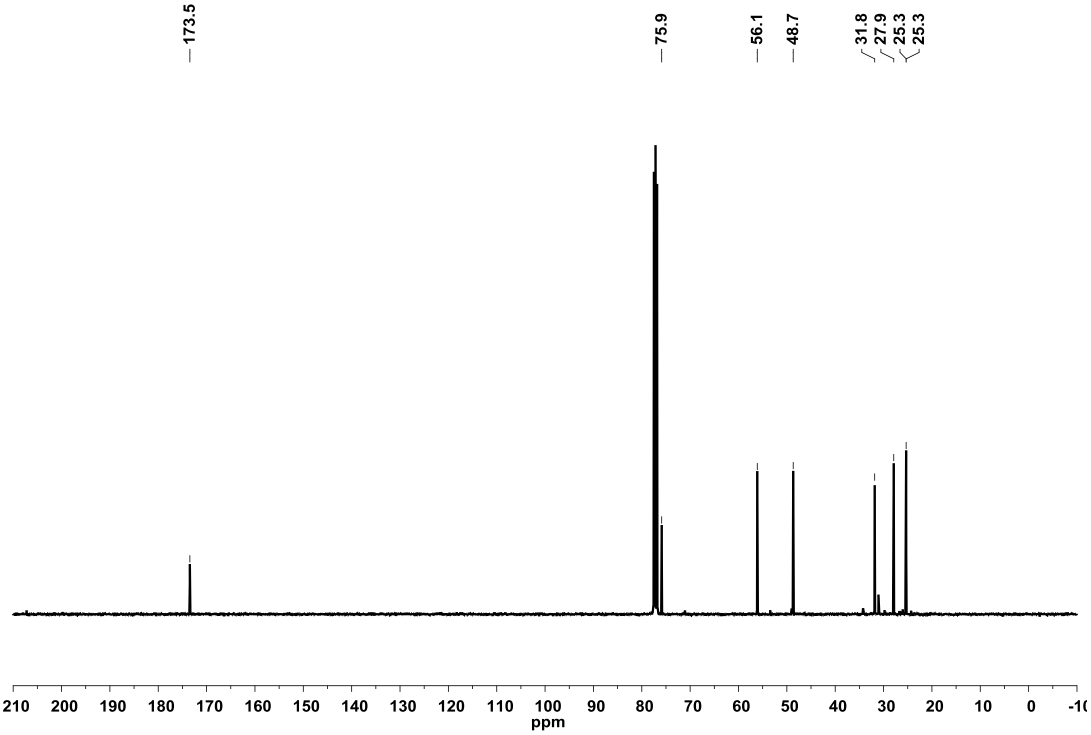

8.8 5-((3a*S*,4*S*,6a*R*)-2-oxohexahydro-1H-thieno[3,4-d]imidazol-4-yl)-*N*-((1-(2-((*S*)-1,2,2-trimethyl-5-oxoimidazolidin-4-yl)ethyl)-1H-1,2,3-triazol-4-yl)methyl)pentanamide 3

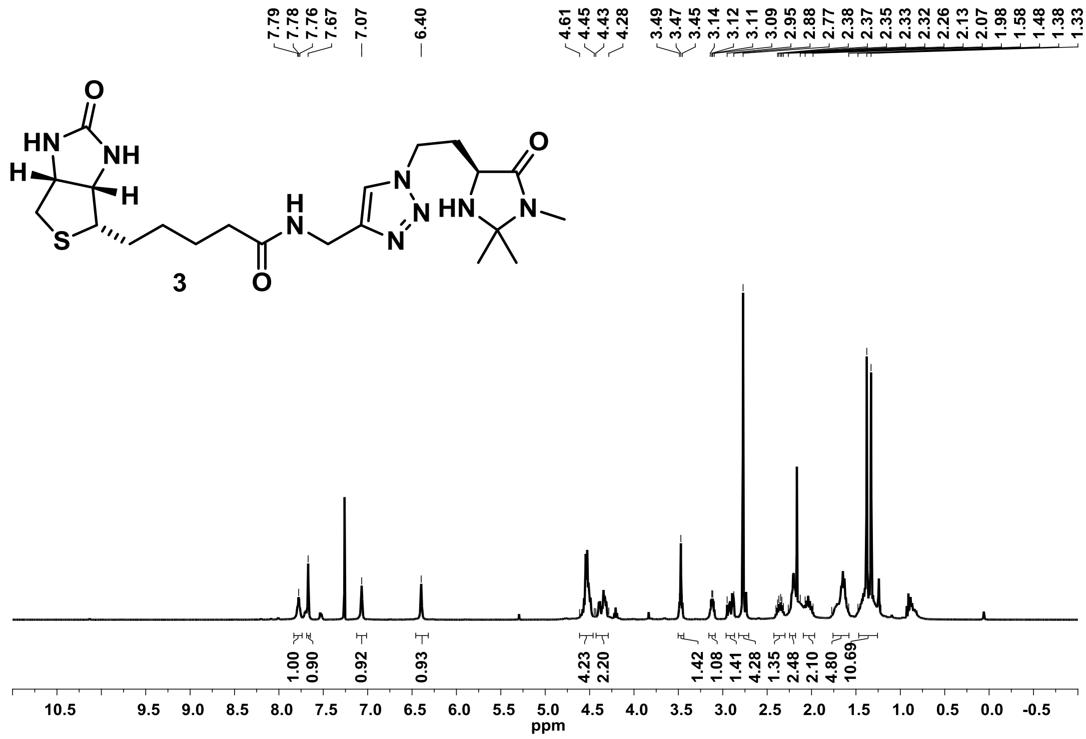

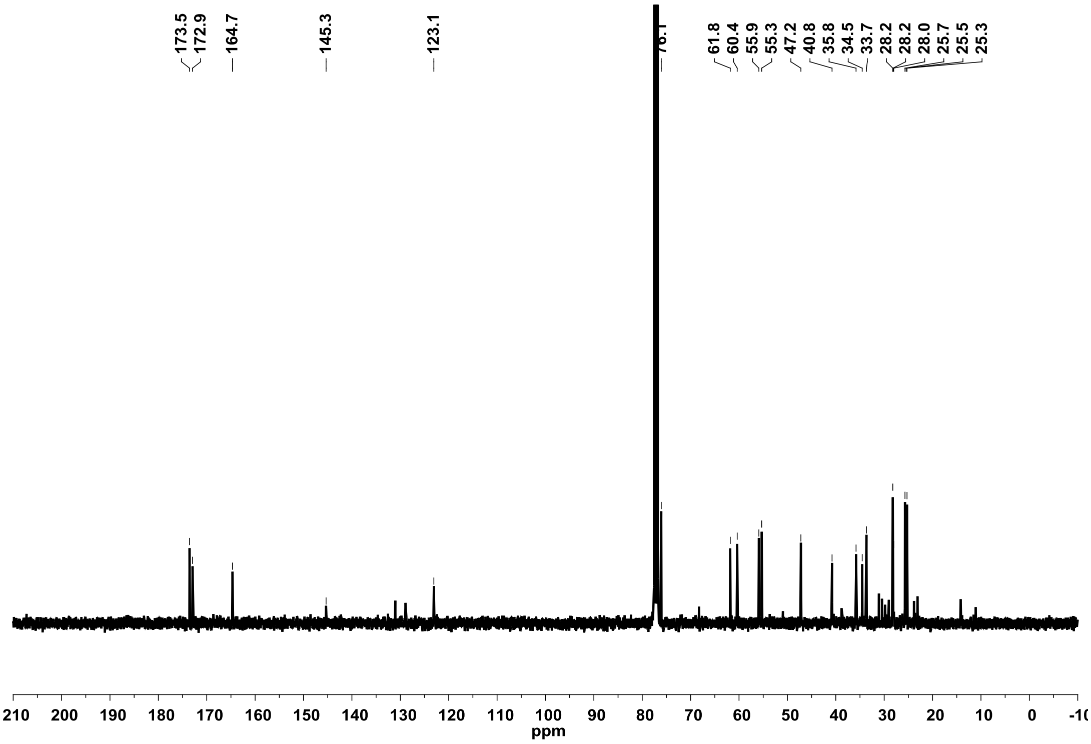

8.9 (5*S*)-5-(2-azidoethyl)-2-(*tert*-butyl)-3-methylimidazolidin-4-one

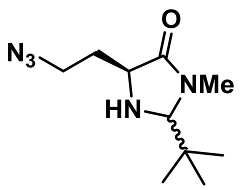

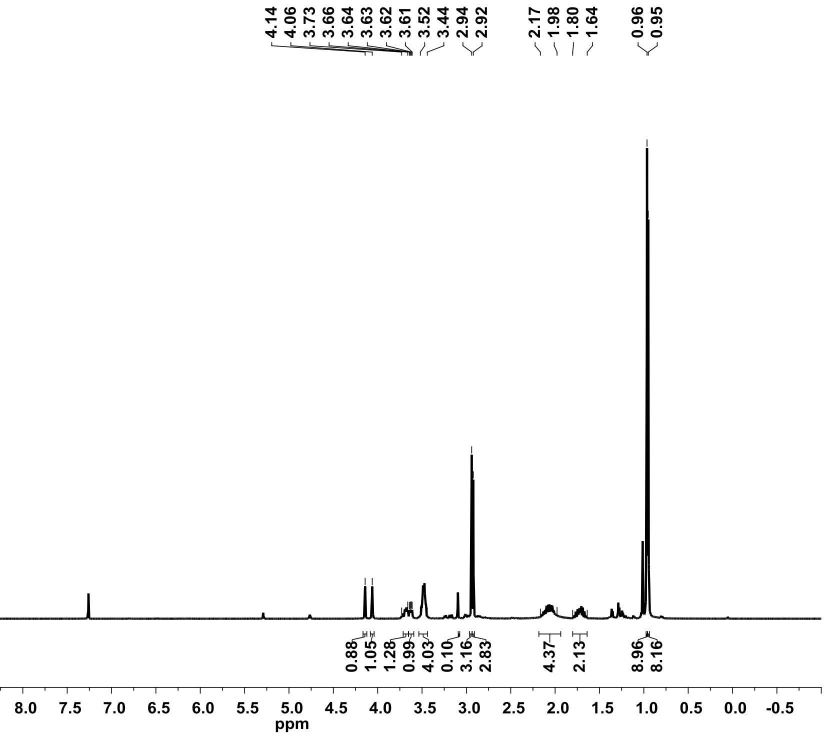

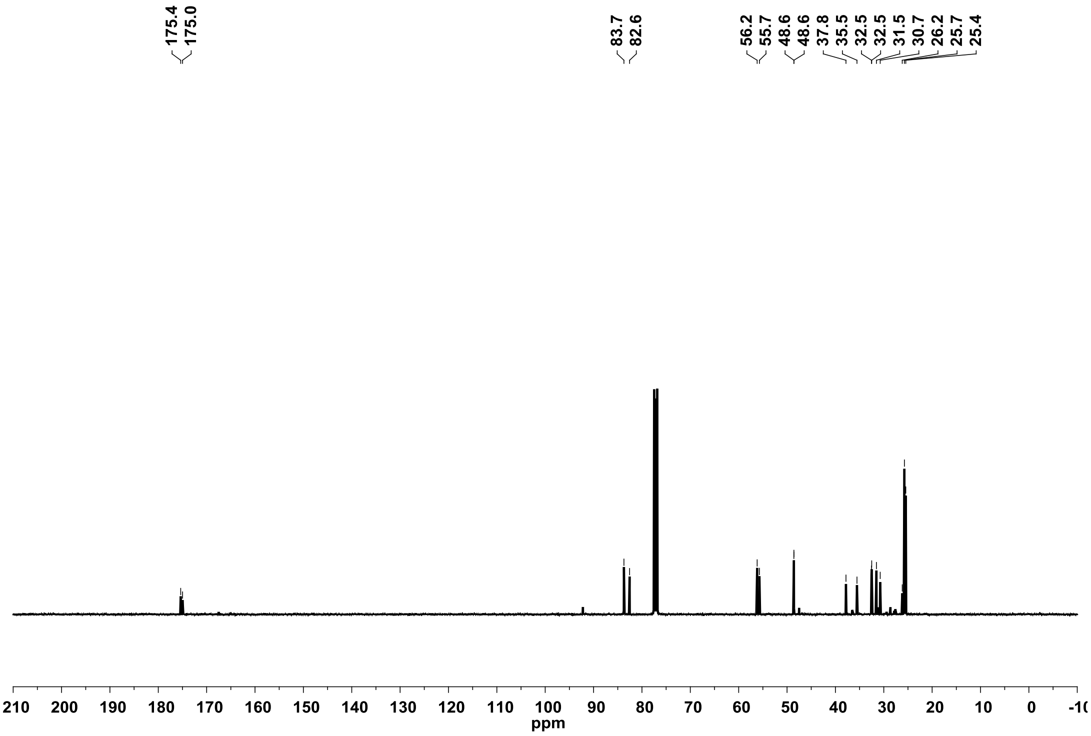

8.10 *N*-((1-(4-(((2*S*,4*S*)-2-(*tert*-butyl)-1-methyl-5-oxoimidazolidin-4-yl)methyl)phenyl)-1H-1,2,3-triazol-4-yl)methyl)-5-((3a*S*,4*S*,6a*R*)-2-oxohexahydro-1H-thieno[3,4-d]imidazol-4-yl)pentanamide 4

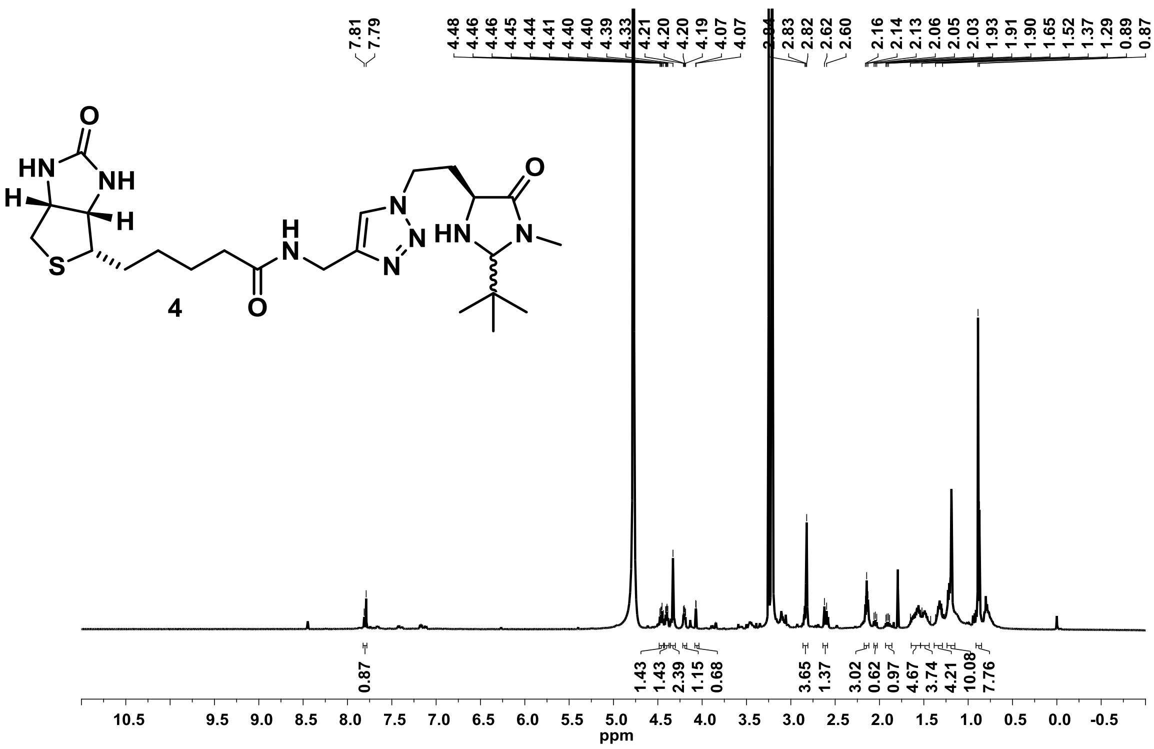

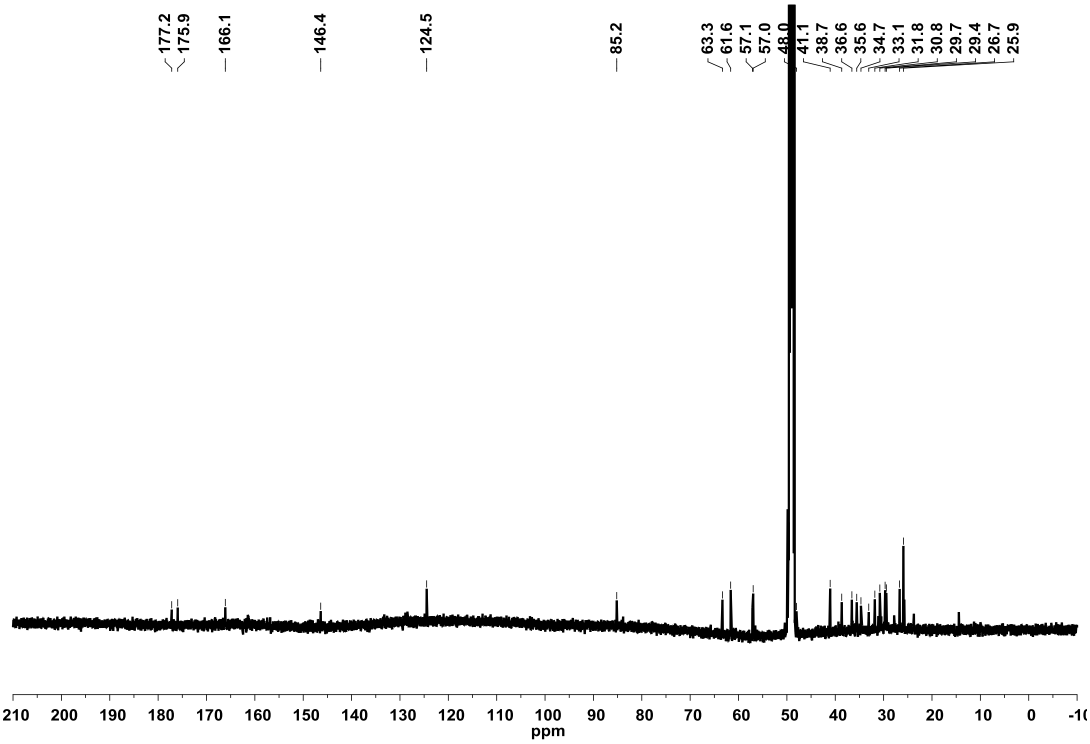

8.11 (2*S*,4*S*)-1-(*tert*-butoxycarbonyl)-4-(4-((5-((3a*S*,4*S*,6a*R*)-2-oxohexahydro-1H-thieno[3,4-d]imidazol-4-yl)pentanamido)methyl)-1H-1,2,3-triazol-1-yl)pyrrolidine-2-carboxylic acid

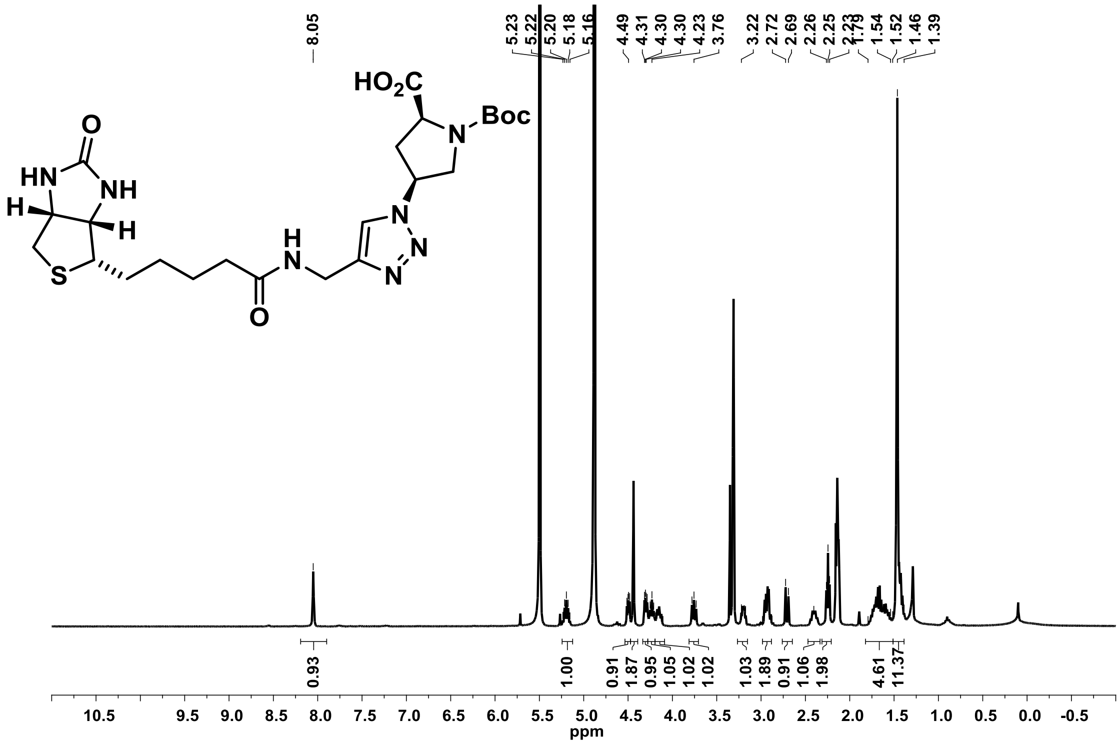

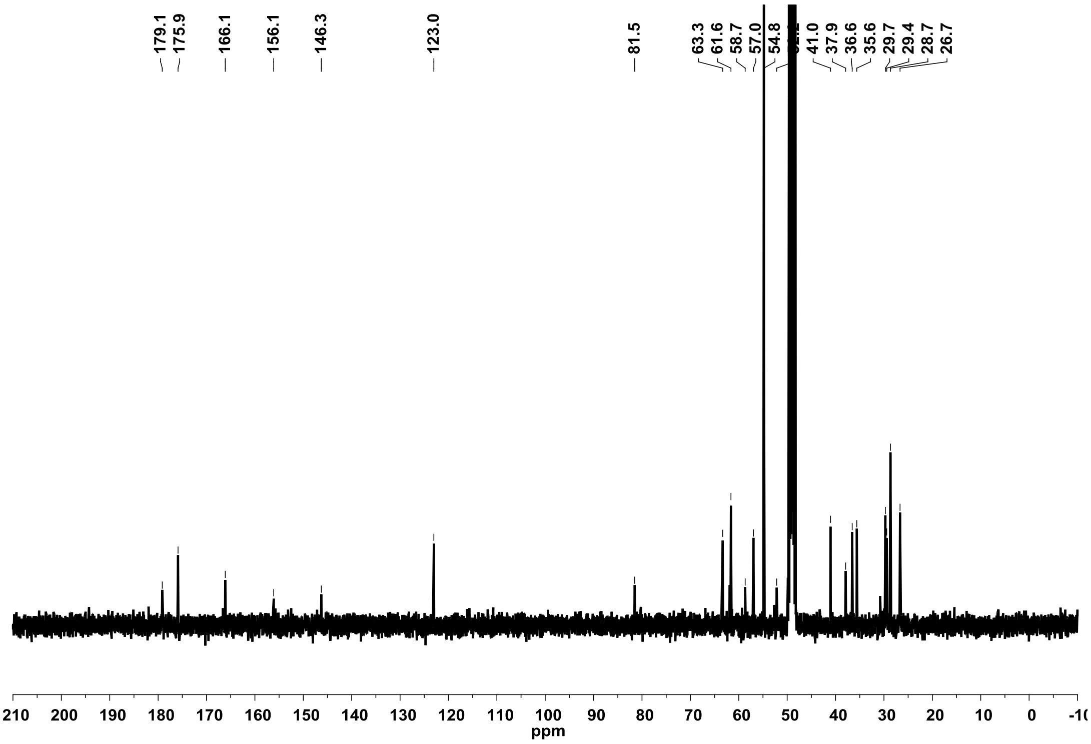

8.12 (2*S*,4*S*)-2-carboxy-4-(4-((5-((3a*S*,4*S*,6a*R*)-2-oxohexahydro-1H-thieno[3,4-d]imidazol-4-yl)pentanamido)methyl)-1H-1,2,3-triazol-1-yl)pyrrolidin-1-ium trifluoroacetate 5

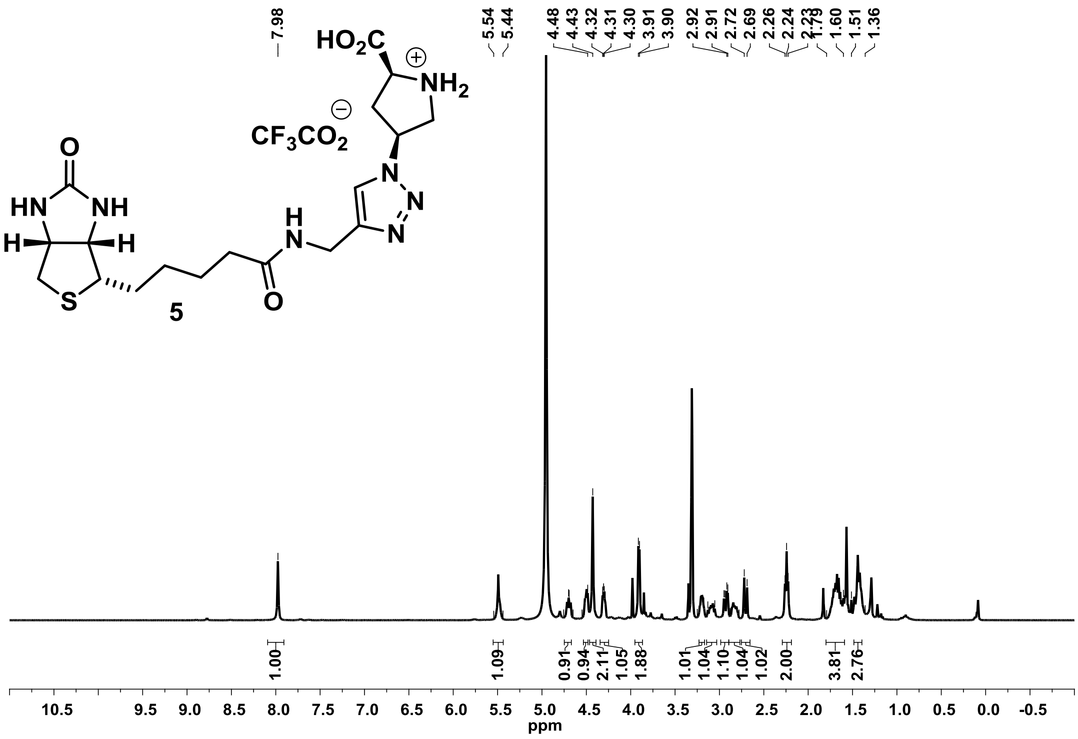

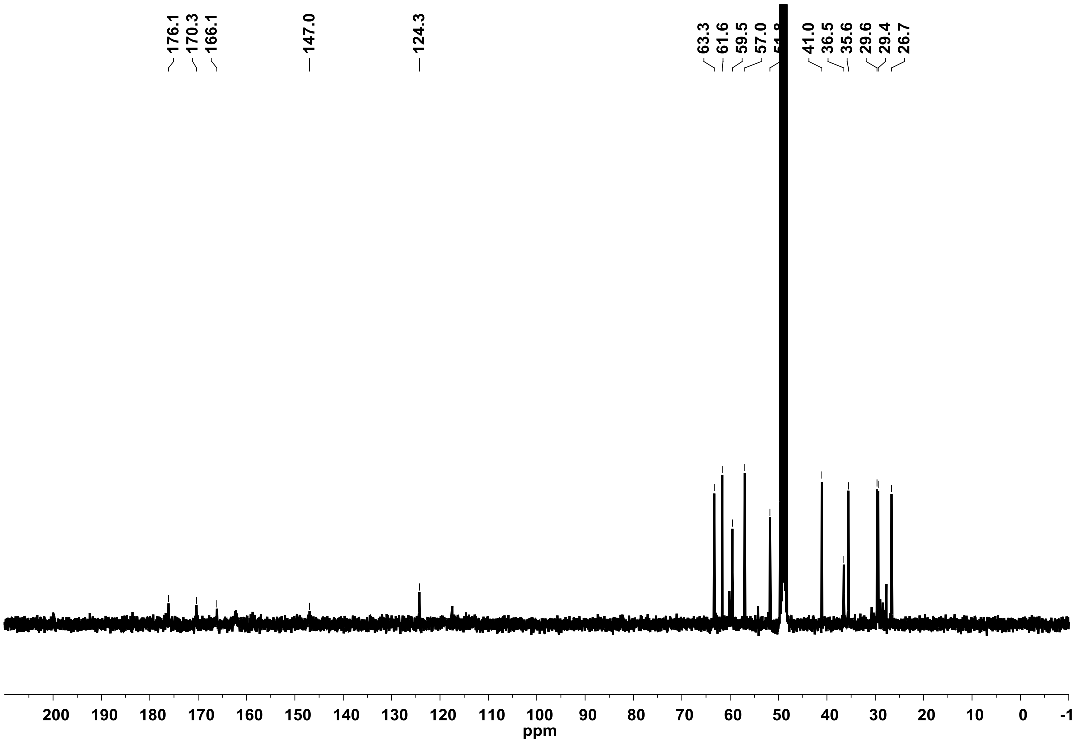

8.13 (2*S*,4*S*)-2-carboxy-4-(5-((3a*S*,4*S*,6a*R*)-2-oxohexahydro-1H-thieno[3,4-d]imidazol-4-yl)pentanamido)pyrrolidin-1-ium formate 6

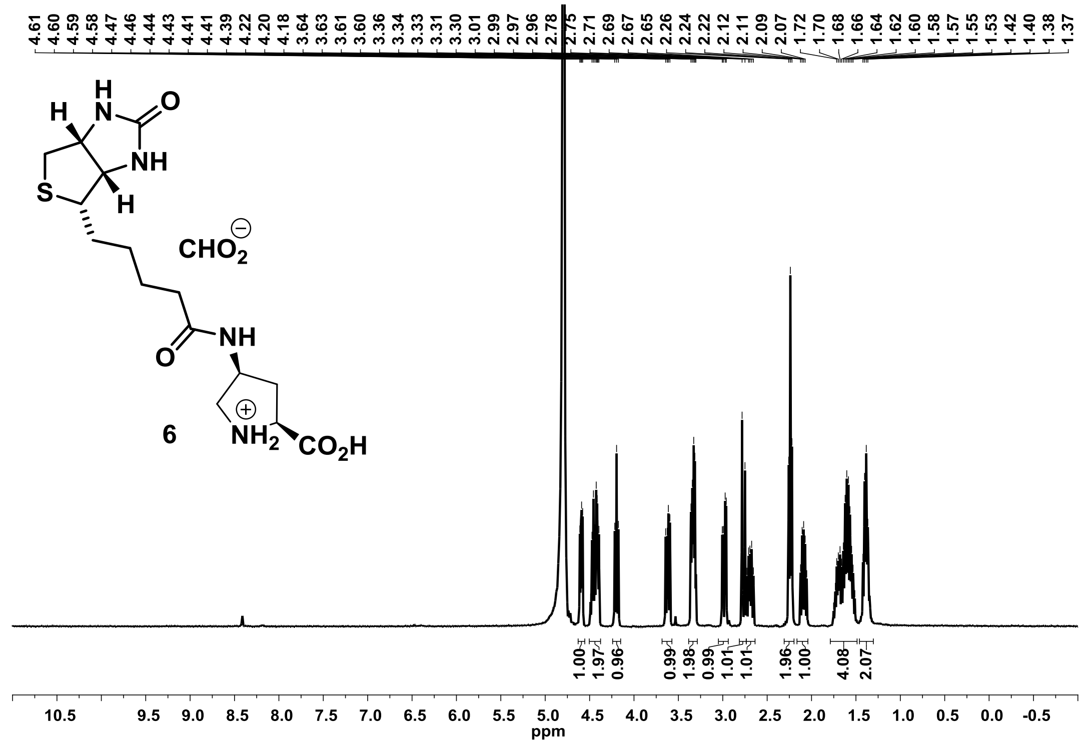
4.61 4.60 4.59 4.58 4.47 4.46 4.44 4.43 4.41 4.39 4.22 4.20 4.18 3.64 3.63 3.61 3.60 3.36 3.34 3.33 3.31 3.30 3.01 2.99 2.97 2.96 2.78 2.75 2.71 2.69 2.67 2.65 2.26 2.24 2.22 2.12 2.11 2.09 2.07 1.72 1.70 1.68 1.66 1.64 1.62 1.60 1.58 1.57 1.55 1.53 1.42 1.40 1.38 1.37
H
N
O
H
NH
S
H
CHO2
NH
O
6
NH2
CO2H
1.00 1.97 0.96 0.99 1.98 0.99 1.01 1.01 1.96 1.00 4.08 2.07
10.5 9.5 9.0 8.5 8.0 7.5 7.0 6.5 6.0 5.5 5.0 4.5 4.0 3.5 3.0 2.5 2.0 1.5 1.0 0.5 0.0 -0.5
ppm

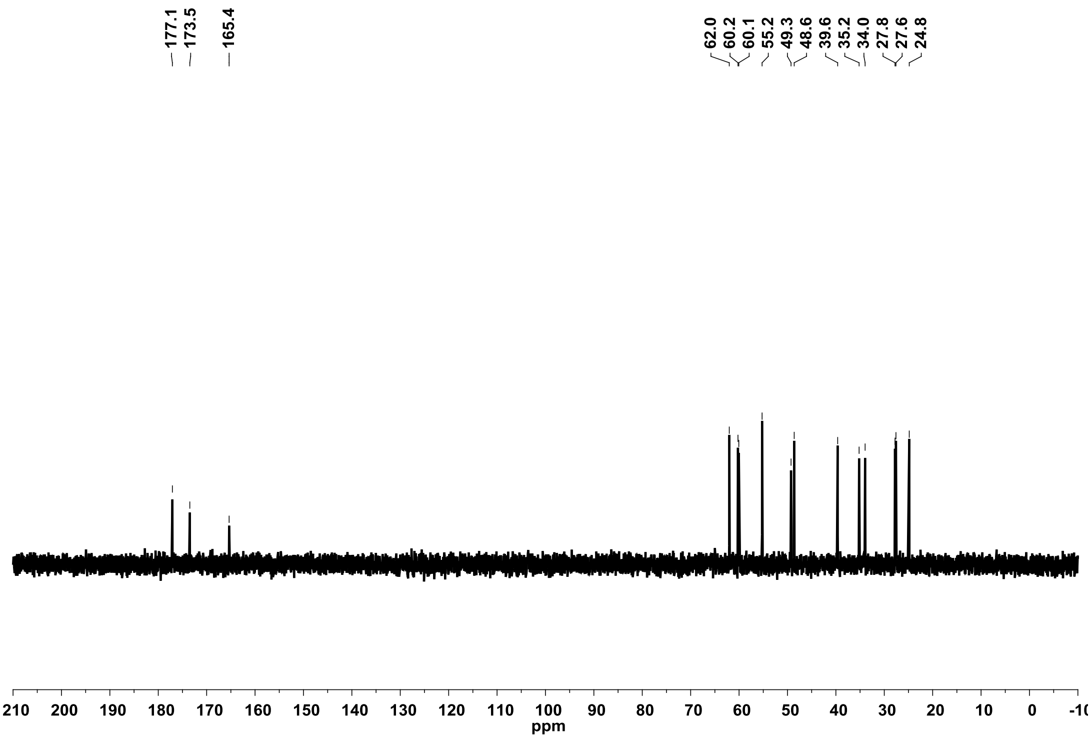
177.1 173.5 165.4
62.0 60.2 60.1 55.2 49.3 48.6 39.6 35.2 34.0 27.8 27.6 24.8
210 200 190 180 170 160 150 140 130 120 110 100 90 80 70 60 50 40 30 20 10 0
ppm

8.14 (*R*)-3-(5-((3a*S*,4*S*,6a*R*)-2-oxohexahydro-1H-thieno[3,4-d]imidazol-4-yl)pentanamido)pyrrolidin-1-ium formate 7

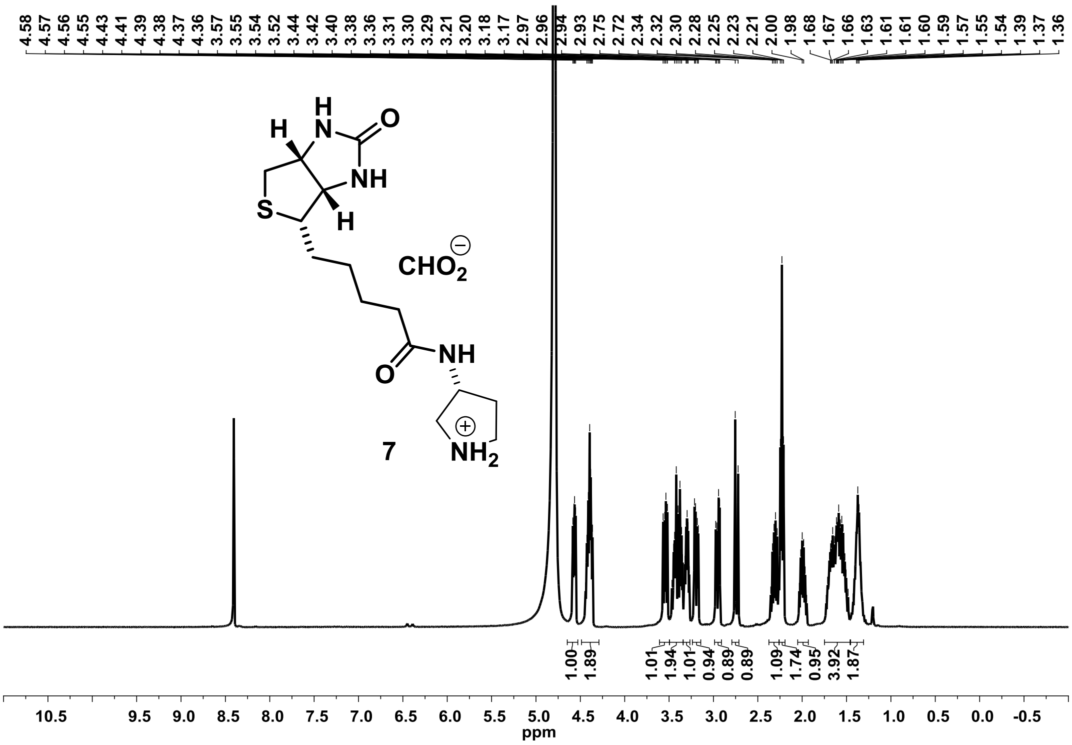

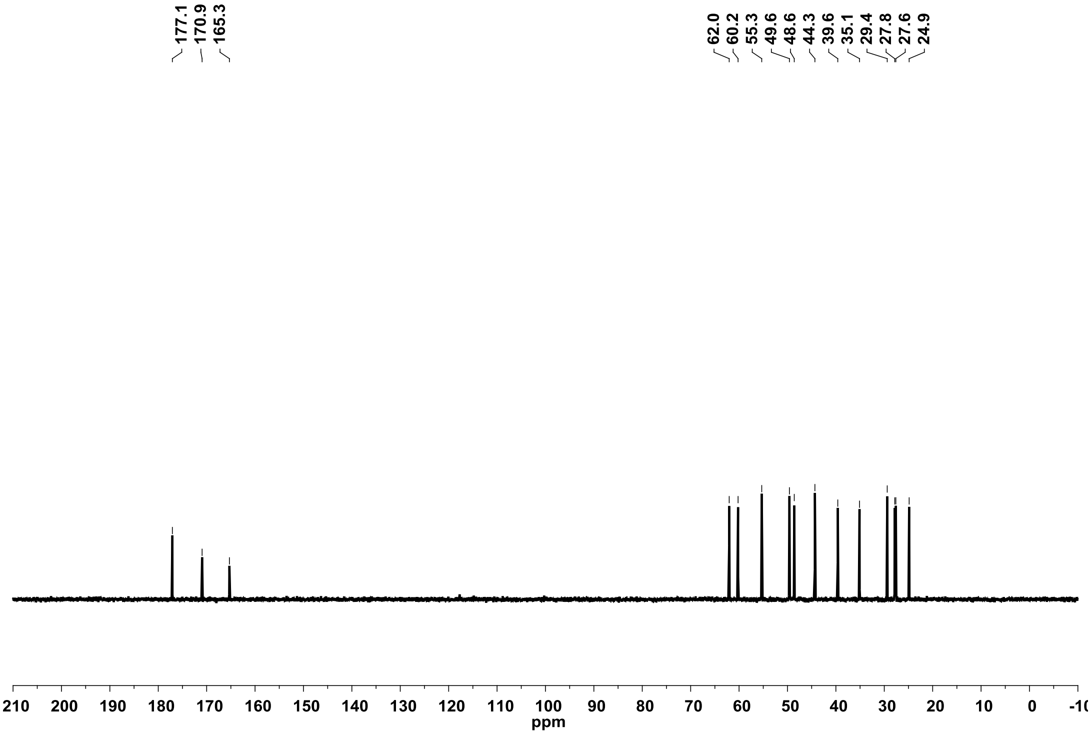

8.15 (*S*)-3-(5-((3a*S*,4*S*,6a*R*)-2-oxohexahydro-1H-thieno[3,4-d]imidazol-4-yl)pentanamido)pyrrolidin-1-ium formate 8

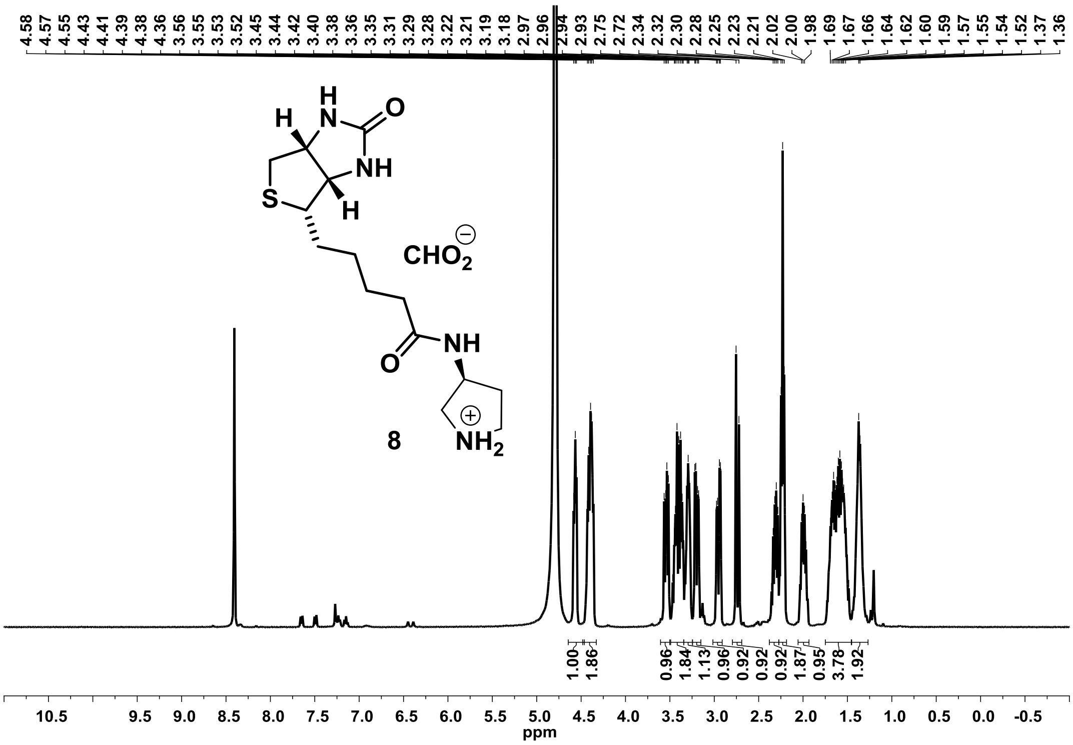

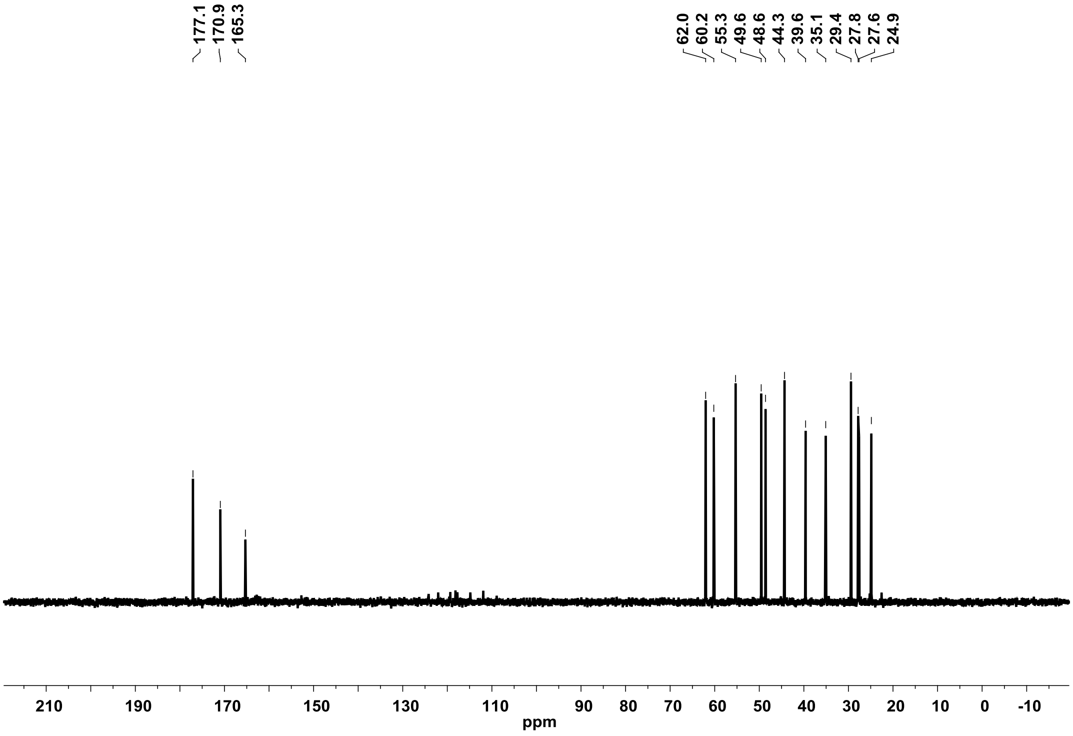

9 GCMS Traces of Catalyst Activity Screening

9.1 pH screening of catalyst 5

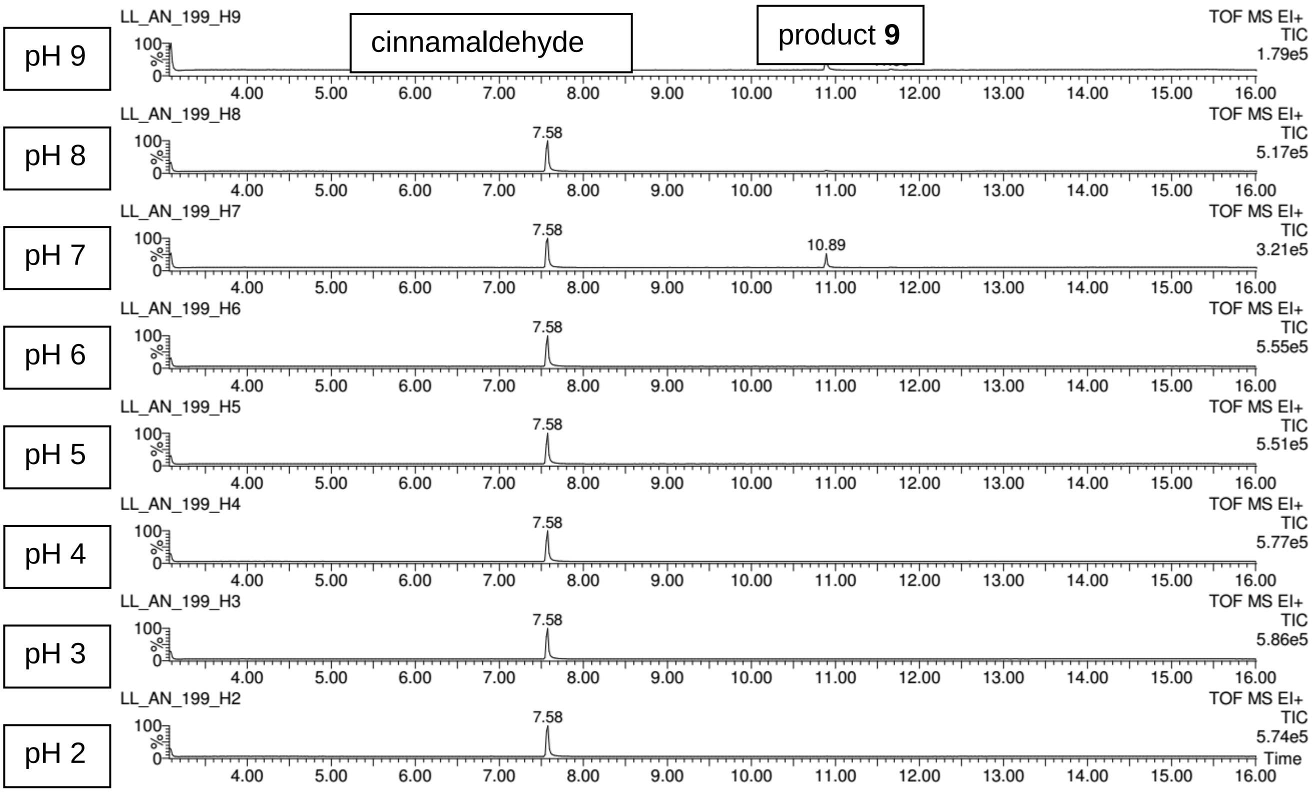

9.2 pH screening of catalyst 6

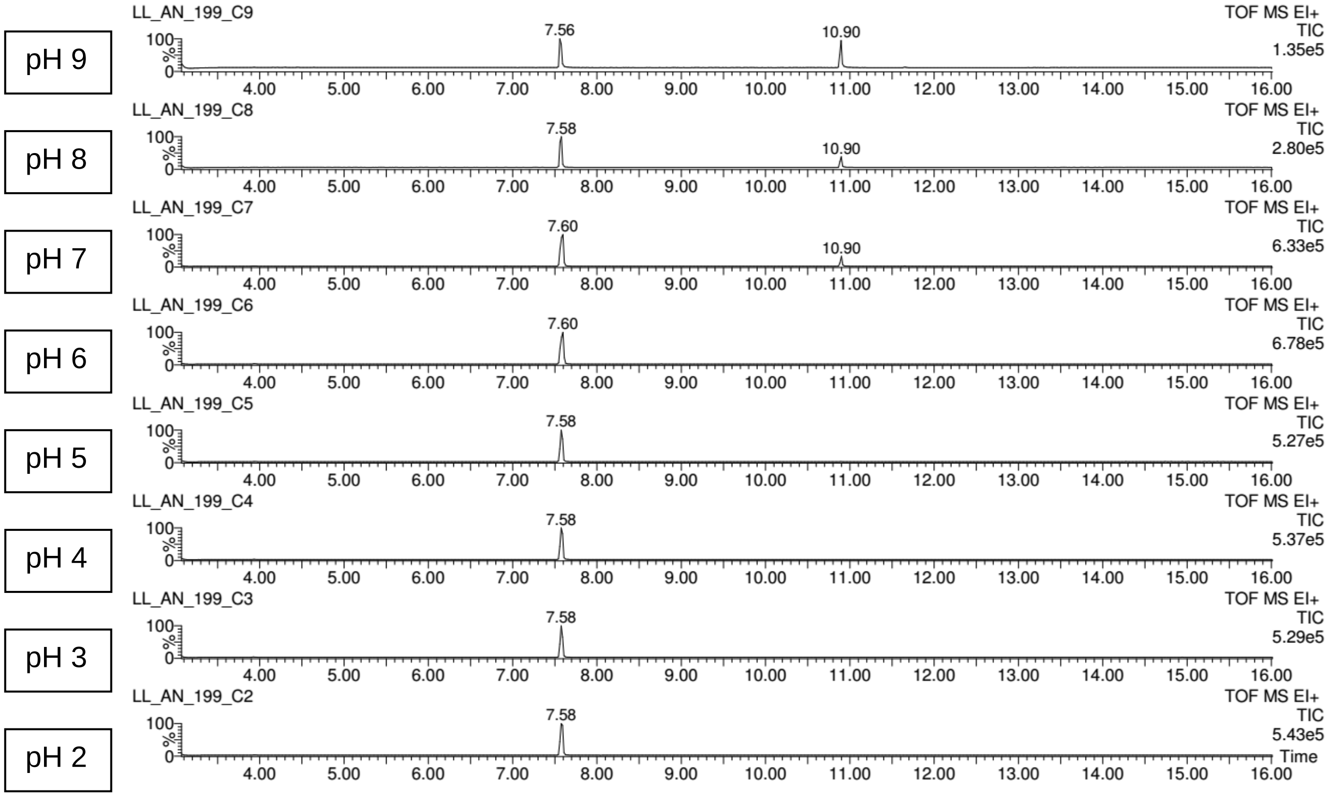

9.3 pH screening of catalyst 7

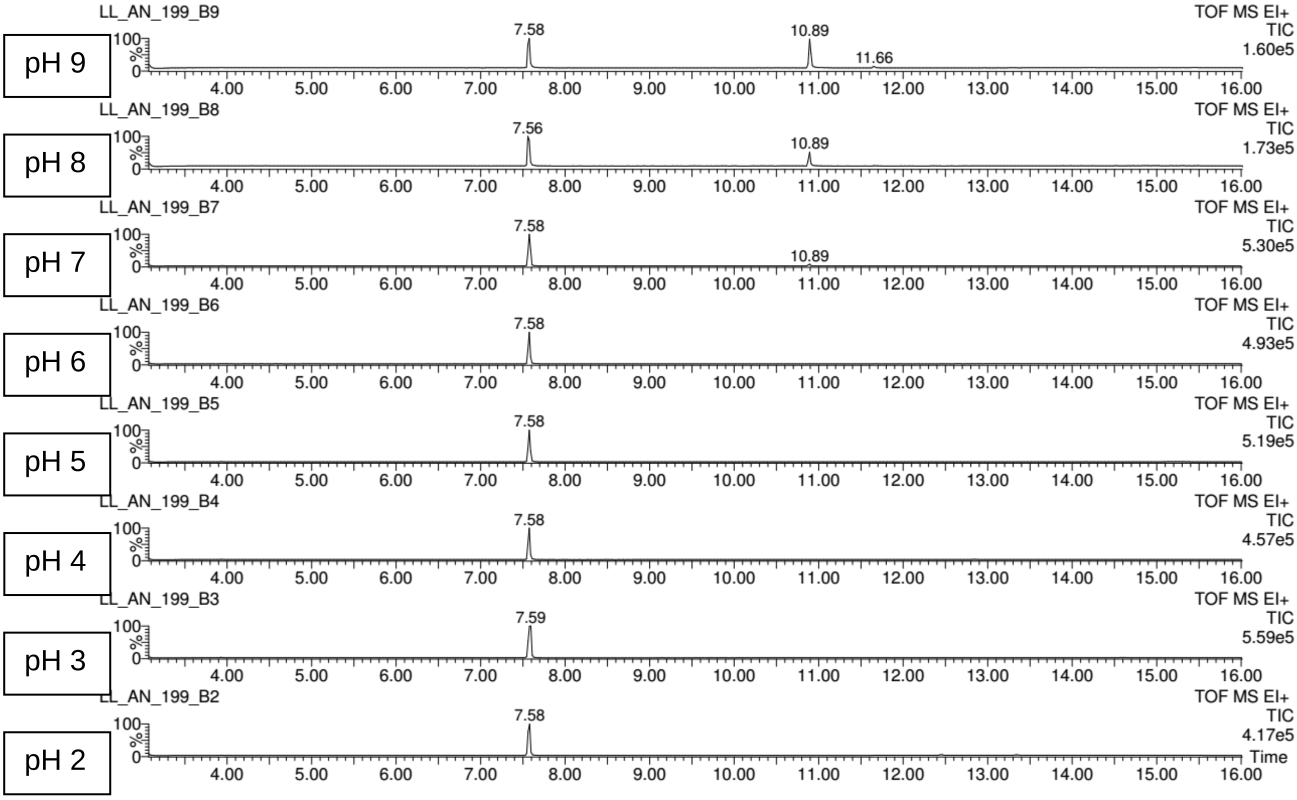

10 GCMS Traces of Hybrid Catalyst Activity Screening

10.1 Reactions at pH 7.0, 10 mM KP$_i$, 25 °C, 18 h

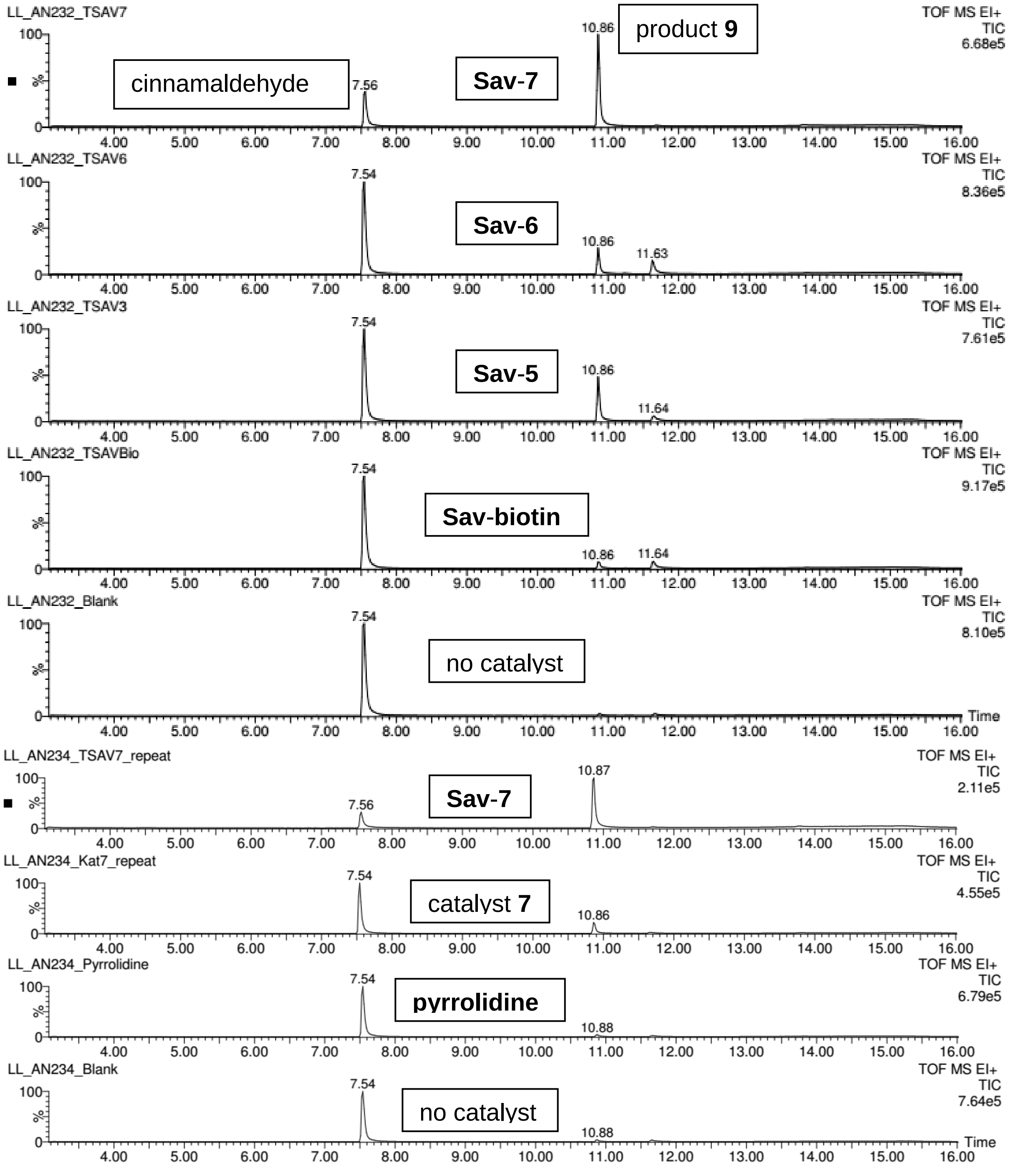

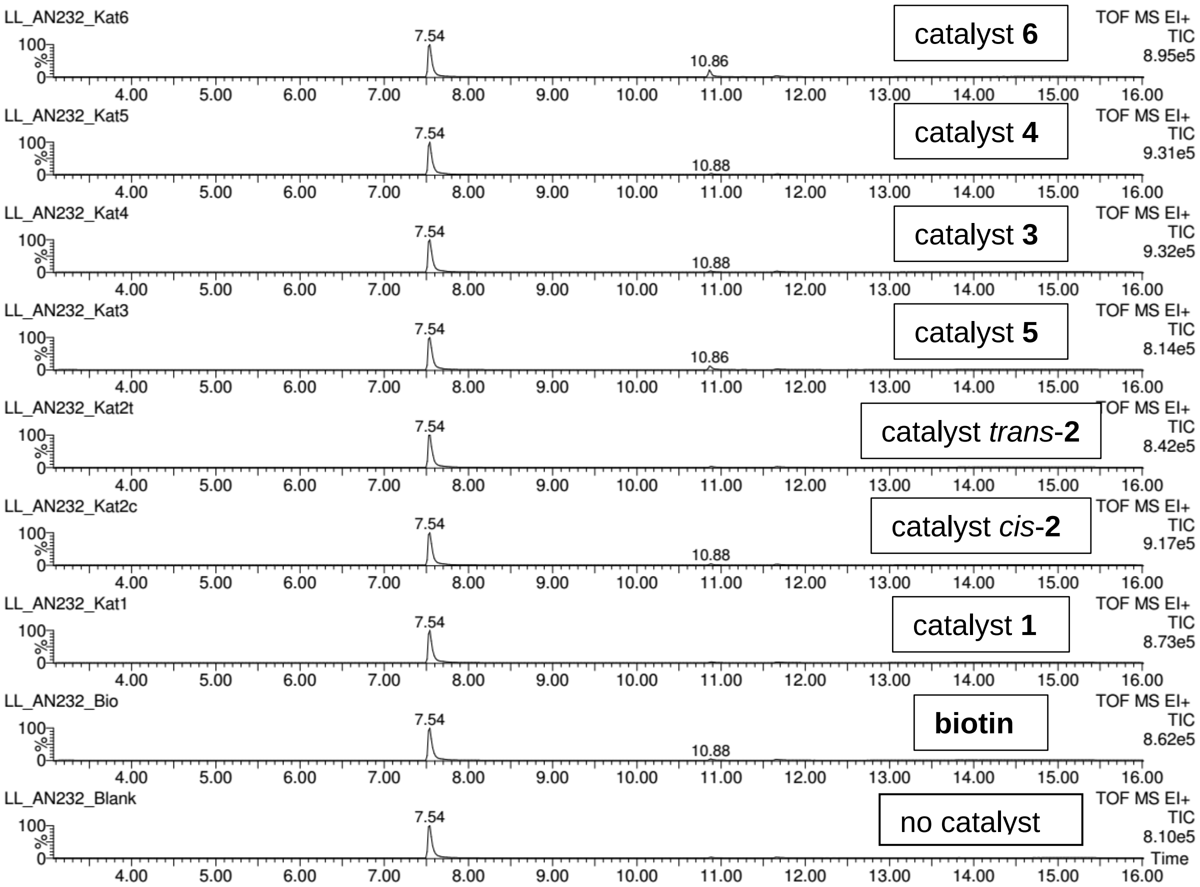
LL_AN232_Kat6
catalyst 6
TOF MS EI+
TIC
8.95e5
7.54
10.86
LL_AN232_Kat5
catalyst 4
9.31e5
10.88
LL_AN232_Kat4
catalyst 3
9.32e5
LL_AN232_Kat3
catalyst 5
8.14e5
LL_AN232_Kat2t
catalyst trans-2
8.42e5
LL_AN232_Kat2c
catalyst cis-2
9.17e5
LL_AN232_Kat1
catalyst 1
8.73e5
LL_AN232_Bio
biotin
8.62e5
LL_AN232_Blank
no catalyst
8.10e5
Time

10.2 Reactions at pH 8.0, 10 mm KP_i, 25 °C, 18 h

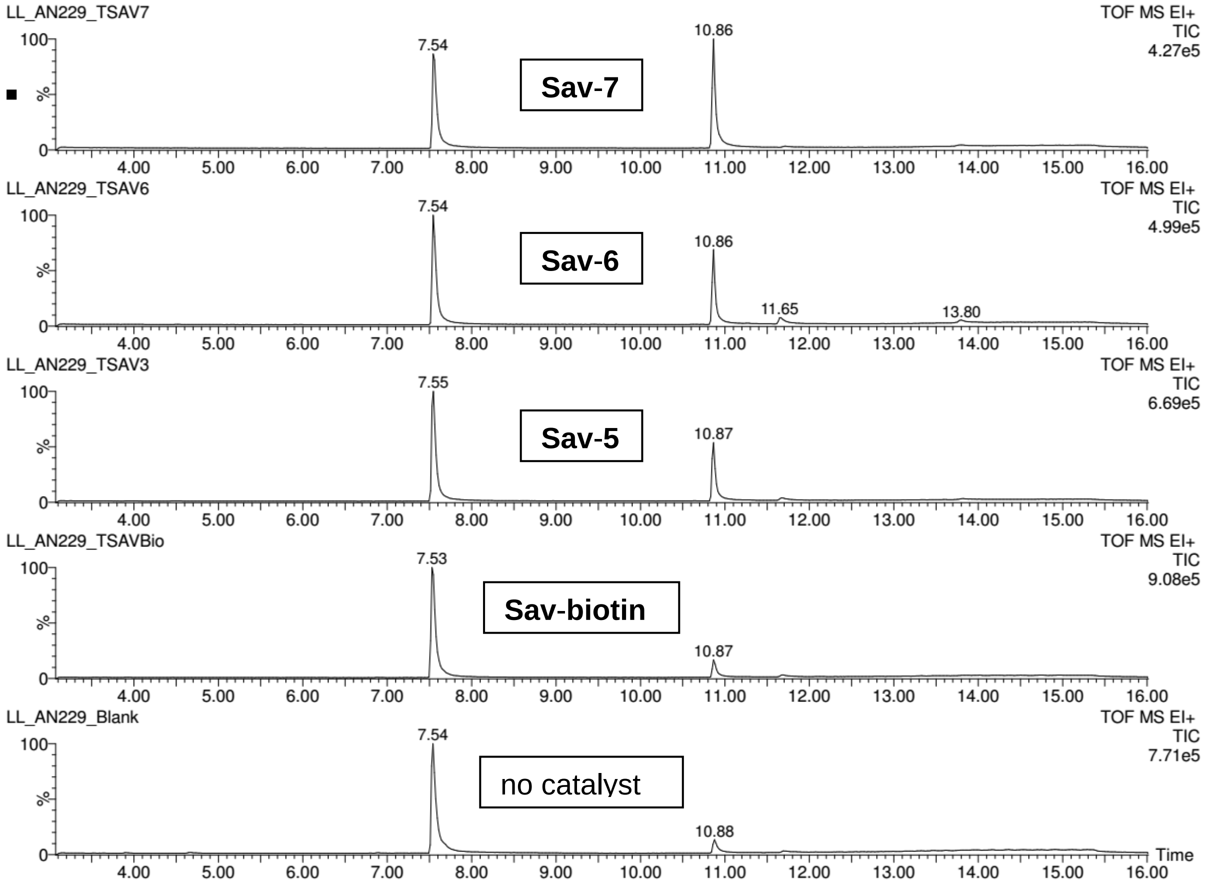

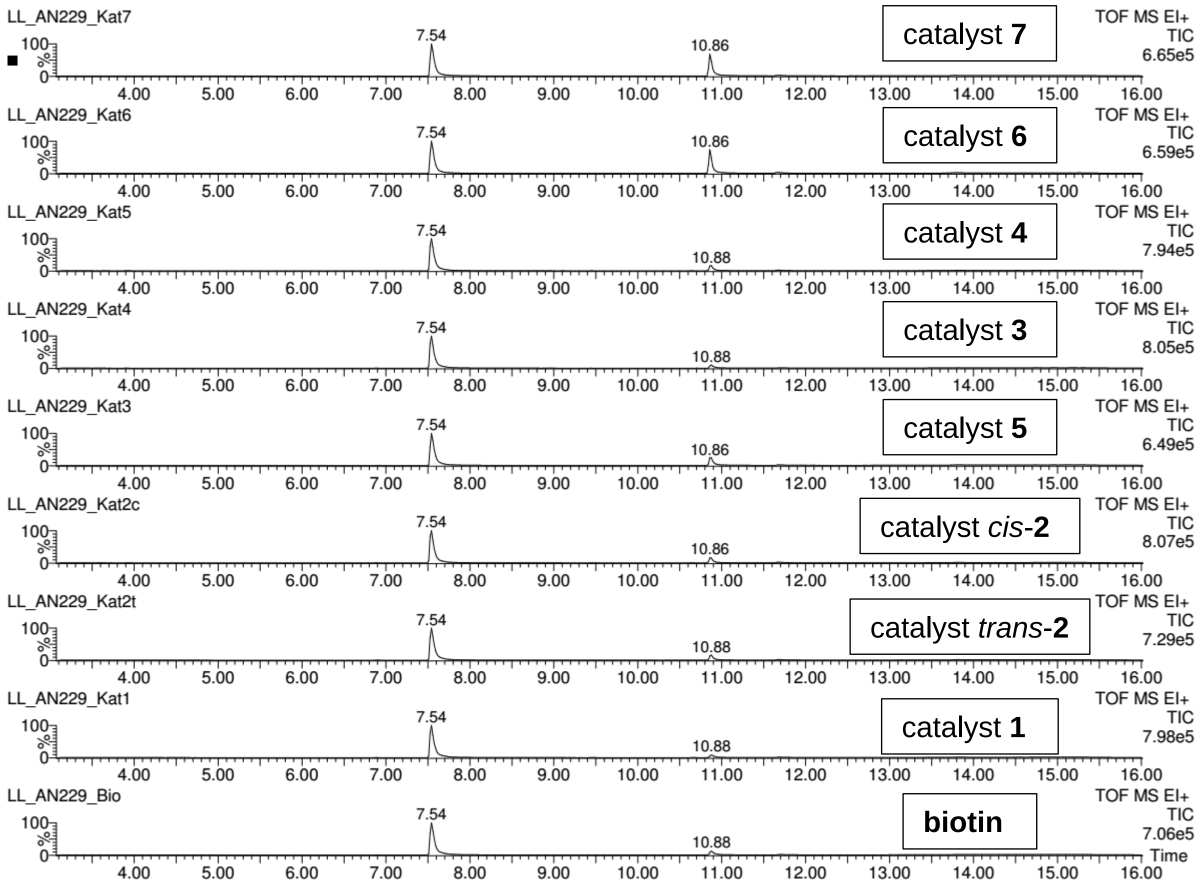
LL_AN229_Kat7
catalyst 7
TOF MS EI+
TIC
6.65e5
LL_AN229_Kat6
catalyst 6
6.59e5
LL_AN229_Kat5
catalyst 4
7.94e5
LL_AN229_Kat4
catalyst 3
8.05e5
LL_AN229_Kat3
catalyst 5
6.49e5
LL_AN229_Kat2c
catalyst cis-2
8.07e5
LL_AN229_Kat2t
catalyst trans-2
7.29e5
LL_AN229_Kat1
catalyst 1
7.98e5
LL_AN229_Bio
biotin
7.06e5
7.54
10.86
10.88
Time

10.3 Reactions at pH 9.0, 10 mM Tris, 25 °C, 18 h

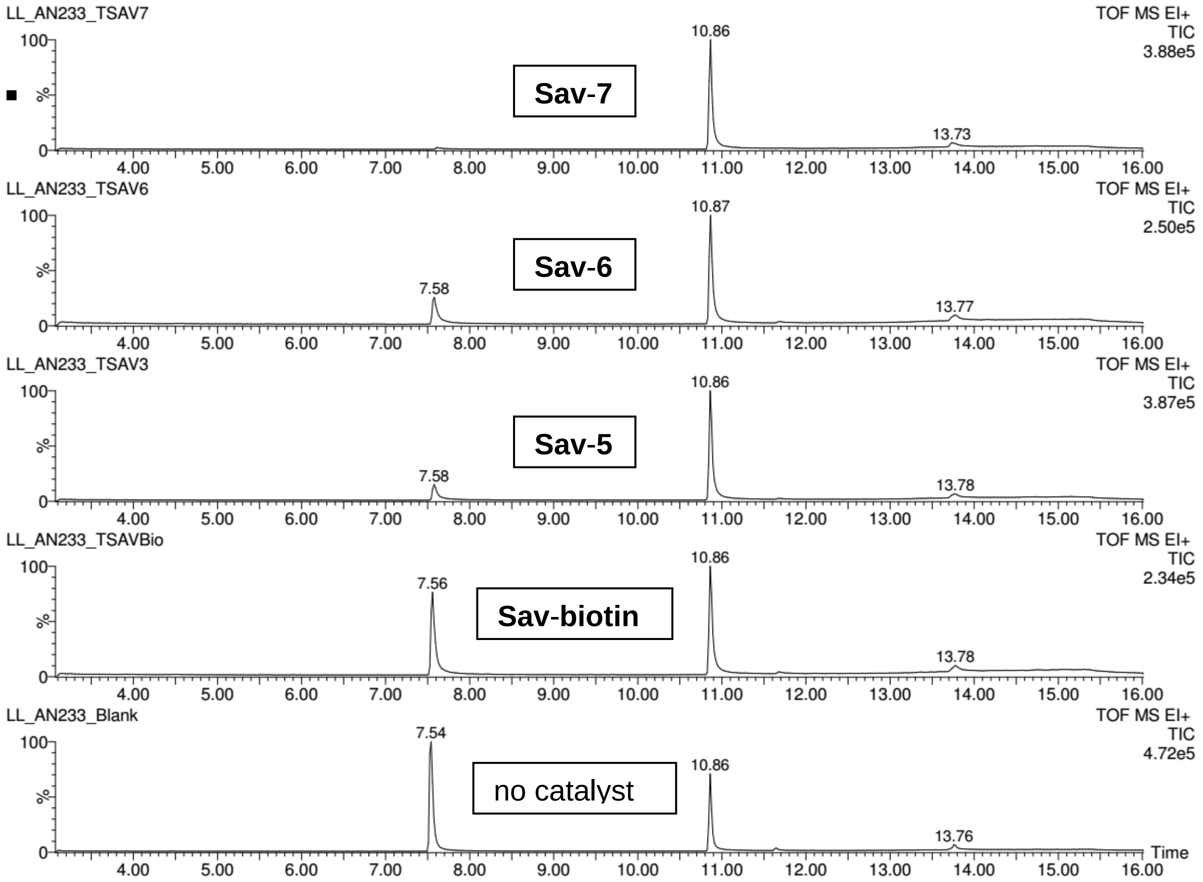

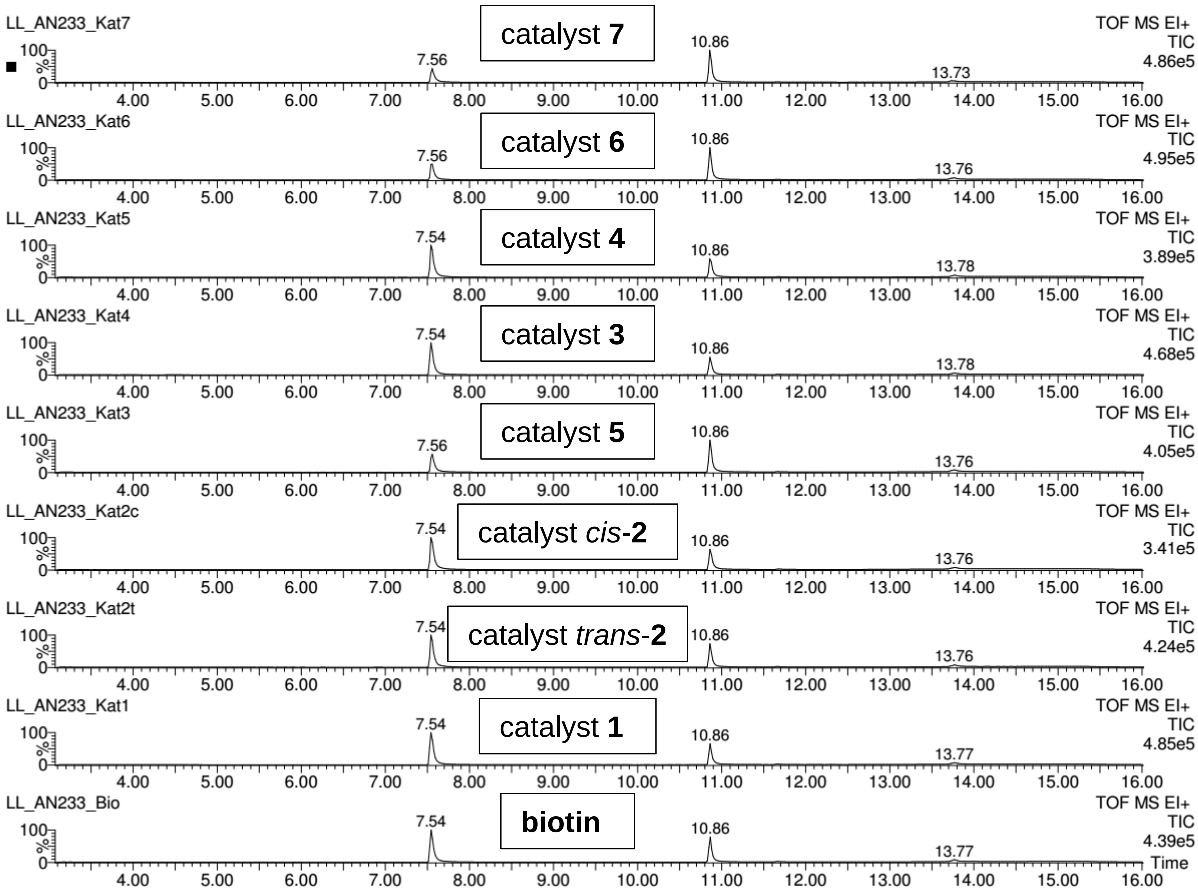
LL_AN233_Kat7
catalyst 7
TOF MS EI+
TIC
4.86e5
7.56
10.86
13.73
LL_AN233_Kat6
catalyst 6
TOF MS EI+
TIC
4.95e5
7.56
10.86
13.76
LL_AN233_Kat5
catalyst 4
TOF MS EI+
TIC
3.89e5
7.54
10.86
13.78
LL_AN233_Kat4
catalyst 3
TOF MS EI+
TIC
4.68e5
7.54
10.86
13.78
LL_AN233_Kat3
catalyst 5
TOF MS EI+
TIC
4.05e5
7.56
10.86
13.76
LL_AN233_Kat2c
catalyst cis-2
TOF MS EI+
TIC
3.41e5
7.54
10.86
13.76
LL_AN233_Kat2t
catalyst trans-2
TOF MS EI+
TIC
4.24e5
7.54
10.86
13.76
LL_AN233_Kat1
catalyst 1
TOF MS EI+
TIC
4.85e5
7.54
10.86
13.77
LL_AN233_Bio
biotin
TOF MS EI+
TIC
4.39e5
7.54
10.86
13.77
100
%
0
4.00 5.00 6.00 7.00 8.00 9.00 10.00 11.00 12.00 13.00 14.00 15.00 16.00
Time

11 Table of Optimized Catalytic Runs for Yield Determination

11.1 Catalytic Runs – KP_i Buffer

A = aldehyde protons, normalized to 1, S = *ortho* protons of nitrobenzene standard, DB = double bond protons, P = product CH_2 protons, see 4.2.

	pH 7 42 h				`pH 8 42 h			
	Integrals				Integrals			
**Catalyst**	**A**	**S**	**DB**	**P**	**A**	**S**	**DB**	**P**
**None**	1.00	1.59	1.01	0.04	1.00	1.84	1.05	0.08
**Pyrrolidine**	1.00	1.79	1.00	0.05	1.00	2.39	0.99	0.08
**L-Proline**	1.00	1.40	0.98	0.05	1.00	2.32	0.97	0.11
**Biotin**	1.00	1.56	1.01	0.04	1.00	1.95	1.02	0.09
**5**	1.00	1.69	1.00	0.10	1.00	3.02	0.96	0.13
**6**	1.00	1.48	0.97	0.11	1.00	2.50	0.96	0.17
**7**	1.00	1.98	0.93	0.14	1.00	2.71	0.95	0.22
**8**	1.00	1.70	0.97	0.14	1.00	2.74	0.97	0.18
**Sav-Bio**	1.00	1.62	1.00	0.06	1.00	2.17	0.97	0.12
**Sav-5**	1.00	2.14	0.99	0.15	1.00	3.03	0.95	0.15
**Sav-6**	1.00	1.66	0.96	0.08	1.00	3.16	0.95	0.16
**Sav-7**	1.00	3.15	0.68	0.63	1.00	4.94	0.86	0.40
**Sav-8**	1.00	1.77	0.85	0.27	1.00	3.51	0.93	0.20

12 Chiral HPLC Data of Micro-Scale Reactions

12.1 Cinnamaldehyde Runs under Different Conditions

Racemate 11a

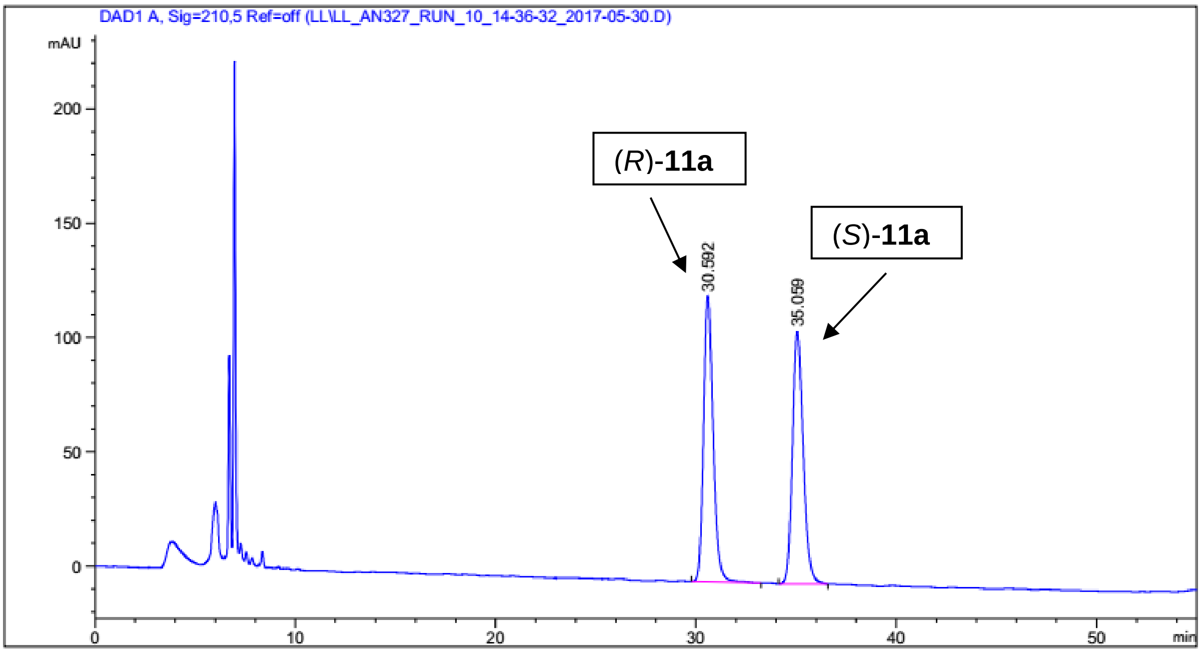

Signal 1: DAD1 A, Sig=210,5 Ref=off

Peak #	RetTime [min]	Type	Width [min]	Area [mAU*s]	Height [mAU]	Area %
1	30.592	BB	0.5127	4166.16895	125.05280	50.3085
2	35.059	BB	0.5760	4115.06738	110.10015	49.6915
Totals :				8281.23633	235.15295	

The peaks were assigned based on the known selectivity with catalyst **L-14**.[3-4]

Product 11a with Catalyst 7, Pure Buffer

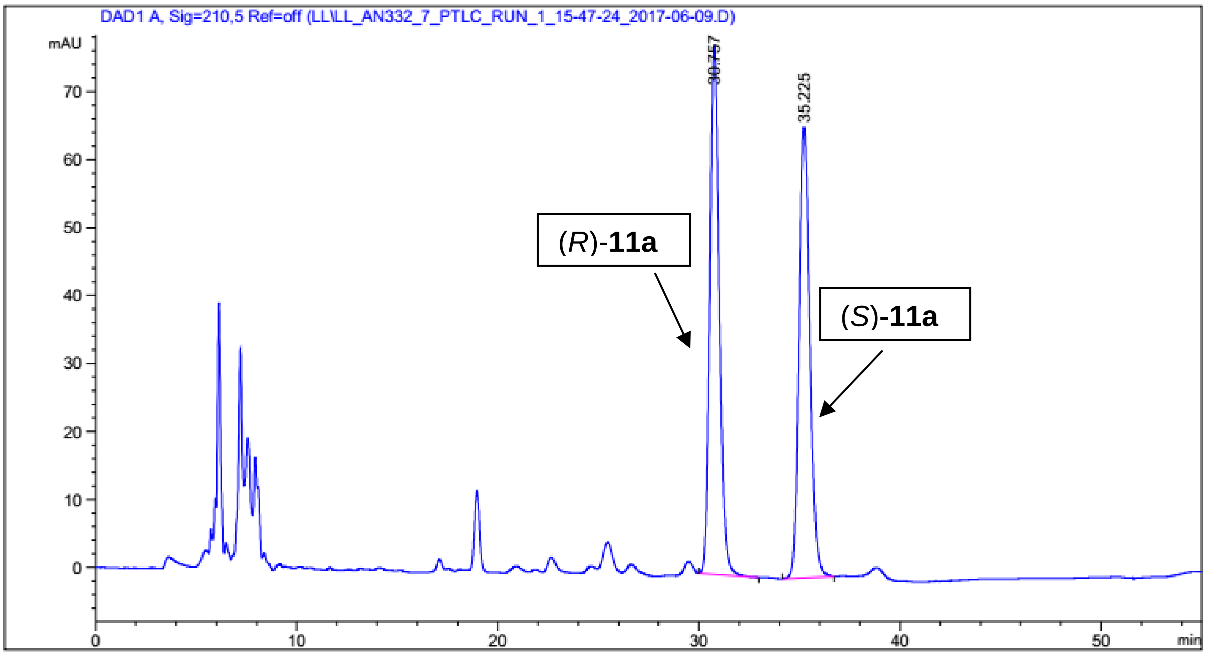

Signal 1: DAD1 A, Sig=210,5 Ref=off

Peak #	RetTime [min]	Type	Width [min]	Area [mAU*s]	Height [mAU]	Area %
1	30.757	BB	0.5105	2565.38477	77.84309	50.8575
2	35.225	BB	0.5743	2478.87720	66.29137	49.1425

Totals : 5044.26196 144.13446

Product 11a with Catalyst 8

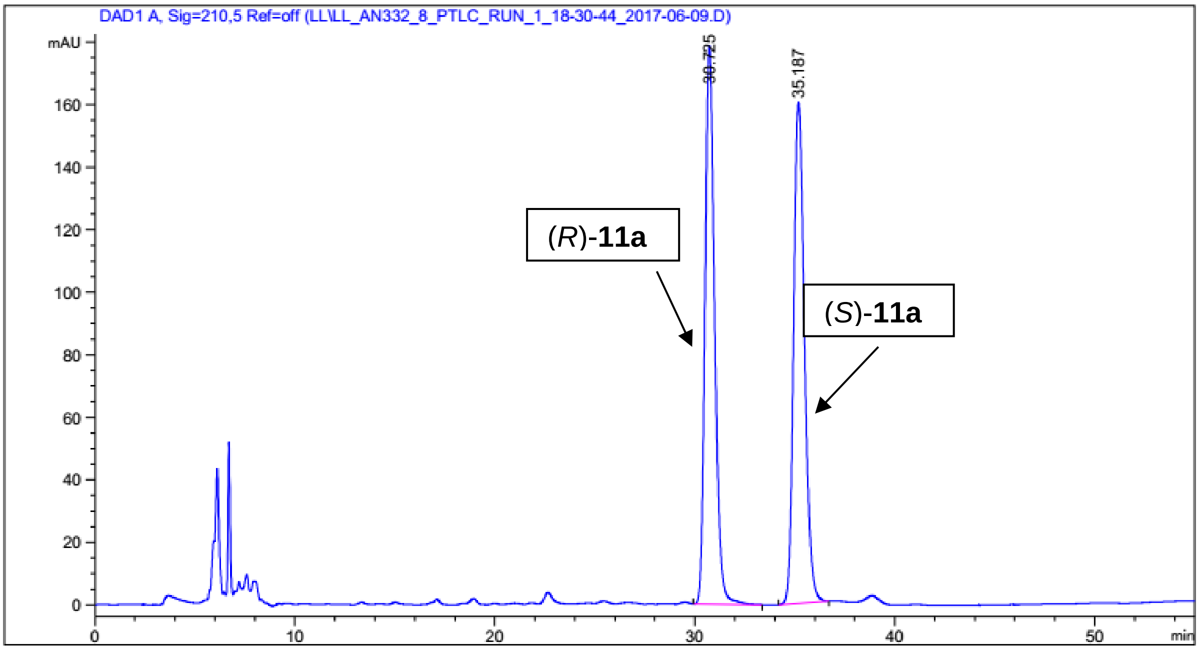

Signal 1: DAD1 A, Sig=210,5 Ref=off

Peak #	RetTime [min]	Type	Width [min]	Area [mAU*s]	Height [mAU]	Area %
1	30.725	BB	0.5149	5961.86670	178.85182	49.8100
2	35.187	BB	0.5797	6007.34912	160.14040	50.1900

Totals : 1.19692e4 338.99222

Product 11a with Catalyst Sav-7

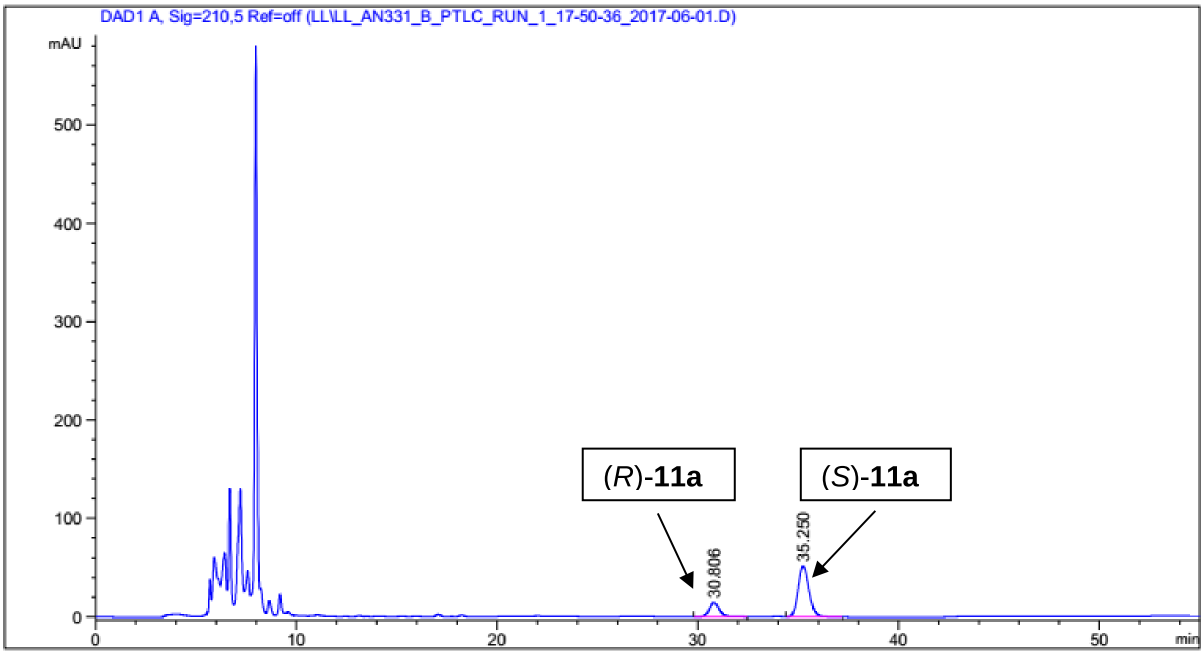

Signal 1: DAD1 A, Sig=210,5 Ref=off

Peak #	RetTime [min]	Type	Width [min]	Area [mAU*s]	Height [mAU]	Area %
1	30.806	BB	0.5231	497.39508	14.46612	20.4615
2	35.250	BB	0.5808	1933.48206	51.64398	79.5385

Totals :				2430.87714	66.11010	

Product 11a with Catalyst Sav-8

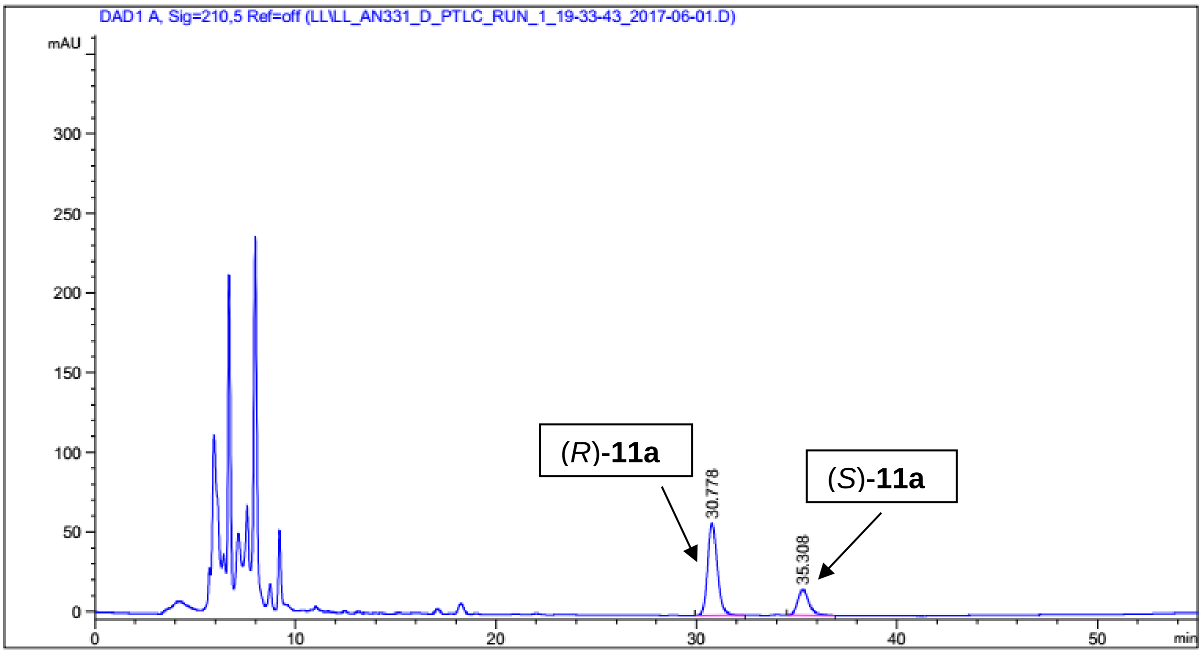

Signal 1: DAD1 A, Sig=210,5 Ref=off

Peak #	RetTime [min]	Type	Width [min]	Area [mAU*s]	Height [mAU]	Area %
1	30.778	BB	0.5171	1928.81580	57.53668	75.6153
2	35.308	BB	0.5933	622.01288	16.01026	24.3847
Totals :				2550.82867	73.54694	

Product 11a with Catalyst L-14

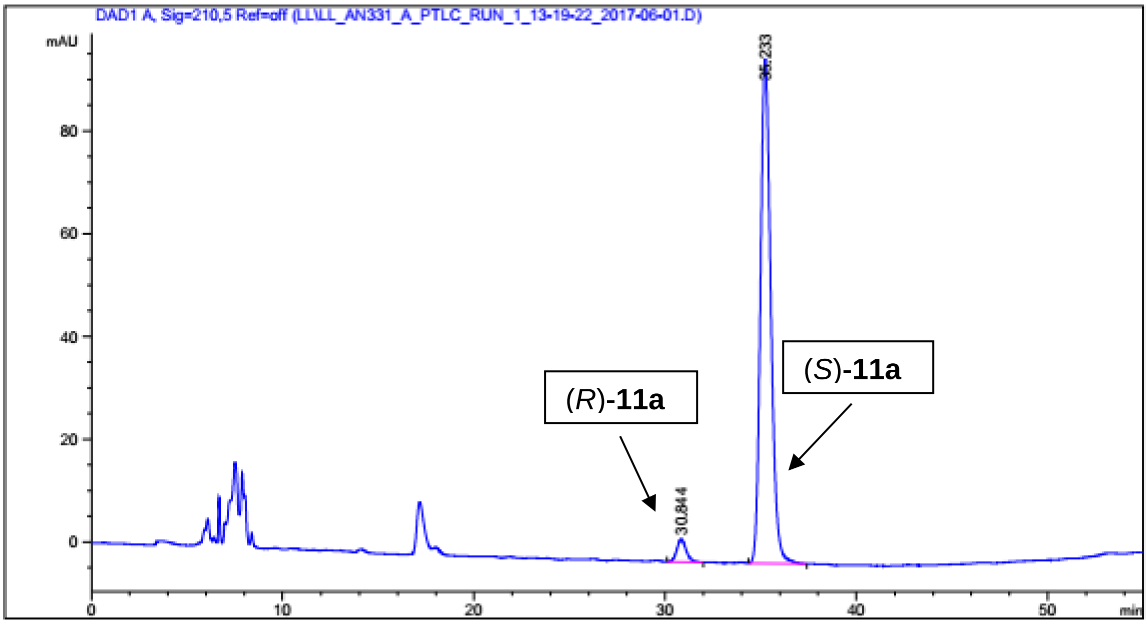

Signal 1: DAD1 A, Sig=210,5 Ref=off

Peak #	RetTime [min]	Type	Width [min]	Area [mAU*s]	Height [mAU]	Area %
1	30.844	BB	0.4957	152.03799	4.41519	3.9570
2	35.233	BB	0.5803	3690.23804	98.23528	96.0430

Totals : 3842.27603 102.65047

Product 11a with Catalyst Sav-7, Ethylacetate

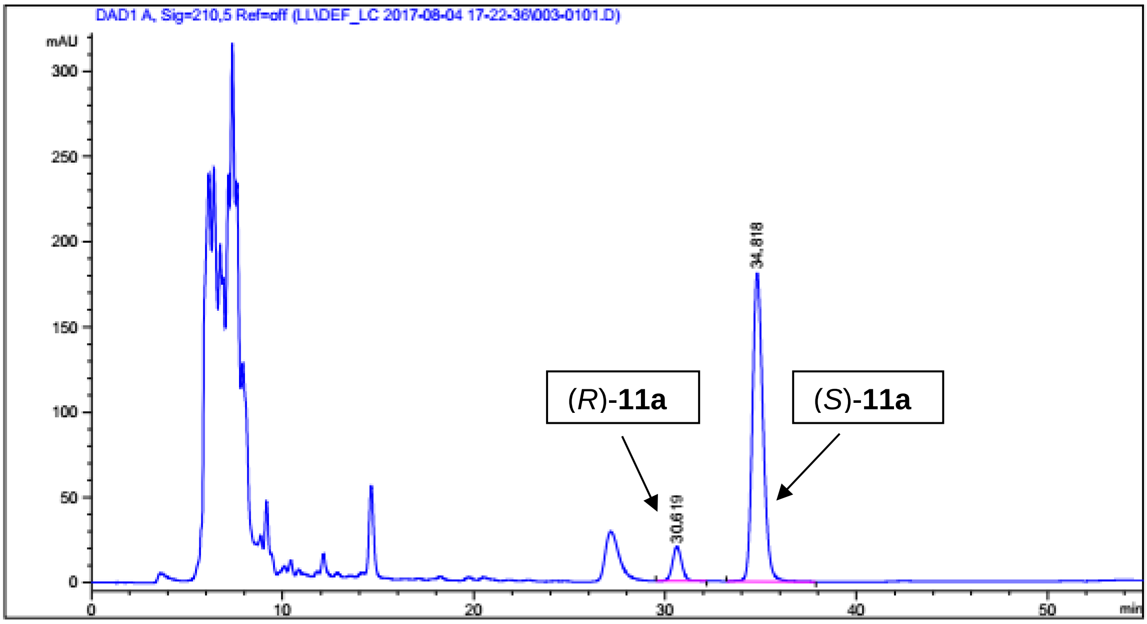

Signal 1: DAD1 A, Sig=210,5 Ref=off

Peak #	RetTime [min]	Type	Width [min]	Area [mAU*s]	Height [mAU]	Area %
1	30.619	BB	0.5165	683.22583	20.10131	8.9502
2	34.818	BB	0.5968	6950.39160	180.67255	91.0498

Totals : 7633.61743 200.77386

Product 11a with Catalyst Sav-7, 50% Methanol

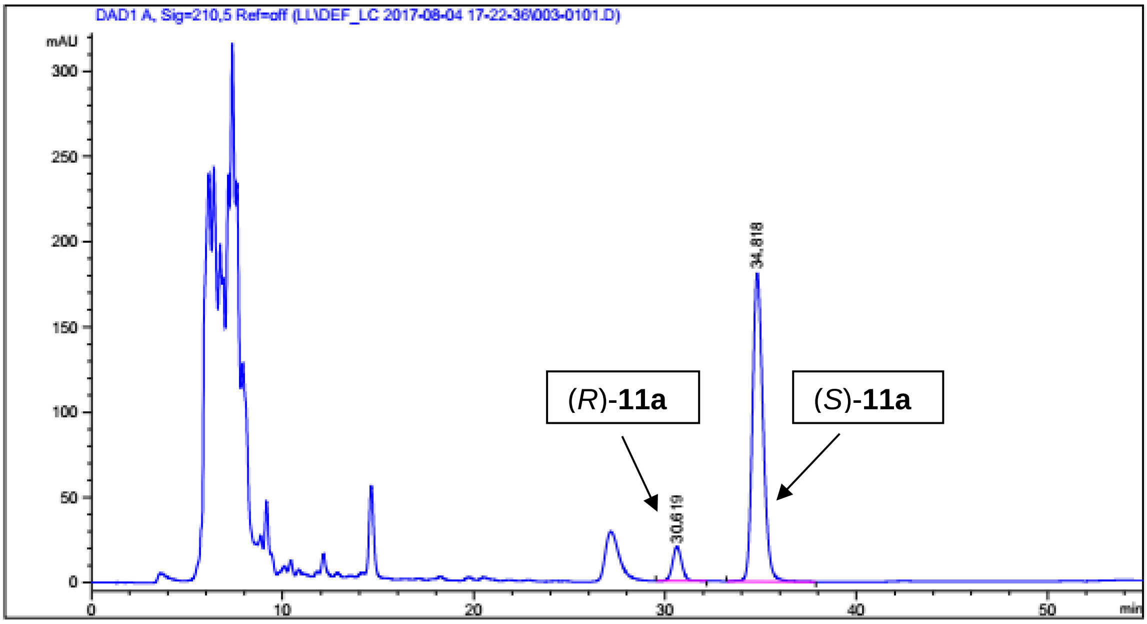

Signal 1: DAD1 A, Sig=210,5 Ref=off

Peak #	RetTime [min]	Type	Width [min]	Area [mAU*s]	Height [mAU]	Area %
1	30.619	BB	0.5165	683.22583	20.10131	8.9502
2	34.818	BB	0.5968	6950.39160	180.67255	91.0498

Totals : 7633.61743 200.77386

Product 11a with Catalyst Sav-7, 25% Methanol

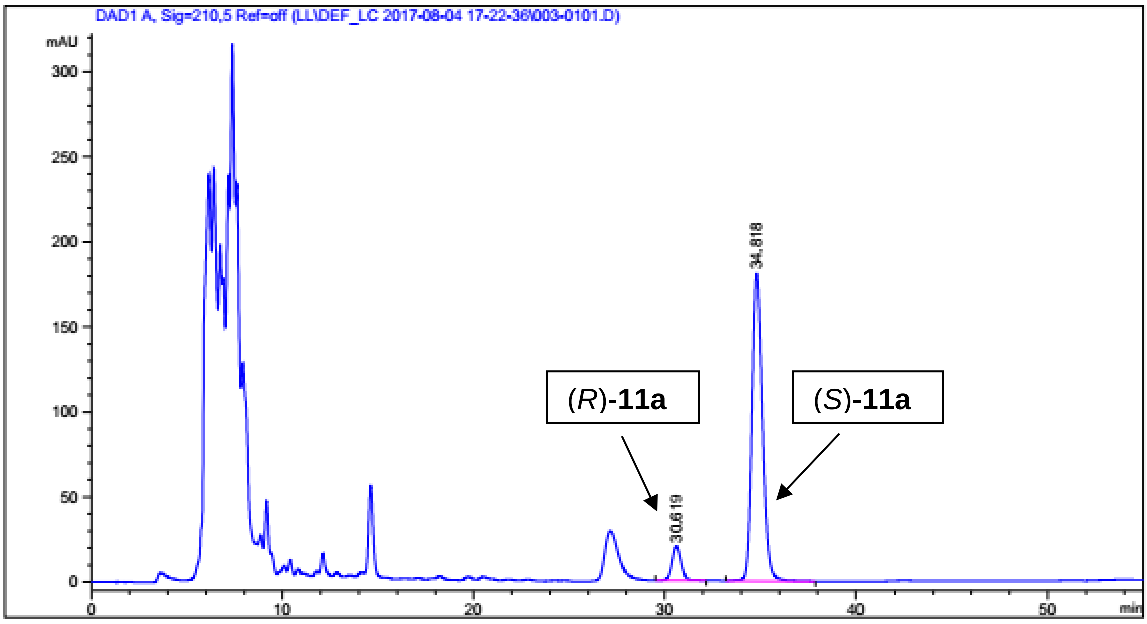

Signal 1: DAD1 A, Sig=210,5 Ref=off

Peak #	RetTime [min]	Type	Width [min]	Area [mAU*s]	Height [mAU]	Area %
1	30.619	BB	0.5165	683.22583	20.10131	8.9502
2	34.818	BB	0.5968	6950.39160	180.67255	91.0498

Totals : 7633.61743 200.77386

Product 11a with Catalyst Sav-7, 10% Methanol

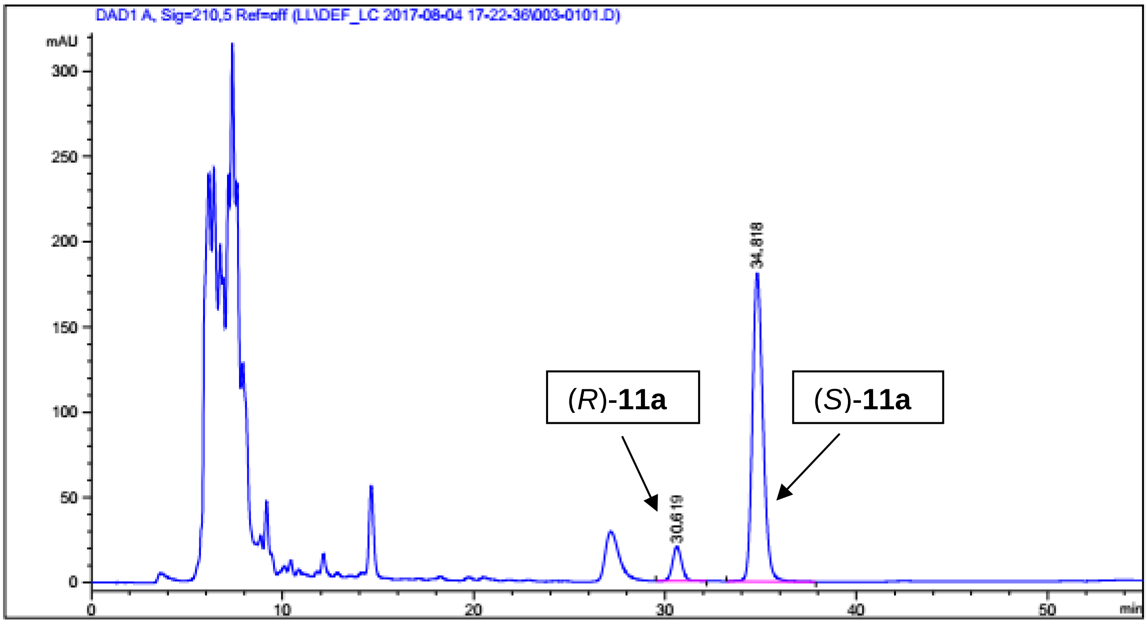

Signal 1: DAD1 A, Sig=210,5 Ref=off

Peak #	RetTime [min]	Type	Width [min]	Area [mAU*s]	Height [mAU]	Area %
1	30.619	BB	0.5165	683.22583	20.10131	8.9502
2	34.818	BB	0.5968	6950.39160	180.67255	91.0498

Totals : 7633.61743 200.77386

12.2 Cinnamaldehyde Derivatives under Optimized Conditions

Product 11b with Catalyst Sav-7, Methanol

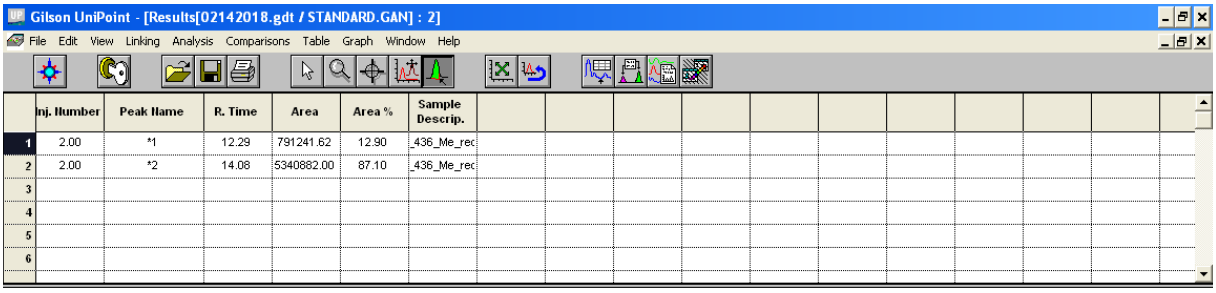

	Inj. Number	Peak Name	R. Time	Area	Area %	Sample Descrip.
1	2.00	*1	12.29	791241.62	12.90	_436_Me_rec
2	2.00	*2	14.08	5340882.00	87.10	_436_Me_rec
3						
4						
5						
6						

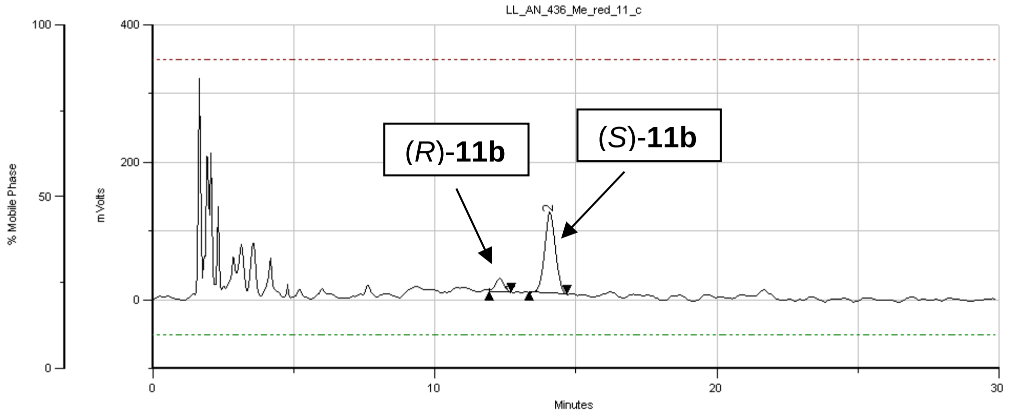

Conditions: 2.0 mL/min, 25 °C, *n*-hexane/*iso*-propanol 90:10

Racemate 11b

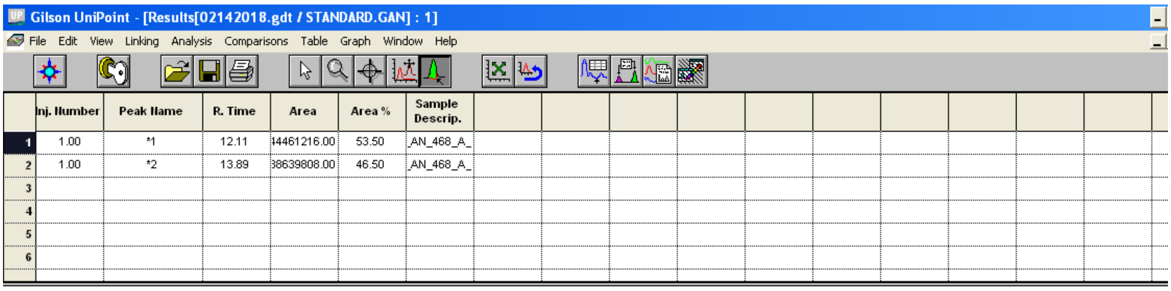

	Inj. Number	Peak Name	R. Time	Area	Area %	Sample Descrip.
1	1.00	*1	12.11	44461216.00	53.50	_AN_468_A_
2	1.00	*2	13.89	38639808.00	46.50	_AN_468_A_
3						
4						
5						
6						

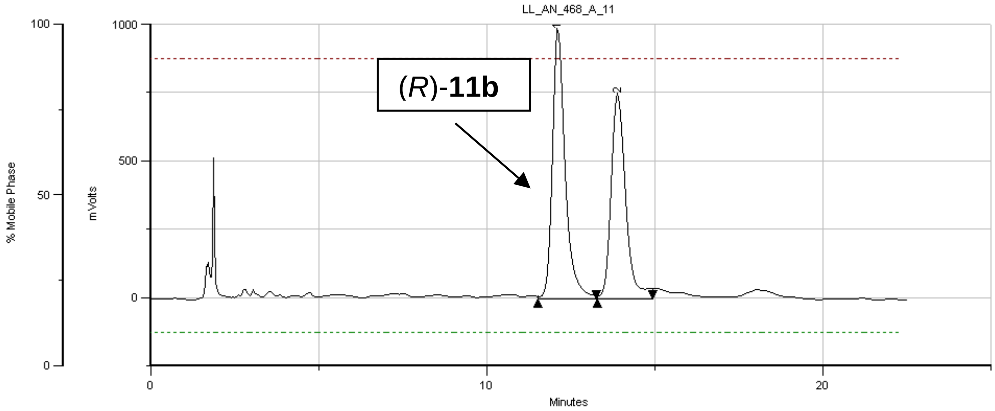

Conditions: 2.0 mL/min, 25 °C, *n*-hexane/*iso*-propanol 90:10

(*S*)-**11b**

Product 11c with Catalyst Sav-7, Methanol

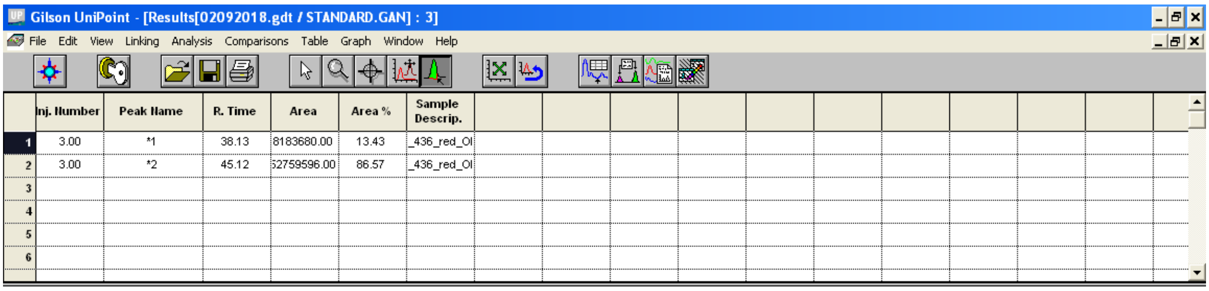

	Inj. Number	Peak Name	R. Time	Area	Area %	Sample Descrip.
1	3.00	*1	38.13	8183680.00	13.43	_436_red_Ol
2	3.00	*2	45.12	52759596.00	86.57	_436_red_Ol
3						
4						
5						
6						

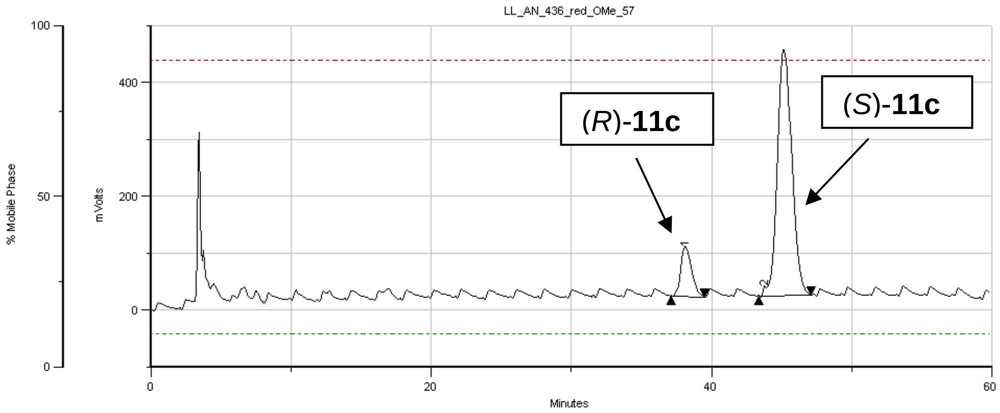

Conditions: 1.0 mL/min, 25 °C, *n*-hexane/*iso*-propanol 90:10

(*S*)-**11b**

Racemate 11c

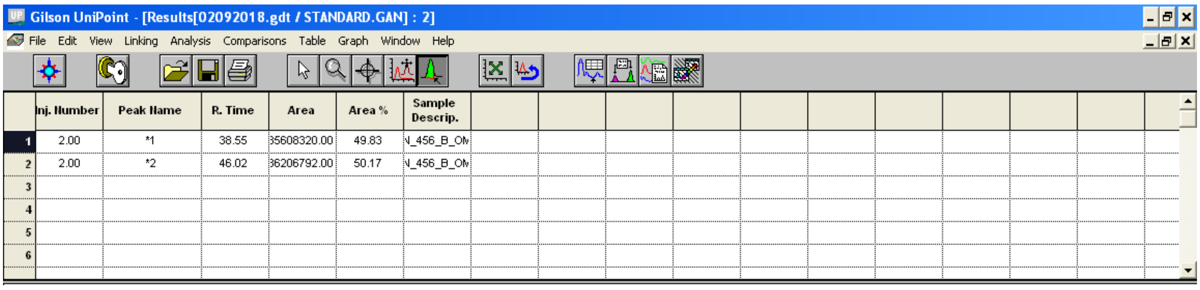
Gilson UniPoint - [Results[02092018.gdt / STANDARD.GAN] : 2]

File Edit View Linking Analysis Comparisons Table Graph Window Help

	Inj. Number	Peak Name	R. Time	Area	Area %	Sample Descrip.
1	2.00	*1	38.55	35608320.00	49.83	_456_B_OM
2	2.00	*2	46.02	36206792.00	50.17	_456_B_OM
3						
4						
5						
6						

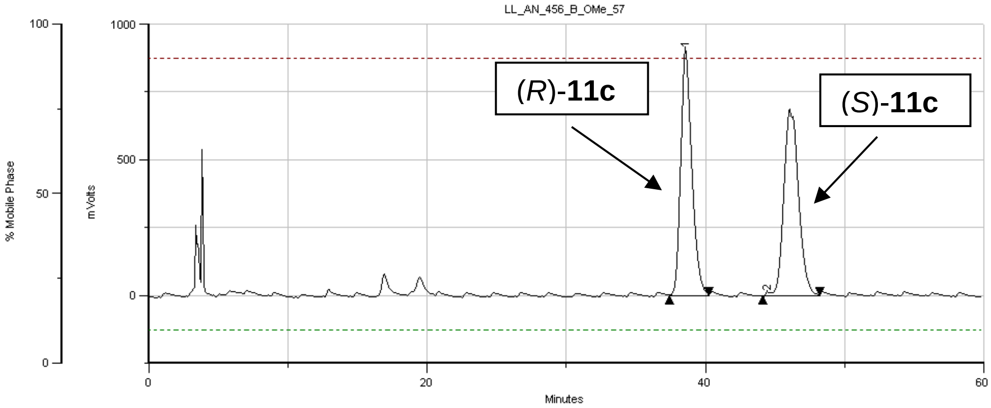

Conditions: 1.0 mL/min, 25 °C, *n*-hexane/*iso*-propanol 90:10

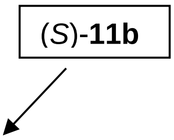

Product 11d with Catalyst Sav-7, Methanol

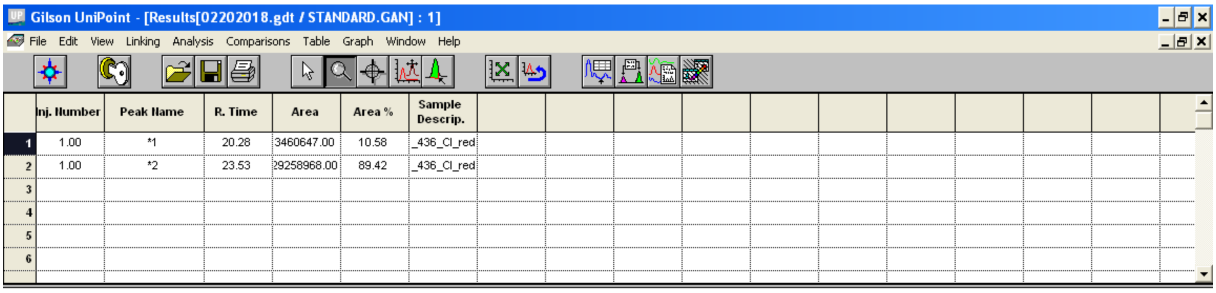

	Inj. Number	Peak Name	R. Time	Area	Area %	Sample Descrip.
1	1.00	*1	20.28	3460647.00	10.58	_436_Cl_red
2	1.00	*2	23.53	29258968.00	89.42	_436_Cl_red
3						
4						
5						
6						

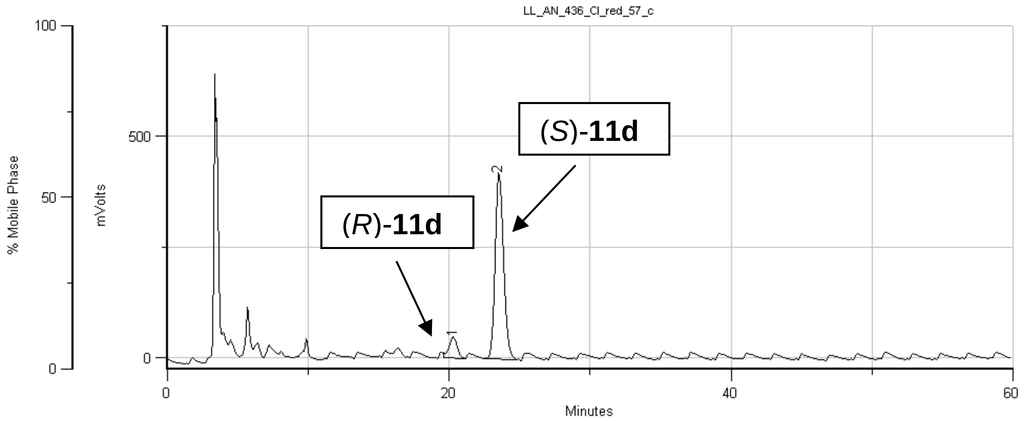

Conditions: 1.0 mL/min, 25 °C, *n*-hexane/*iso*-propanol 90:10

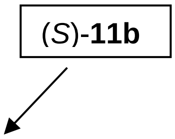

Racemate 11d

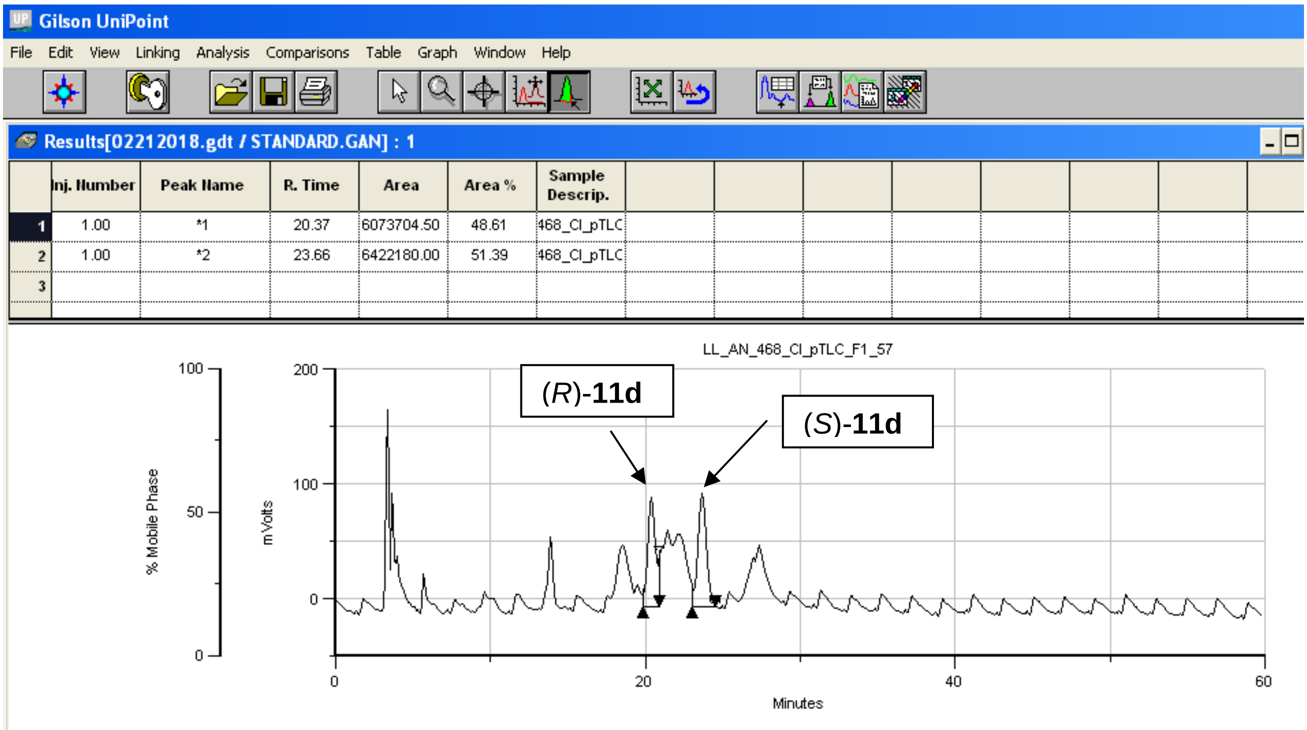

	Inj. Number	Peak Name	R. Time	Area	Area %	Sample Descrip.
1	1.00	*1	20.37	6073704.50	48.61	468_Cl_pTLC
2	1.00	*2	23.66	6422180.00	51.39	468_Cl_pTLC
3						

Conditions: 1.0 mL/min, 25 °C, *n*-hexane/*iso*-propanol 90:10

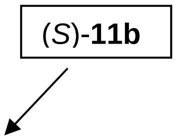

Product 11e with Catalyst Sav-7, Methanol

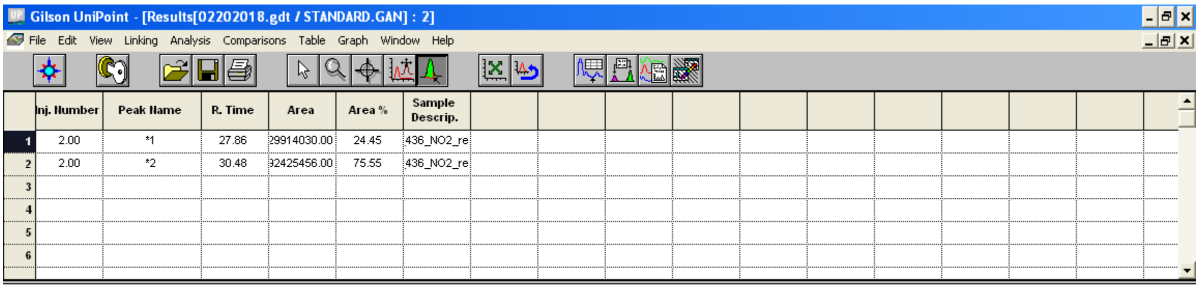

Gilson UniPoint - [Results[02202018.gdt / STANDARD.GAN] : 2]

File Edit View Linking Analysis Comparisons Table Graph Window Help

	Inj. Number	Peak Name	R. Time	Area	Area %	Sample Descrip.
1	2.00	*1	27.86	29914030.00	24.45	436_NO2_re
2	2.00	*2	30.48	92425456.00	75.55	436_NO2_re
3						
4						
5						
6						

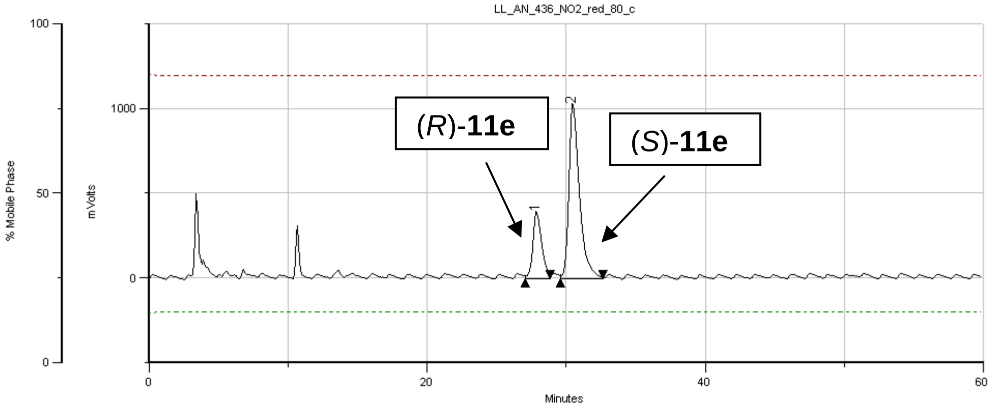

Conditions: 1.0 mL/min, 25 °C, *n*-hexane/*iso*-propanol 85:15

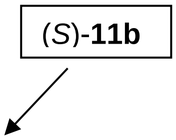

Racemate 11e

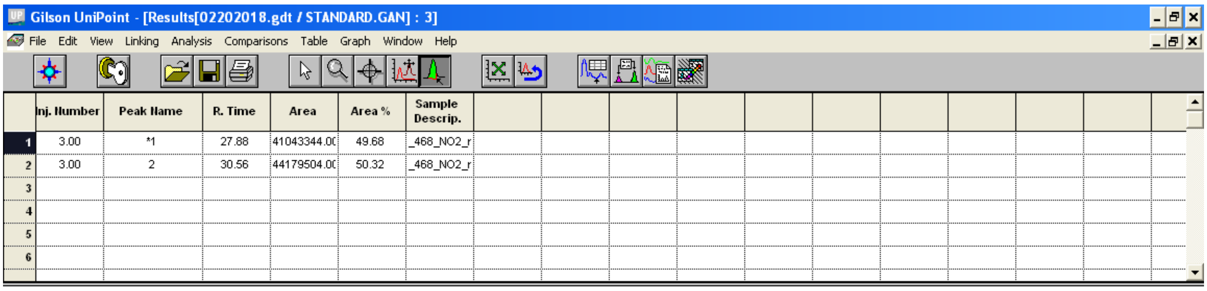

	Inj. Number	Peak Name	R. Time	Area	Area %	Sample Descrip.
1	3.00	*1	27.88	41043344.0[illegible]	49.68	_468_NO2_r
2	3.00	2	30.56	44179504.0[illegible]	50.32	_468_NO2_r
3						
4						
5						
6						

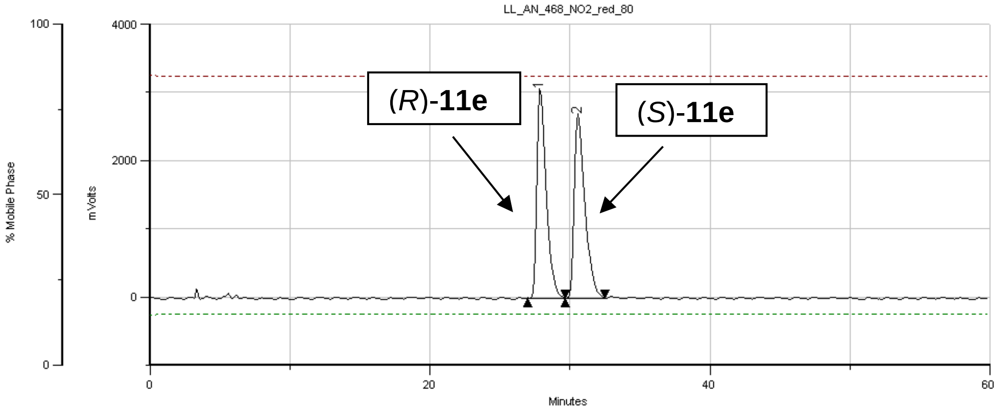

Conditions: 1.0 mL/min, 25 °C, *n*-hexane/*iso*-propanol 85:15

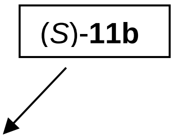

13 References

[1] E. Rodrigo, J. L. Garcia Ruano, M. B. Cid, *J. Org. Chem.* **2013**, *78*, 10737-10746.
[2] J. L. Shih, T. S. Nguyen, J. A. May, *Angew. Chem. Int. Ed.* **2015**, *54*, 9931-9935; *Angew. Chem.* **2015**, *127*, 10069-10073.
[3] C. Palomo, A. Landa, A. Mielgo, M. Oiarbide, A. Puente, S. Vera, *Angew. Chem. Int. Ed.* **2007**, *46*, 8431-8435; *Angew. Chem.* **2007**, *119*, 8583-8587; .
[4] I. Mager, K. Zeitler, *Org. Lett.* **2010**, *12*, 1480-1483.
[5] G. R. Fulmer, A. J. M. Miller, N. H. Sherden, H. E. Gottlieb, A. Nudelman, B. M. Stoltz, J. E. Bercaw, K. I. Goldberg, *Organometallics* **2010**, *29*, 2176-2179.
[6] T. L. Foley, A. Yasgar, C. J. Garcia, A. Jadhav, A. Simeonov, M. D. Burkart, *Org. Biomol. Chem.* **2010**, *8*, 4601-4606.
[7] Z. Yuan, Z. Que, S. Cheng, R. Zhuo, F. Li, *Chem. Commun.* **2012**, *48*, 8129-8131.
[8] L. Samulis, N. C. O. Tomkinson, *Tetrahedron* **2011**, *67*, 4263-4267.
[9] (a) L. Baugh, I. Le Trong, D. S. Cerutti, S. Gulich, P. S. Stayton, R. E. Stenkamp, T. P. Lybrand, *Biochemistry* **2010**, *49*, 4568-4570; (b) I. Le Trong, Z. Wang, D. E. Hyre, T. P. Lybrand, P. S. Stayton, R. E. Stenkamp, *Acta Crystallogr., Sect. D: Biol. Crystallogr.* **2011**, *67*, 813-821; (c) L. Baugh, I. Le Trong, D. S. Cerutti, N. Mehta, S. Gulich, P. S. Stayton, R. E. Stenkamp, T. P. Lybrand, *Biochemistry* **2012**, *51*, 597-607; (d) S. Freitag, I. Le Trong, A. Chilkoti, L. A. Klumb, P. S. Stayton, R. E. Stenkamp, *J. Mol. Biol.* **1998**, *279*, 211-221.
[10] (a) W. Kabsch, *Acta Crystallogr., Sect. D: Biol. Crystallogr.* **2010**, *66*, 125-132; (b) W. Kabsch, *Acta Crystallogr., Section D: Biol. Crystallogr.* **2010**, *66*, 125-132.
[11] P. R. Evans, G. N. Murshudov, *Acta Crystallogr., Sect. D: Biol. Crystallogr.* **2013**, *69*, 1204-1214.
[12] A. A. Lebedev, A. A. Vagin, G. N. Murshudov, *Acta Crystallogr., Sect. D: Biol. Crystallogr.* **2008**, *64*, 33-39.
[13] M. D. Winn, C. C. Ballard, K. D. Cowtan, E. J. Dodson, P. Emsley, P. R. Evans, R. M. Keegan, E. B. Krissinel, A. G. W. Leslie, A. McCoy, S. J. McNicholas, G. N. Murshudov, N. S. Pannu, E. A. Potterton, H. R. Powell, R. J. Read, A. Vagin, K. S. Wilson, *Acta Crystallogr. D* **2011**, *67*, 235-242.
[14] G. N. Murshudov, P. Skubak, A. A. Lebedev, N. S. Pannu, R. A. Steiner, R. A. Nicholls, M. D. Winn, F. Long, A. A. Vagin, *Acta Crystallogr., Sect. D: Biol. Crystallogr.* **2011**, *67*, 355-367.
[15] P. Emsley, B. Lohkamp, W. G. Scott, K. Cowtan, *Acta Crystallogr., Sect. D: Biol. Crystallogr.* **2010**, *66*, 486-501.
[16] P. C. Weber, D. H. Ohlendorf, J. J. Wendoloski, F. R. Salemme, *Science* **1989**, *243*, 85-88.
[17] E. Krissinel, K. Henrick, *J. Mol. Biol.* **2007**, *372*, 774-797.
[18] R. Gautier, A. C. Camproux, P. Tuffery, *Nucleic Acids Res.* **2004**, *32*, W508-511.
[19] C. R. Sondergaard, M. H. Olsson, M. Rostkowski, J. H. Jensen, *J. Chem. Theory Comput.* **2011**, *7*, 2284-2295.
[20] T. J. Dolinsky, J. E. Nielsen, J. A. McCammon, N. A. Baker, *Nucleic Acids Res.* **2004**, *32*, W665-667.
[21] J. C. Phillips, R. Braun, W. Wang, J. Gumbart, E. Tajkhorshid, E. Villa, C. Chipot, R. D. Skeel, L. Kale, K. Schulten, *J. Comput. Chem.* **2005**, *26*, 1781-1802.
[22] Y. Duan, C. Wu, S. Chowdhury, M. C. Lee, G. Xiong, W. Zhang, R. Yang, P. Cieplak, R. Luo, T. Lee, J. Caldwell, J. Wang, P. Kollman, *J. Comput. Chem.* **2003**, *24*, 1999-2012.
[23] S. E. Feller, Y. Zhang, R. W. Pastor, B. R. Brooks, *J. Chem. Phys.* **1995**, *103*, 4613-4621.
[24] (a) J. Wang, R. M. Wolf, J. W. Caldwell, P. A. Kollman, D. A. Case, *J Comput Chem* **2004**, *25*, 1157-1174; (b) J. Wang, W. Wang, P. A. Kollman, D. A. Case, *J Mol Graph Model* **2006**, *25*, 247-260.
